# Structural Analysis of dsRNA Binding to Anti-viral Pattern Recognition Receptors LGP2 and MDA5

**DOI:** 10.1016/j.molcel.2016.04.021

**Published:** 2016-05-19

**Authors:** Emiko Uchikawa, Mathilde Lethier, Hélène Malet, Joanna Brunel, Denis Gerlier, Stephen Cusack

**Affiliations:** 1 European Molecular Biology Laboratory, Grenoble Outstation, 71 Avenue des Martyrs, CS 90181, 38042 Grenoble Cedex 9, France; 2 University Grenoble Alpes, Centre National de la Recherche Scientifique, EMBL Unit of Virus Host-Cell Interactions, 71 Avenue des Martyrs, CS 90181, 38042 Grenoble Cedex 9, France; 3 CIRI, International Center for Infectiology Research, Université de Lyon, 69007 Lyon, France; 4 Inserm, U1111, 69007 Lyon, France; 5 CNRS, UMR5308, 69007 Lyon, France; 6 Ecole Normale Supérieure de Lyon, 69007 Lyon, France; 7 Université Lyon 1, Centre International de Recherche en Infectiologie, 69007 Lyon, France

## Abstract

RIG-I and MDA5 sense virus-derived short 5′ppp blunt-ended or long dsRNA, respectively, causing interferon production. Non-signaling LGP2 appears to positively and negatively regulate MDA5 and RIG-I signaling, respectively. Co-crystal structures of chicken (*ch*) LGP2 with dsRNA display a fully or semi-closed conformation depending on the presence or absence of nucleotide. LGP2 caps blunt, 3′ or 5′ overhang dsRNA ends with 1 bp longer overall footprint than RIG-I. Structures of 1:1 and 2:1 complexes of *ch*MDA5 with short dsRNA reveal head-to-head packing rather than the polar head-to-tail orientation described for long filaments. *ch*LGP2 and *ch*MDA5 make filaments with a similar axial repeat, although less co-operatively for *ch*LGP2. Overall, LGP2 resembles a chimera combining a MDA5-like helicase domain and RIG-I like CTD supporting both stem and end binding. Functionally, RNA binding is required for LGP2-mediated enhancement of MDA5 activation. We propose that LGP2 end-binding may promote nucleation of MDA5 oligomerization on dsRNA.

## Highlights

• *ch*LPG2-dsRNA structures reveal RIG-I like end binding, but overhangs are possible
• *ch*MDA5-dsRNA complex structures show head-to-head packing on short dsRNAs
• LGP2 also has MDA5-like behavior, coating dsRNA but with less cooperativity
• Both human and chicken LGP2 enhance MDA5 signaling in an RNA-dependent manner

## Graphical Abstract

**Figure:**
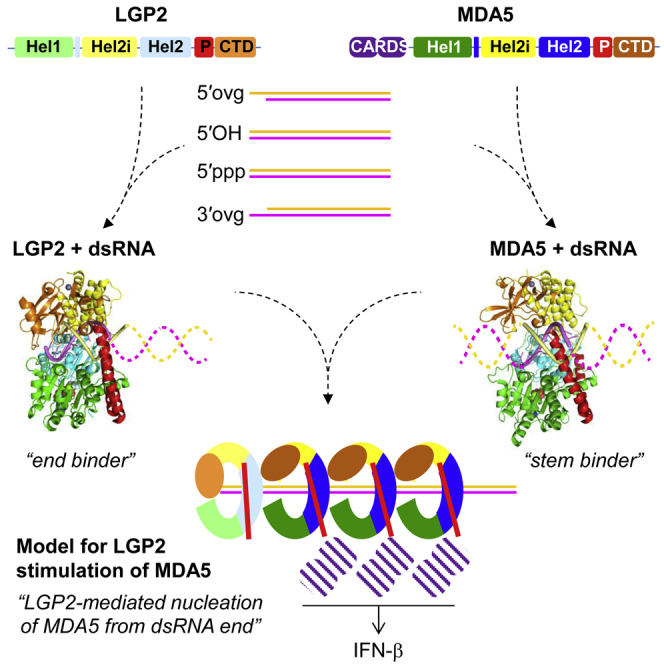

## Teaser

Uchikawa et al. reveal structural details of dsRNA recognition by MDA5 and LGP2 that synergistically sense viral RNA and activate interferon expression. LGP2 is primarily a dsRNA end binder but can also coat dsRNA, but less co-operatively than MDA5. Functional studies show that LGP2 enhancement of MDA5 signaling is RNA dependent.

## Introduction

**Figure 1:**
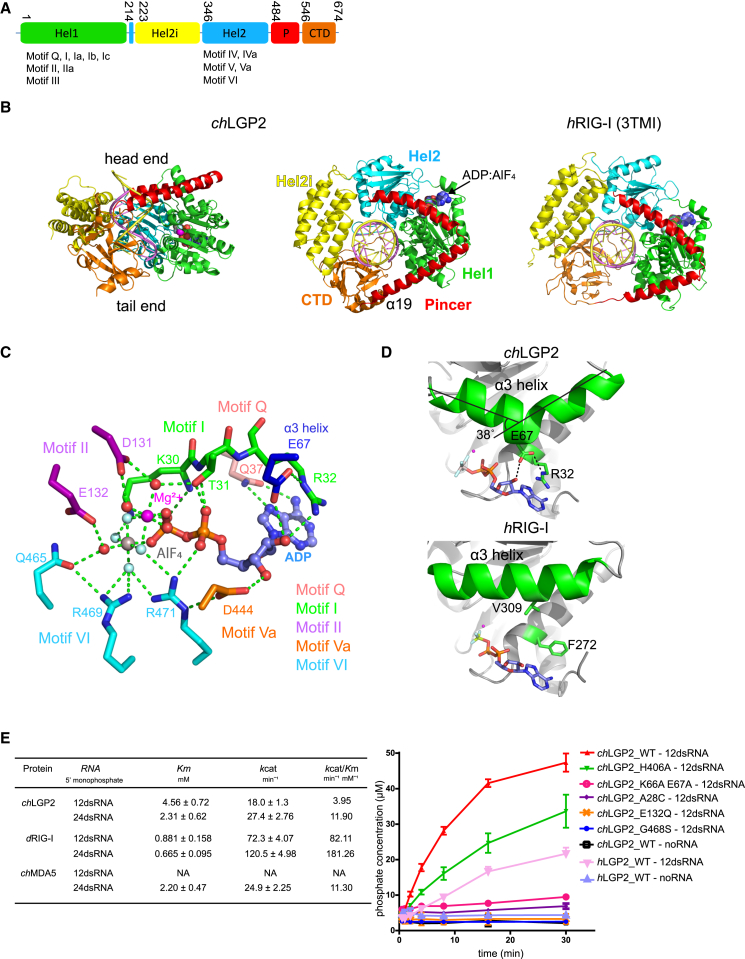
Overall Structure of *ch*LGP2-dsRNA-ADP:AlF_4_ Complex (A) Domain structure of *ch*LGP2. Domain colors are green (Hel1), yellow (Hel2i), cyan (Hel2), red (pincer motif), and orange (CTD). (B) Side and head-end cartoon view of *ch*LGP2-dsRNA-ADP:AlF_4_ complex (left and middle) compared with *h*RIG-I-dsRNA-ADP:BeF_3_ complex (PDB: 5E3H [3TMI]) (right). In the side view, the head end contains the pincer motif and the tail end contains the CTD. Domain colors are as in (A), with the zinc atom in the CTD a black sphere. The dsRNA 3′ and 5′ strands are, respectively, violet and yellow. The ADP:AlF_4_ is in spheres representation. Note that the second pincer domain helix (α19) of *ch*LGP2 extends right up to the CTD, unlike in *h*RIG-I. (C) Details of the immediate protein ligands of the ADP:AlF_4_:Mg^2+^ bound in *ch*LGP2, all of which (except Glu67) come from helicase motifs Q, I and II (Hel1), and Va and VI (Hel2), which are colored as indicated. The Mg^2+^ ion, aluminum, and fluorine atoms are, respectively, purple, gray, and light blue spheres. Glu132 and Gln465 coordinate the mimic of the attacking water molecule (red sphere) in this transition-state analog complex. (D) Diagram showing how in *ch*LGP2, Glu67 emerges from helix *a*3 to be involved in ATP ribose binding and stabilization of Arg32, which stacks on the adenine base (top). A very similar situation occurs in MDA5 (see Figure 4D) but not RIG-I (bottom). (E) Left: table of *K*_m_ and *k*cat values for the RNA-dependent ATPase activity of wild-type *ch*LGP2, *d*RIG-I, and *ch*MDA5. Right: representative ATP hydrolysis curves for *ch*LGP2 and *h*LGP2 with without dsRNA and various mutations of *ch*LGP2. See also Figure S2. See also Figures S1–S3.

Homologous double-stranded RNA (dsRNA) dependent ATPases RIG-I, MDA5, and LGP2 (RIG-I-like helicases [RLHs]) are key cytosolic pattern recognition receptors in the vertebrate innate immune response against RNA viruses. RIG-I senses primarily 5′ppp blunt-end dsRNA (5′ppp-dsRNA), whereas MDA5 is activated by long dsRNA. Consequently, the two sensors respond to different but overlapping sets of viruses (Yoo et al., 2014). Both activated receptors trigger the same downstream signaling pathway, leading to interferon (IFN) induction (Goubau et al., 2013). RIG-I and MDA5 possess tandem N-terminal caspase activation and recruitment domains (CARDs), a central DECH-box helicase domain (Fairman-Williams et al., 2010), and a C-terminal domain (CTD). LGP2 differs in lacking CARDs (Figure 1A) and thus independent signaling activity.

Extensive studies of RIG-I and MDA5 have elucidated their mode of RNA binding and activation and signaling mechanisms (Ahmad and Hur, 2015, Hopfner, 2014). RIG-I binding to short 5′ppp-dsRNA via its CTD and helicase domains releases the CARDs from an auto-inhibitory state, in an ATP-dependent manner (Kowalinski et al., 2011). This allows them to interact with and oligomerize, in a poly-ubiquitin-dependent fashion, downstream signaling partner MAVS (Peisley et al., 2014, Wu et al., 2014). By contrast, MDA5 binds co-operatively to long dsRNA to form protein-coated filaments (Berke et al., 2012, Peisley et al., 2011, Wu et al., 2013); the resulting oligomerization of MDA5 CARDs activates MAVS (Wu et al., 2013).

LGP2 is reported to be both a positive and negative regulator of the anti-viral response (Rodriguez et al., 2014, Zhu et al., 2014). A positive role for LGP2 in anti-viral signaling is supported by the higher susceptibility of LGP2 knockout mice to certain RNA viruses (Satoh et al., 2010) and co-operative activity with MDA5 (Childs et al., 2013). Indeed, small amounts of LGP2 enhance MDA5-mediated signaling (Bruns et al., 2014), and a picornavirus-derived MDA5 agonist was found through its interaction with LGP2 (Deddouche et al., 2014). Furthermore, LGP2, like MDA5, is specifically targeted for inactivation by paramxyovirus V protein (Childs et al., 2012, Rodriguez and Horvath, 2013). A negative role for LGP2 emerged from inhibitory activity observed upon overexpression of LGP2 (Bruns et al., 2013, Liniger et al., 2012, and references therein).

To gain further insight into the role of LGP2 in the anti-viral response and its co-operative role in MDA5 signaling, we performed structural, biochemical, and cell-based studies on chicken (*ch*) LGP2 and MDA5. Interestingly, chicken (Barber et al., 2010) and another *Galliforme*, turkey (according to its draft genome), both lack a RIG-I gene, unlike other vertebrates, including many birds (Chen et al., 2013). Nevertheless, chicken cells express MDA5, LGP2, and MAVS (Karpala et al., 2011, Liniger et al., 2012) and produce type I IFN in response to highly pathogenic avian influenza virus, most likely via *ch*MDA5 in co-operation with *ch*LGP2 (Hayashi et al., 2014, Liniger et al., 2012). We present the crystal structure of *ch*LGP2 bound to 5′ mono-phosphate (5′p), 5′ tri-phosphate (5′ppp), and 3′ overhang dsRNA at, respectively, 1.5, 2.2, and 2.0 Å resolution and characterize the RNA binding and ATPase activity of *ch*LGP2 and human (*h*) LGP2. We also report crystal structures of 1:1 and 2:1 CARD-deleted *ch*MDA5-dsRNA complexes at, respectively, 2.60 and 2.75 Å resolution. We demonstrate by electron microscopy (EM) that both *ch*MDA5 and *ch*LGP2 make filaments with dsRNA with the same axial repeat. Finally, functional studies reveal that LGP2 enhanced poly(I:C)-dependent MDA5 signaling, in both chicken and human cells, requires an intact RNA binding site on both the LGP2 helicase and CTD domains.

## Results

### Overall Structure of *ch*LGP2

We determined three co-crystal structures of full-length *ch*LGP2 with the ATP transition state analog adenosine 5′-diphosphate:aluminum fluoride (ADP:AlF_4_) and either a 10-mer palindromic 5′p dsRNA (*ch*LGP2_10p) or 5′ppp dsRNA (*ch*LGP2_10ppp) or a 5′ppp and 3′ two nucleotide (GG) overhang hairpin RNA duplex (*ch*LGP2-3ovg) at, respectively, 1.5, 2.2, and 2.0 Å resolution. A fourth structure of *ch*LGP2 bound to a 12-mer palindromic dsRNA has no bound nucleotide. See Table 1 for crystallographic details.

Overall, *ch*LGP2 resembles other dsRNA-bound RLHs with the two RecA-like helicase domains (Hel1, Hel2), helicase insertion domain (Hel2i), pincer domain (P), and CTD wrapping around the dsRNA stem. Hel1 contains the conserved helicase motifs Q, I, Ia, Ib, Ic, II, IIa, and III, whereas Hel2 contains motifs IV, IVa, V, Va, and VI (Figures 1A and 1B; see also Figure S1 for the secondary structure of *ch*LGP2 and sequence alignment with other RLHs). The unusually high resolution of the structures reveals details of the ADP:AlF_4_ binding site and the highly hydrated *ch*LGP2-dsRNA interface. One distinctive feature of LGP2 is that the second pincer domain helix (α19) has eight turns and connects directly to the CTD, whereas in RIG-I, α19 has only six turns, followed by an extended, proline-rich connecting peptide (Figure 1B). Thus in LGP2, the CTD-pincer linkage appears to be more constrained and lacking the functionally important flexibility observed in RNA free RIG-I (Kowalinski et al., 2011). The structure of *ch*LGP2-dsRNA-ADP:AlF_4_ complex is in a highly ordered, closed conformation, mimicking the transition state for ATP hydrolysis, and thus most closely resembles the duck RIG-IΔCTD-dsRNA-ADP:AlF_4_ complex (*d*RIG-I; Protein Data Bank [PDB]: 4A36) (Kowalinski et al., 2011) in the disposition of the domains, except that the CTD is present as well. In the *ch*LGP2 structure without nucleotide, the helicase is in a semi-closed state with Hel2 separated from Hel1 and partially disordered. See the Discussion for an analysis of the different nucleotide-dependent conformations observed for RLHs.

### ADP:AlF_4_ Binding and ATP Hydrolysis Activity of *ch*LGP2

ADP:AlF_4_ is tightly bound at the interface between the *ch*LGP2 Hel1 and Hel2 with conserved motifs Q, I, and II from Hel1 and motifs Va and VI from Hel2 engaged in the interaction (Figures 1C and S2A). The adenosine base is partially stacked between His4 (motif Q) and LGP2 conserved Arg32 (motif I), and base-specific interactions are provided by the main-chain carbonyl of Glu2 and side chain of Gln7 (motif Q). This differs from RIG-I, where the base stacks between motif Q Arg244 (*h*RIG-I) and motif I Phe272 (equivalent to His4 and Arg32 in LGP2, respectively). The ADP ribose hydrogen bonds with conserved Glu67, which itself is stabilized by a salt bridge with Arg32 (Figures 1C and 1D). Glu67 emerges from a kink in helix α3 (Figure 1D), a feature that is conserved in MDA5 (see below; Figure S1), but not RIG-I, where the equivalent helix is straight and not involved in nucleotide binding (Figure 1D). Motif I (25-PTGAGKTR-32) wraps around the α- and β-phosphates of the ADP, making several hydrogen bonds. Motif Va (440-EEGLD-444) and motif VI (467-QGRARA-472) directly interact with ADP:AlF_4,_ a characteristic of the closed form. Motif Va Asp444 interacts with the ADP ribose and stabilizes Arg471 of motif VI. Arg471 interacts with the α-phosphate and two fluorines of AlF_4_. Arg469, stabilized by Gln465, interacts with a third fluorine, mimicking stabilization of the transition state (Figures 1C and S2B). The high-resolution structure reveals the complex hydrogen bonding network involving Asp131 and Glu132 (motif II), AlF_4_, Mg^2+^, and water molecules in the presumed transition state. The octahedrally coordinated Mg^2+^ ion ligates one oxygen atom of the β-phosphate, two fluorine atoms, and three water molecules. Two magnesium coordinated water molecules form direct contacts with the side chains of Thr31 (motif I) and Asp131 (motif II) (Figures 1C and S2B). One water molecule coordinated by Glu132 (motif II, DECH) and Gln465 (motif VI) likely represents the attacking nucleophilic water, which catalyzes ATP hydrolysis (Figures 1C and S2B).

According to Bruns et al. (2013), *h*LGP2 has significant basal (dsRNA-independent) ATPase activity. We find that both *ch*LGP2 and *h*LGP2 ATPase activity is strictly dsRNA dependent (Figure 1E). Unlike MDA5, the activity is not dsRNA length dependent (Figures S2C and S2D). Moreover the ATP catalytic efficiency, as indicated by *k*cat/*K*m, is much lower for both *ch*LGP2 and *ch*MDA5 than for *d*RIG-I (Figures 1E and S2C). The importance of the interaction of Glu67 with the nucleotide ribose is underlined by the abolished ATPase activity of a *ch*LGP2 K66A/E67A mutant (Figure 1E). Within the highly conserved motif I sequence (25-PTGAGKTR-32, *ch*LGP2) the fourth position differs, being C/S in RIG-I, S in MDA5, and A/G/S in LGP2 (Figure S2E), and an A28C mutation abolishes the ATPase activity of *ch*LGP2 (Figure 1E). Glu132 (motif II) (Civril et al., 2011, Louber et al., 2015) and Gly468 (motif VI; Figure S2F) (Funabiki et al., 2014) are absolutely conserved and crucial for ATP hydrolysis by RIG-I and MDA5, correlating with their mutation underlying genetic disease (Funabiki et al., 2014, Jang et al., 2015). Accordingly, E132Q or G468S mutations abolish the ATPase activity of *ch*LGP2 (Figure 1E).

### Recognition of dsRNA by *ch*LGP2

**Figure 2:**
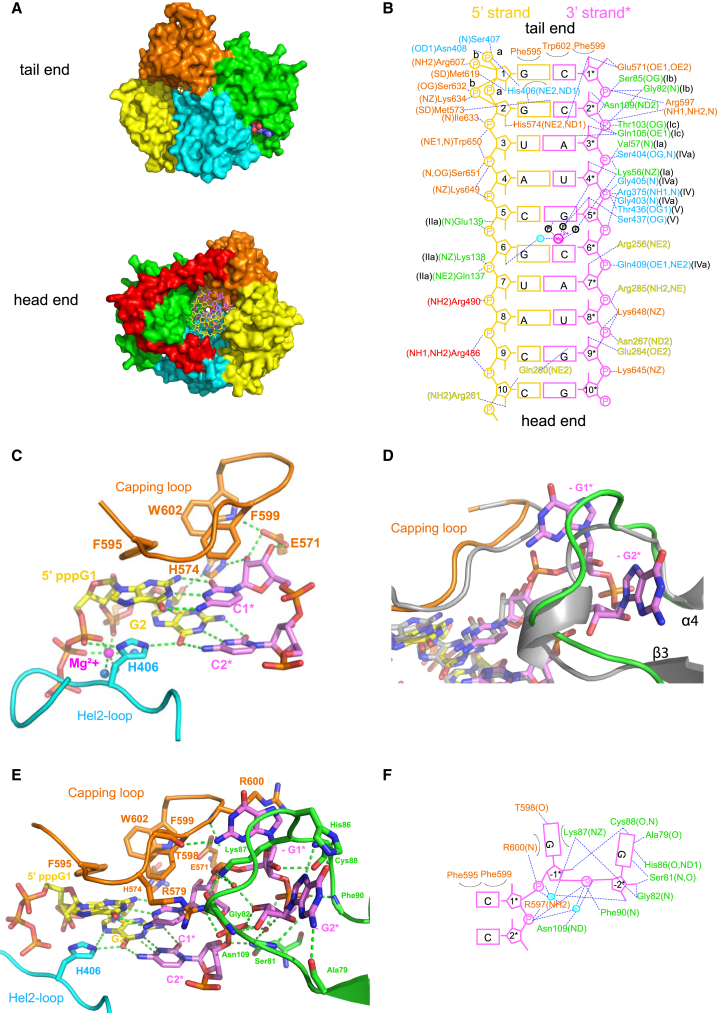
dsRNA Binding by *ch*LGP2 (A) Surface representation of the *ch*GP2-dsRNA-ADP:AlF_4_ complex viewed from the tail (left) and head (right) ends, colored as in Figure 1. At the tail end, the protein completely caps the blunt end of the dsRNA, whereas at the head end, the dsRNA can be extended. (B) Schematic diagram showing the interactions of *ch*LGP2 residues with the 10-mer dsRNA. Residues are colored according to domain and labeled with the conserved motif they belong to and the atom involved in the interaction. Polar interactions are indicated with a blue dotted line (cutoff 3.5 Å) and hydrophobic interactions by an arc. Numerous direct or water-mediated interactions are omitted for clarity. The 3′ strand nucleotides are numbered with an asterisk (i.e., 5′-G1:C1^∗^-3′ is the first base pair from the tail end). The two observed alternative conformations of the first and second 5′ phosphates are shown. (C) Details of the *ch*LGP2-RNA interactions that cap the blunt end of the 5′ppp dsRNA showing the role of aromatic residues Phe595, Phe599, and Trp602 from the capping loop, Glu571 and His574 from the 3′ end-binding loop, and His406 from the Hel2-loop. The Mg^2+^ coordinated by the 5′ppp is a magenta sphere. (D) Comparison of *ch*LGP2 structures with 5′ppp-dsRNA without (gray) or with (colors) a 3′-GG overhang, showing how a slight displacement of the capping loop and rearrangement of the β3-α4 loop accommodates the 3′ end extrusion. (E) Schematic diagram showing interactions with the 3′-GG overhang nucleotides (denoted G-1^∗^ and G-2^∗^), annotated as in (B). (F) Structural details of the network of interactions between *ch*LGP2 and the 3′-GG overhang nucleotides. Compared with (C), Glu571 and His574 no longer interact with C1^∗^ ribose. See also Figures S4 and S5.

Because of the full closure of all domains around the dsRNA (Figure 2A), many residues of *ch*LGP2 make direct interactions to the dsRNA. These are detailed in Figure 2B, in which numerous water-mediated protein-RNA or direct water-RNA interactions are omitted for clarity. The protein-RNA interface buries a total solvent accessible surface of 4,700 Å^2^ (10p) or 4,786 Å^2^ (10ppp), as compared with 3,109 Å^2^ for *h*RIG-I in the semi-closed state (PDB: 5E3H [3TMI]). RNA binding residues come from the CTD and the conserved motifs Ia, Ib, Ic, and IIa from Hel1 and IV, IVa, and V from Hel2. In addition, Hel2i and the pincer domain interact with the dsRNA. Hel2i residues from helix α10 (Gln256, Gln260, Arg261, Glu264, and Asn267) as well as Arg285 from helix α11 interact with both dsRNA strands via the minor groove. Arg486 and Arg490 from the long helix α18 of the pincer domain also interact directly with the phosphate backbone of the dsRNA 5′ strand. The above-cited interacting residues are conserved or conservatively substituted polar residues in LGP2 from different organisms (Figure S3).

**Figure 3:**
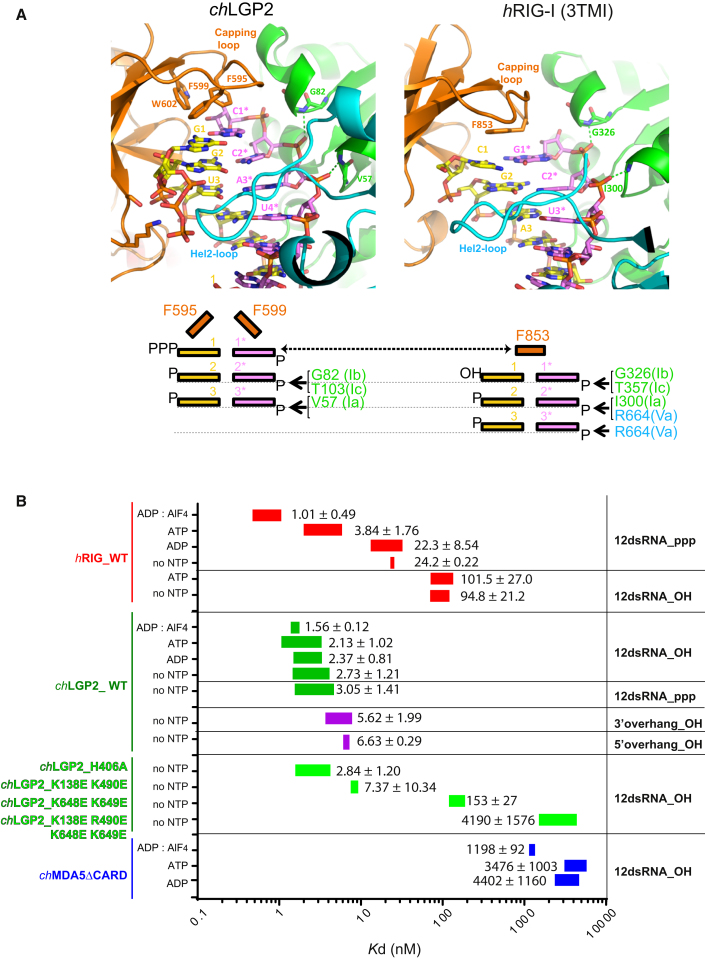
Comparison of *ch*LGP2 and *h*RIG-I Binding to dsRNA (A) Structural and schematic diagrams comparing the mode of end binding of *ch*LGP2 and *h*RIG-I illustrating the extra base pair sequestered by *ch*LGP2, which is at the same level as Phe853 from the capping loop of RIG-I. Conserved interactions with motifs Ia, Ib, and Ic are shown. (B) K_d_ values between *ch*LGP2 (green), full-length *h*RIG-I (red), and *ch*MDA5 (blue) and a 12-mer dsRNA with different 5′ modifications were measured without nucleotide (no NTP) and with various nucleotides (ADP-AlF_4_, ADP, ATP). The values shown correspond to K_d_ (nM) ± SD on the basis of the fluorescence anisotropy binding curves shown in Figure S5B. See also Figures S4 and S5.

Within the full-length *ch*LGP2 structure, the CTD interacts with the dsRNA extremity in a similar fashion to previously reported for the isolated *h*LGP2 CTD (PDB: 3EQT) (Li et al., 2009, Pippig et al., 2009), with the terminal base pair (5′-G1:C1^∗^-3′) making extensive hydrophobic interactions with aromatic residues Phe595, Phe599, and Trp602 from the 591–602 “capping loop” between strands β18 and β19 (Figure 2C). This loop also makes significant contacts with Hel1 in the vicinity of motif Ib, thereby reinforcing the closure of LGP2 over the RNA. The 3′ end interacts mainly with the turn between strands β16 and β17 (the 571–574 “3′ end-binding loop”; Figure S1), with fully conserved Glu571 hydrogen bonding with both hydroxyls of C1^∗^ and His574 bridging between the C1^∗^ and G2 riboses (Figure 2C). These interactions would appear to block extension of the 3′ strand of the dsRNA (but see below). The 5′ end interacts with both the CTD and the extended 402–413 “Hel2-loop” (Figure S1), which emerges between strand β10 and helix α16 and crosses the major groove of the dsRNA (Figure 2C). In *ch*LGP2, the G1 base is imperfectly stacked between His406 and tilted Phe595, whereas the equivalent residues in *h*LGP2 are aliphatic, Asn408 and Ile597, respectively. Interestingly, an aromatic residue (His or Tyr) is found at position 406 in the Hel2-loop only in bird, reptile, and amphibian LGP2s (i.e., not in mammals or fish, in which it is usually an Asn), whereas only birds (with a few exceptions) and frogs have an aromatic residue at position 595 (Figure S3). The deletion of the Hel2-loop significantly affects ATP hydrolysis by *h*MDA5 (Wu et al., 2013). We found that the H406A mutation in *ch*LGP2 also reduced ATP hydrolysis but did not significantly change the affinity to RNA (Figures 1E and 3B). The CTD of LGP2 contains a lysine-rich motif (644-KKKYKKWS-651), with highly conserved Lys648 and Lys649 contacting phosphates of both strands across the major groove (respectively A7^∗^, U8^∗^, and C5) and conserved Trp650 to U3 and A4 phosphates (Figure 2B). The K648/K649E double mutation reduces *ch*LGP2 affinity to dsRNA 56 times compared with wild-type (WT). A quadruple mutant with the additional glutamate substitutions of the conserved helicase residues Lys138 and Arg490 that interact with consecutive 5′ strand phosphates (Figure 2B) has ∼1,500 times less affinity, while the helicase mutations alone reduce the affinity by only 2.7 times (Figure 3B). This confirms the strong direct binding of the CTD to the RNA binding compared to the helicase domain and reveals the cooperativity of dsRNA binding to the two domains.

### *ch*LGP2 Binds One Extra Base Pair of dsRNA Compared with RIG-I

An intriguing observation is that the blunt end of the dsRNA is located 1 bp deeper into the *ch*LGP2 CTD than occurs in RIG-I (Figure 3A). This is not because the entire CTD is raised up but because the capping loop of LGP2 is flat, whereas that of RIG-I bends downward placing the side chain of Phe853 at the same level as the blunt-end base pair in LGP2 (Figure 3A). This shifts all the canonical contacts between helicase motifs and backbone phosphates 1 bp down from the blunt end in LGP2 compared with RIG-I, with motifs Ib and Ic contacting phosphate 1 and phosphate 2 of the 3′ strand in RIG-I and LGP2, respectively (Figure 3A). Consequently, the overall footprint of LGP2 on dsRNA is 10 bp for LGP2 rather than 9 bp for RIG-I, consistent with the larger surface area of protein-RNA contact for LGP2.

### *ch*LGP2 Binds to dsRNA with Modified 5′ and 3′ Ends

To further characterize RNA binding to *ch*LGP2, we determined structures with the 5′ppp form the same 10-mer dsRNA (i.e., the cognate RIG-I ligand) and a 5′ppp hairpin dsRNA with 3′ GG overhang at 2.2 Å (*ch*LGP2-10ppp) and 2.0 Å (*ch*LGP2-3′ovg) resolution, respectively (Table 1). Note that the hairpin stem has some base pair differences from the 10-mer dsRNA beyond the second base pair (Figure S4). We also measured the binding affinity of *ch*LGP2 to different RNAs with and without various nucleotides and compared the results with *h*RIG-I (Figure 3B).

In the *ch*LGP2-10ppp structure, the 5′ end has a unique conformation (corresponding to one of the *ch*LGP2-10p conformations), with the 5′ppp being accommodated without structural changes. The α- and β-phosphates interact with His406, Ser407, and Asn408 from the Hel2-loop, and the β- and γ-phosphates interact with Lys634 (Figure S4A). An octahedrally coordinated magnesium ion directly interacts with the α- and β-phosphates as well as the phosphate of G2 (Figure 2C). The 5′ppp conformation in the *ch*LGP2-3′ovg structure is different and resembles the other 5′ end conformation observed in the *ch*LGP2-10p structure. The tri-phosphate makes alternative interactions with Asn408, Arg607, and Lys634, and there is no bound magnesium (Figure S4B).

The *ch*LGP2-3ovg structure is overall similar to the previous structures, the major difference being a shift of the first G1-C1^∗^ base pair toward Hel1, a slight shift in the opposite direction of the CTD (including the capping and 3′ end-binding loops), and a significant rearrangement of the β3-α4 loop (residues 79–90), which would otherwise clash with the additional bases (Figure 2D). The net result is to disengage Glu571 and His574 from the ribose of C1 allowing 3′ extension of the RNA by extrusion of overhang nucleotide G-1^∗^ between the capping loop and the refolded β3-α4 loop. The G-1^∗^ base is stacked between the side chains of Arg600 and Lys87, and the resulting conformation of the β3-α4 loop permits G-1^∗^ and G-2^∗^ to make numerous interactions with residues 79–90, including a number of base-specific interactions (Figures 2E and 2F). This together with the burial of the G-2^∗^ ribose, whose 3′ OH makes a hydrogen bond to the phosphate of C1^∗^, suggests that the observed structure may be specific to this particular 3′-GG overhang but does show how relatively minor structural perturbations allow extrusion of a 3′ overhang, while extension of the double helical stem is prevented by maintenance of the stacking of the capping loop over the first base pair.

The affinities of LGP2 to various dsRNA ligands were determined by fluorescence anisotropy (Figure 3B). *ch*LGP2 binds to a blunt-end 12-mer dsRNA with an affinity independent of whether the 5′ is a hydroxyl or a tri-phosphate (Figures 3B and S5B), consistent with the lack of significant additional interactions to the tri-phosphate (and its variable conformation) observed in the crystal structures. By comparison, *h*RIG-I, as expected, binds to 5′ppp more tightly than to 5′-OH dsRNA, a result of the interactions of the 5′ppp with three lysines and a histidine, all strictly conserved (Luo et al., 2012). In addition, while *h*RIG-I’s affinity to 5′ppp dsRNA depends on which nucleotide is also bound, being highest for ADP:AlF_4_ (K_d_ ∼ 1 nM) and 20 times weaker with ADP or no nucleotide (Figure 3B), the affinity of *ch*LGP2 for dsRNA is independent of bound nucleotide (Figure 3B). Finally, consistent with the 3′-ovg structure, the affinity of *ch*LGP2 for 3′ and 5′ overhangs is only marginally reduced by a factor of two. In comparison, *h*RIG-I can also bind 3′ overhangs with high affinity (provided there is a 5′ppp), but 5′ overhangs are far less well accepted (Ramanathan et al., 2016).

### Overall Structure of *ch*MDA5

**Figure 4:**
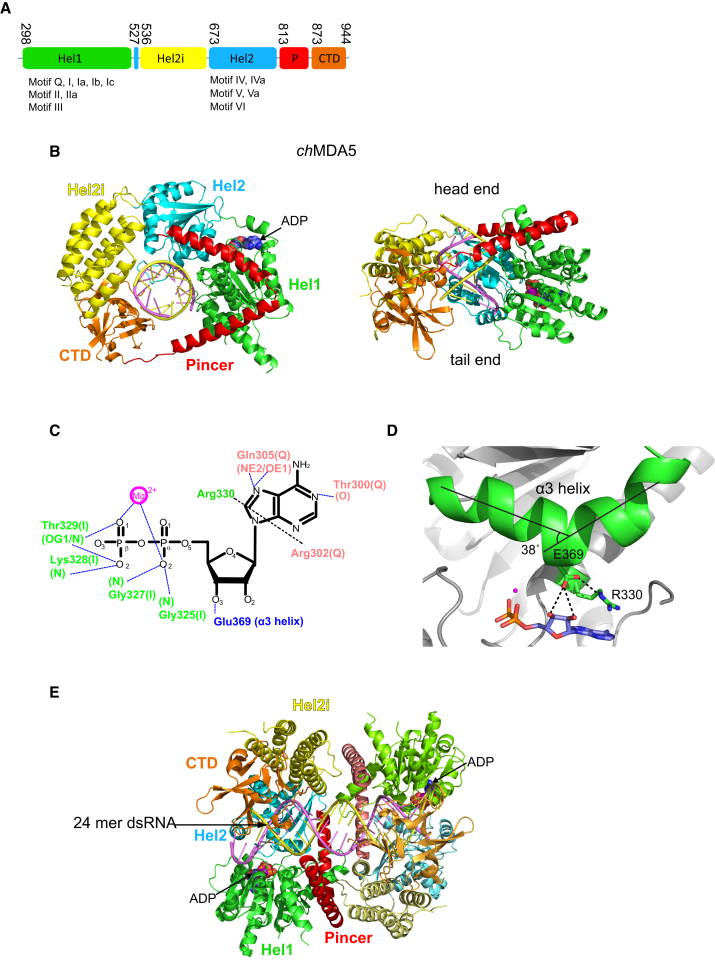
Overall Structure of *ch*MDA5ΔCARD-ADP Complex (A) Domain structure of *ch*MDA5ΔCARD, omitting the N-terminal tandem CARD domains. (B) Ribbon diagram of the *ch*MDA5ΔCARD-10-mer dsRNA-ADP complex from head end along (left) and perpendicular (right) to the dsRNA axis, colored according to Figure 2A. (C) Diagram showing the interactions of *ch*MDA5 with ADP:Mg. (D) Diagram showing how Glu369 emerges from helix *a*3 to be involved in ribose binding and stabilization of Arg330, which stacks on the adenine base. A very similar situation occurs in LGP2 but not RIG-I (see Figure 1D). (E) Ribbon diagram of the head-to-head *ch*MDA5ΔCARD-24-mer dsRNA-ADP complex perpendicular to the dsRNA axis. Domains and dsRNA are colored according to Figure 2A, with lighter colors for the second monomer.

In the absence of endogenous RIG-I, it is thought that *ch*MDA5, perhaps in co-operation with *ch*LGP2, is responsible for the RNA dependent anti-viral response to viruses that are usually detected by RIG-I (Liniger et al., 2012). We therefore determined the crystal structure of *ch*MDA5 (see Figure 4A for the domain structure) and investigated its ability, as well as that of *ch*LGP2, to form filaments with dsRNA.

For co-crystallization, we used 5′p palindromic duplex RNAs, the ATP analog AMPPNP, and a construct denoted *ch*MDA5ΔCARD-Q, comprising the helicase and CTD (residues 298–994) with the mutation E436Q and lacking seven residues at the C terminus. The E436Q mutant in conserved motif II (i.e., DECH becomes DQCH) virtually abolishes ATPase activity (Louber et al., 2015) and for another DEAD-box helicase, VASA, permitted trapping of the helicase substrate RNA (Xiol et al., 2014). Unlike for the *h*MDA5 crystal structure (Wu et al., 2013) the long, acidic loop between helixes α12 and α13 of Hel2i, which is 17 residues shorter in *ch*MDA5, was not deleted (Figure S1). The structure of a 1:1 complex of *ch*MDA5ΔCARD-Q with ADP:Mg^2+^ and 10 bp dsRNA (*ch*MDA5-10) and a 2:1 complex with 24 bp dsRNA (*ch*MDA5-24) were solved by molecular replacement at 2.60 and 2.75 Å, respectively (Table 2), as well as a 2:1 complex with 27 bp dsRNA at 7.2 Å resolution.

Overall, the *ch*MDA5-dsRNA structures resemble that of *h*MDA5 bound to a 12-mer dsRNA (PDB: 4GL2; root-mean-square deviation of 1.16 Å for all Cαs) (Figure 4) but with considerably higher resolution (*h*MDA5 is at 3.56 Å), and the model is more complete. Interestingly, the *h*MDA5, *ch*MDA5-10 (Figure 4B), *ch*MDA5-24 (Figure 4E), and *ch*MDA5-27 structures all capture the dsRNAs bound in different longitudinal positions. The *ch*MDA5-24 and *ch*MDA5-27 structures reveal two different 2:1 complexes with two MDA5 molecules bound to the same dsRNA. Following Wu et al. (2013), looking perpendicular to the RNA axis, we denote the head end of MDA5 as the pincer domain containing face and the tail end as that containing the CTD (see Figures 4B and S1 for the secondary structure of *ch*MDA5 and sequence alignments).

### *ch*MDA5 Bound to dsRNA and ADP:Mg^2+^ Is in the Semi-closed Conformation

In all *ch*MDA5-dsRNA structures, the helicase is in the semi-closed conformation (Figures 4B and S6; Table S1), but unlike the semi-closed forms of RIG-I and LGP2, Hel2 is well ordered. In the two higher resolution structures, the electron density in the nucleotide binding site clearly corresponds to ADP:Mg^2+^, presumably because of slow AMPPNP hydrolysis. The ADP:Mg^2+^ is bound in canonical fashion by motifs Q and I, with the adenosine stacked between Arg330 (motif I) and Arg302 (motif Q) (Figure 4C). This arrangement is more similar to that in *ch*LGP2 than RIG-I, with helix α3 being kinked toward the nucleotide binding cleft in the same way, allowing Glu369, stabilized by Arg330, to hydrogen-bond to the 3′ OH of ADP (corresponding to Glu67 and Arg32 in *ch*LGP2; compare Figures 1D and 4D).

### *ch*MDA5 Interaction with dsRNA

**Figure 5:**
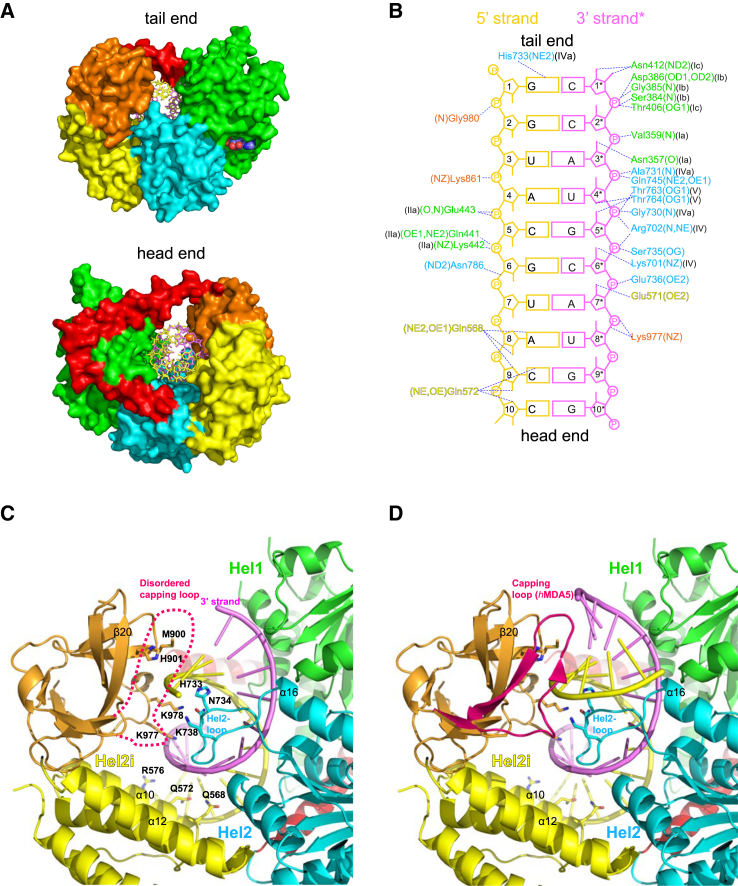
Interactions of *ch*MDA5 with dsRNA (A) Head and tail views down the dsRNA axis of the chMDA5 structure showing that the dsRNA can continue from both ends (see also D). Compared with LGP2 (Figure 2A), MDA5 appears from the tail end as an open horseshoe rather than a disk. (B) Schematic diagram showing the interactions of *ch*MDA5 residues with the 10-mer dsRNA, annotated as in Figure 2B. (C) Ribbon diagram showing protein-RNA interactions in the *ch*MDA5 10-mer structure at different levels along the dsRNA, including those from helix α12 of Hel2i, the Hel2 loop, and two loops of the CTD. The putative position of the disordered MDA5 capping loop is shown dotted on the basis of the crystal structure of the isolated *h*MDA5 CTD. Protein-RNA interactions mediated by the Hel1 and Hel2 domains have been omitted for clarity (see B). (D) As in (C), but the dsRNA has been extended by 3 bp to emerge from the tail end of the molecule. Modeling suggests that the MDA5 capping loop could be involved in another level of protein-RNA interactions as well as possibly mediating the tail-to-head protein-protein interface.

MDA5 binds to the dsRNA stem using the canonical helicase motifs Ia, Ib, Ic, IIa, IV, IVa, and V as well as conserved glutamines Gln568 and Gln572 from α10 of Hel2i, in a similar manner to other RLHs (Figure 5). The distinguishing feature of MDA5 (Wu et al., 2013) is that its CTD interacts intimately with Hel2i and is displaced relative to the position in LGP2 or RIG-I to allow a dsRNA helix to pass through the molecule. Thus, the domain arrangement in MDA5, at the tail end, resembles an open horseshoe rather than a closed circle (compare Figures 5A and 2A). The Hel2-loop (729–740 in *ch*MDA5, notably residues His733, Asn734, and Lys738; Figure S1), senses the major groove of the dsRNA, as in LGP2 and RIG-I, but without making any specific interactions (Figure 5C). The CTD 3′ end-binding loop (898-ENMH-901 in *ch*MDA5) contacts the backbone of the 5′ strand, without blocking extension of the 3′ end, and the conserved 977–980 turn, toward the end of the CTD, also makes backbone interactions to both strands in the major groove (Figure 5C). Another major distinctive feature of MDA5 is the position and nature of the CTD “capping loop” (918–927 in *ch*MDA5; Figure S1). While in all MDA5 crystal structures with dsRNA this loop is disordered, it is well structured in the isolated *h*MDA5 CTD structure (PDB: 3GA3), revealing it to be more compact than in LGP2 or RIG-I. Moreover, the MDA5 loop, unlike those of LGP2 and RIG-I, lacks the bulky hydrophobic residues that would favor interaction with a blunt end base pair. Modeling of the loop from the superposed *h*MDA5 CTD structure suggests that when binding to a continuous dsRNA the capping loop and N-terminal end of α16 of Hel2 could favorably interact with the minor groove and extended 5′ strand backbone, respectively (Figure 5D).

### Dimers of *ch*MDA5 on dsRNA Have the Head-Head Configuration

**Figure 6:**
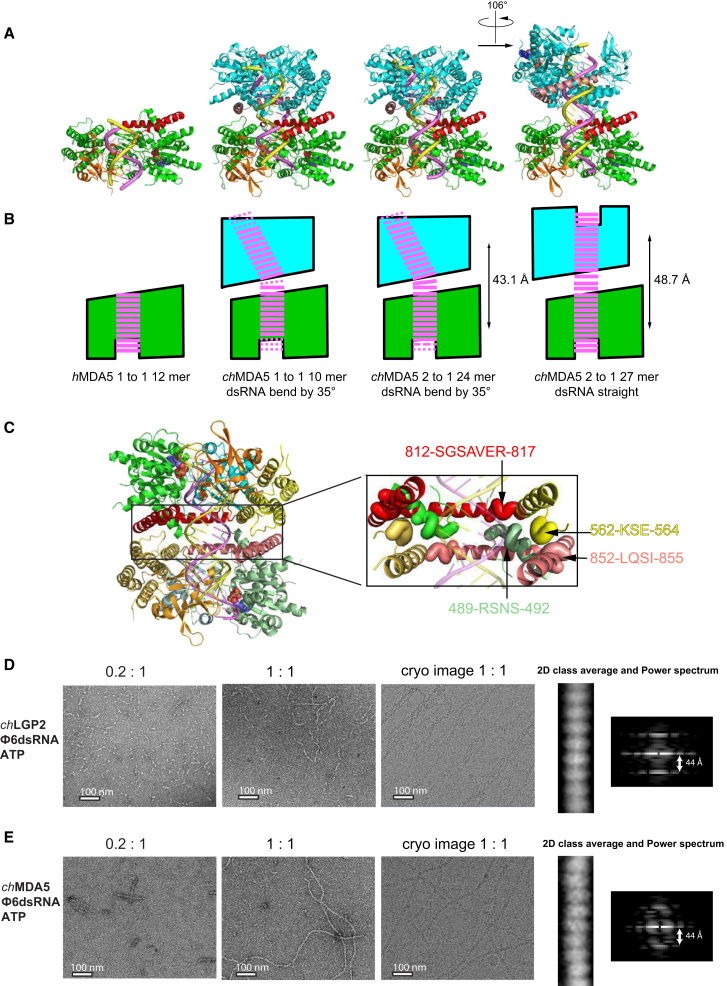
Dimer and Filament Formation by *ch*MDA5 and *ch*LGP2 (A) Comparison of monomeric and dimeric structures of MDA5-dsRNA complexes. *h*MDA5 12-mer (PDB: 4GL2) (left), *ch*MDA5 10-mer (middle-left), *ch*MDA5 24-mer (middle-right), and *ch*MDA5 27-mer (right). In all cases, the bottom molecule (green ribbons with CTD in orange, pincer domain in red) is in the same orientation. The top molecule is cyan except for the pincer domain (pink). The *ch*MDA5 10-mer and 24-mer structures are essentially the same apart from the lack of continuity of the dsRNA in the former. In both cases, the axis of the dsRNA is bent by 35° between the two head-to-head molecules, but in the *ch*MDA5 27-mer complex, it is straight. (B) Schematic representation of (A) highlighting the dsRNA conformation. The distance between the center of mass of the two molecules in the head-to-head dimer is indicated. Compared with the *h*MDA5 12-mer structure (left), the *ch*MDA5 10-mer structure (middle left) lacks 3 bp at the tail end and gains 1 bp at the head end, and there is a 1 bp gap between the RNAs in the two head-to-head molecules in the dimer (dotted base pairs indicate potential extra base pairs not in the structure). In the *ch*MDA5 24-mer (middle right), the dsRNA is continuous between the molecules in the dimer but bent. Because there is 1 bp between the two MDA5 molecules and 24 bp overall, the structure is likely a superposition of structures with either 11 or 12 bp bound to one MDA5 (i.e., 11.5 on average), as indicated by the dashed line. In the low-resolution *ch*MDA5 27-mer, it is unclear whether there are extra base pairs between the molecules, which are more widely separated, or at the tail ends, but the dsRNA is straight. (C) Detail of the four regions involved in protein-protein interactions mediating the head-to-head packing. (D) EM analysis of *ch*LGP2:Φ6 dsRNA complexes in the presence of ATP. Left: molar ratio 0.2:1 with uranyl acetate negative stain. Middle left: molar ratio 1:1 with negative stain. Middle right: molar ratio 1:1 with cryo-EM. Right: cryo-EM image 2D class average and power spectrum. (E) EM analysis of *ch*MDA5:Φ6 dsRNA complexes in the presence of ATP. Left: molar ratio 0.2:1 with negative stain. Middle left: molar ratio 1:1 with negative stain. Middle right: molar ratio 1:1 with cryo-EM. Right: cryo-EM image 2D class average and power spectrum.

In the *ch*MDA5 24-mer structure, two MDA5 molecules are stacked head to head on one dsRNA, with a two-fold symmetry axis perpendicular to the dsRNA and between the two molecules (Figures 4E and 6A); additionally there is a 35° bend in the dsRNA axis (Figures 6A and 6B). In the *ch*MDA5 10-mer structure, there are two 1:1 complexes in the asymmetric unit, which are arranged exactly as in the 24-mer structure except that the dsRNA is not continuous, lacking one base pair between the molecules (Figures 6A and 6B). The *h*MDA5 12-mer structure has two 1:1 complexes in the asymmetric unit, but with no pseudo-continuous RNA helix running through multiple MDA5 molecules. Compared with the *ch*MDA5 10-mer structure, the *h*MDA5 12-mer structure has three extra base pairs at the tail end but one fewer at the head end (Figures 6A and 6B). By comparison, the *ch*MDA5 24-mer structure gains 1 bp at the head end (making the dsRNA continuous between the two molecules) and 1–2 bp at the tail end (because the 24-mer structure probably corresponds to a superposition of two structures with a shift of 1 bp). More base pairs at the tail end correlate with a slight movement of the CTD toward the dsRNA. The bend in the dsRNA axis in the 1:1 and 2:1 *ch*MDA5 structures allows two MDA5 molecules to pack closely together in head-to-head fashion, with a center-of-mass to center-of-mass distance of 43.1 Å and total buried protein-protein surface of 1,961 and 1,907 Å^2^, respectively. The head-to-head interface involves two-fold symmetric interactions between the pincer domain of one molecule and two protruding loops of the other molecule (Figure 6C). The beginning of the pincer domain (812-SGSAVER-817) interacts with the loop preceding Hel1 helix α9 (489-RSNS-492), which protrudes from the surface of the neighboring molecule. The end of the pincer domain (852-LQSI-855) interacts with the loop (562-KSE-564) between helices α11 and α12 of the second molecule. Note that residues V816 and E817 in *ch*MDA5 are equivalent to I841 and E842, implicated in the head-to-tail interaction in *h*MDA5 (Wu et al., 2013).

In the *ch*MDA 27-mer complex, the dsRNA is straight. Moreover, compared with the 24-mer complex, the two MDA5 head-to-head molecules are more distantly separated by 48.7 Å, and their relative rotation differs, with the second molecule of the 27-mer dimer being rotated a further 106° around and translated 10.6 Å further along the dsRNA (Figures 6A and 6B). Only regions 852–855 and 562–564 are close enough to make contact, thus reducing the total buried surface area to 484 Å^2^. Thus, whereas in the case of the 24-mer 2:1 complex, stronger protein-protein interactions are able to distort the path of the dsRNA, the 27-mer structure paradoxically suggests that *ch*MDA5 prefers to bind at the end of a short dsRNA, even if this means reducing protein-protein interactions. However, it cannot be ruled out that the observed arrangement is dependent on crystal contacts.

### *ch*LGP2 Makes Filaments with dsRNA

To test the ability of *ch*LGP2 to form filaments on continuous dsRNA, complexes with Φ6 2,948, 4,063, and 6,374 bp long dsRNA were studied by EM. When mixed with a protein/RNA ratio of 0.2:1 in the presence of 2 mM ATP and imaged using negative stain, *ch*LGP2 forms short and discrete multimeric clusters on Φ6 dsRNA (Figure 6D). At a ratio of 1:1, corresponding to one protein per 15 bp, as in *h*MDA5 polar filaments (Berke et al., 2012, Wu et al., 2013), more extensive and regular dsRNA coating interspersed with naked dsRNA is observed (Figure 6D). The presence of ATP or ADP:AlF_4_ favors the formation of filamentous regions. In comparison, at a 1:1 ratio, full-length *ch*MDA5 completely coats dsRNA, independently of bound nucleotide, and at a ratio of 0.2:1, long clusters are observed on a few dsRNA molecules (Figure 6E). Thus, *ch*LGP2 binding to dsRNA is less co-operative than *ch*MDA5 (this work) and *h*MDA5 (Berke et al., 2012, Peisley et al., 2011, Wu et al., 2013). Imaging of *ch*LGP2 and full-length *ch*MDA5 filaments by cryo-EM shows that they are qualitatively similar, with a subunit axial translation of ∼44 Å (Figures 6D and 6E), identical to that reported for *h*MDA5 (Berke et al., 2012).

### Functional Studies of LGP2/MDA5 Cooperativity

**Figure 7:**
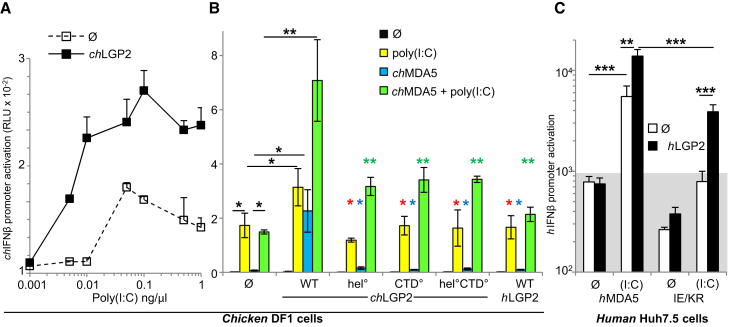
Cooperativity of LGP2 with MDA5 in Cells (A) Enhancement of poly(I:C)-mediated activation of endogenous *ch*MDA5 by exogenously supplied *ch*LGP2 (27.5 ng DNA/well) in chicken DF1 cells. See also Figures S7A–S7C. (B) Effect of *ch*LGP2, *ch*LGP2 variants (K138E/R490E or hel°, K648E/K649E or CTD°, K138E/R490E/K648E/K649E or hel°CTD°), and *h*LGP2 (27.5 ng DNA/well) on the activation of *ch*IFNβ promoter by endogenous *ch*MDA5 stimulated (yellow bars) or not (blue bars) by poly(I:C) and by exogenous *ch*MDA5 (1.67 ng DNA /well) in the absence (black bars) and presence (green bars) of poly(I:C) in chicken DF1 cells. ^∗^p < 0.05 and ^∗∗^p > 0.025 and below. Comparison of endogenous MDA5 and exogenous MDA5 co-transfected or not with LGP2 and activated or not with poly(I:C) are in black, LGP2 activated *ch*LGP2 variants with WT counterpart are in red (effect on endogenous *ch*MDA5), blue (effect on exogenous *ch*MDA5), and green (effect on exogenous *ch*MDA5 + poly[I:C]). Data are mean ± SD of three independent experiments, with each combination done in triplicate each time. See also Figures S7D–S7F. (C) Effect of hLGP2 on the activation of exogenous *h*MDA5 and *h*MDA5 IE/KR (1.67 ng DNA/well) (I841K/E842R) mutant by poly(I:C) (denoted [I:C]) in the absence (white bars) and presence (black bars) of exogenously supplied *h*LGP2 (27.5 ng DNA/well) in human Huh7.5 cells. ^∗∗^p < 0.01 and ^∗∗∗^p > 0.0025. Background signal (mean value) of cells transfected with control plasmid DNA followed or not by transfection of poly(I:C) is indicated by the shaded area. See also Figure S7G. See also Figure S7.

The mechanism of *ch*LGP2 stimulation of *ch*MDA5 function was investigated using the RNA binding-deficient *ch*LGP2 mutants in the CTD, helicase, or both, which we characterized above and monitoring IFN-β activation in chicken DF1 cells upon stimulation with poly(I:C). Exogenously provided *ch*LGP2 readily augments the response of endogenous *ch*MDA5 to poly(I:C) (Figures 7A and S7A), as also found in the human system (Figures S7B and S7C), whereas LGP2 itself lacks transduction ability (Figures 7B and S7D) (Rothenfusser et al., 2005). Unlike the human system, in which exogenous *h*MDA5 exhibits a constitutive activity not enhanced by exogenous *h*LGP2 (Figure S7D, no RNA), exogenous *ch*MDA5 lacks constitutive activity, but co-transfection with *ch*LGP2 results in significant activation of the *ch*IFNβ promoter (Figure 7B, compare WT and Ø, blue bars) suggesting that *ch*LGP2 overexpression can stabilize the binding of *ch*MDA5 to cellular RNAs (as found with ATP hydrolysis mutants of MDA5; Louber et al., 2015). Co-transfection of *ch*LGP2 has a strong enhancing effect on the activation of limiting amounts of exogenous *ch*MDA5 by poly(I:C) (Figure 7B, compare green and blue bars for wild-type *ch*LGP2), similar to the dose-dependent enhancement observed in the human system (Figures S7D and S7E) (Bruns et al., 2013, Bruns et al., 2014, Pippig et al., 2009). Enhancing activities were most evident when either MDA5 or poly(I:C) was in limited amounts. Furthermore there appears to be a species restriction, as *h*LGP2 readily enhances *h*MDA5 activation in human Huh7.5 cells (Figures S7C–S7E) but is inactive toward *ch*MDA5 in DF1 cells (Figure 7B). RNA binding-deficient *ch*LGP2 mutants in the CTD, helicase, or both show poor enhancing activity toward *ch*MDA5 (Figure 7B, CTD°, hel°, and CTD°-hel° constructs), also observed in the human system (Figure S7F). Finally, *h*LGP2 also exhibited a potent enhancing effect on the poly(I:C)-mediated activation of the *h*MDA5 IE/KR mutant, which has been reported to be deficient in signaling because of a defect in homopolymerization (Louber et al., 2015, Wu et al., 2013) (Figure 7C), this property being abrogated with RNA binding-deficient *h*LGP2 mutants (Figure S7G).

## Discussion

We describe unusually high-resolution crystal structures of *ch*LGP2 bound to dsRNA and ADP:AlF_4_, which show *ch*LGP2 to be in the fully closed, ATP hydrolysis transition state. *ch*LGP2 binds to dsRNA with its CTD capping the blunt end, similarly to RIG-I, but differs in having a longer overall footprint and by accommodating 5′ or 3′ overhangs with relatively minor perturbations in structure and little loss in affinity.

We also present several structures of *ch*MDA5, all of which are in the semi-closed state with ADP:Mg^2+^ bound. *ch*MDA5 seems to prefer head-to-head packing when bound to short dsRNA (24-mer to 27-mer) in contrast to the polar head-to-tail packing described for long, signaling active *h*MDA5-dsRNA filaments (Berke et al., 2012, Wu et al., 2013). *ch*LGP2 also forms filaments on dsRNA structurally resembling those of MDA5, although *ch*LGP2 dsRNA coating is less co-operative.

Functionally, conserved RNA binding determinants in both the CTD and helicase are required for LGP2-mediated enhancement of MDA5 signaling in both chicken and human, suggesting a conserved underlying mechanism for this phenomenon.

### Open and Closed Helicase Conformations of RLHs

Structures of RIG-I and MDA5 can be classified into four states depending on the degree of closure of the Hel1 and Hel2 domains, and this is usually correlated to the type of bound nucleotide (Table S1; Figure S6A). RNA free structures of full-length RIG-I (in which the CARDs are bound to Hel2i) or the helicase domain alone are in the very flexible “open” conformation with Hel1 and Hel2 well separated and Hel2 poorly ordered (Kowalinski et al., 2011, Civril et al., 2011, Deimling et al., 2014). Most *h*RIG-I helicase-CTD-dsRNA structures with no nucleotide, sulfate, or ADP are in the “semi-open” conformation with the poorly ordered Hel2 not contacting the RNA (Kohlway et al., 2013, Luo et al., 2011, Luo et al., 2012). In one “semi-closed” state structure, co-crystallized with ATP analog ADP:BeF_3_, Hel2 is better ordered, but motifs V and VI do not engage with the nucleotide (Jiang et al., 2011). The only fully “closed” RIG-I structure in which all motifs are correctly positioned for ATP hydrolysis, is that of the isolated dRIG-I helicase domain bound to dsRNA and ADP:AlF4 (Kowalinski et al., 2011). The closed conformation of the *ch*LGP2-dsRNA-ADP:AlF4 complexes reported here thus closely resembles the latter *d*RIG-I structure. In contrast, the *ch*LGP2 12-mer dsRNA structure without nucleotide is semi-closed with Hel2 poorly ordered, except where it contacts the RNA. Intriguingly, a closed structure of RIG-I including the CTD and an ATP analog has not yet been reported.

To characterize quantitatively these different conformations, we superposed, via the Hel1 domain, all RLH structures on the *ch*LGP2 closed state as reference. The rotation angle required to superpose the Hel2 domain of the test structure on that of the reference structure varies systematically in concordance with the qualitative description above, being 0° to 3° for the closed state, 7° to 13° for the semi-closed state, ∼40° for the semi-open state, and 50° to 60° for the open state (Table S1).

The two different *ch*LGP2 conformations described here provide a unique opportunity to compare the closed and semi-closed states of the same RLH. The ∼10° rotation of Hel2 between the closed state and semi-closed states corresponds to a shift of the backbone interactions made by Hel2 (mediated by motifs IVa and V) by one phosphate down the 3′ strand of the dsRNA, whereas the interactions of Hel1 (mediated by motifs Ia, Ib, Ic, and IIa) with the dsRNA are unchanged (Figures S6B and S6C). The movement of Hel2 is coupled to that of Hel2i, which pivots around its contact with the CTD (which itself does not shift much), while maintaining similar contacts with the RNA (Figure S6B). This comparison highlights an ATP-dependent structural transition, which in one sense can correspond to a co-operative tightening of dsRNA interactions upon ATP binding and in the other sense the relaxation of the grip upon the dsRNA upon ATP hydrolysis. This structural transition needs to be taken into account in understanding the role of ATP binding and hydrolysis in RLH function, including filament formation.

### End Binder and/or Stem Binder?

The stem binder MDA5 and end-capping LGP2/RIG-I critically differ in the position of the CTD and the nature of the residues on the CTD “capping loop” (Figure S1). In LGP2 and RIG-I “capping loop” aromatic hydrophobic amino acids (Phe595, Phe599, and Trp602 in *ch*LGP2; Phe853 and Phe856 in *h*RIG-I) contact the blunt-end base pair of the dsRNA by edge-on or stacking interactions, essentially preventing binding to a continuous stem. In MDA5, the loop contains no bulky hydrophobic residues and is disordered in all structures of MDA5 bound to dsRNA so far. However, it is ordered in the structure of the *h*MDA5 CTD alone (PDB: 3GA3), where it forms an additional β strand to the main β sheet of the CTD rather than bulging out as in RIG-I and LGP2 (Figure 5D). As pointed out previously (Wu et al., 2013), MDA5 CTD is significantly displaced away from the dsRNA compared with RIG-I and is able to pack closer to Hel2i because of the 10 residues shorter α12 in MDA5 Hel2i. In *ch*MDA5, the pining of the CTD to Hel2i is reinforced by the wrapping of the extended loop between α14 and α15 of Hel2i (residues 629–646) over the CTD (e.g., Asp635 interacts with His914 and Arg916 of CTD domain). In *ch*MDA5, this α14-α15 loop is shorter than that of *h*MDA5 (638–670) but longer than in RIG-I or LGP2. The close CTD-Hel2i interaction combined with the less hydrophobic and less intrusive CTD capping loop allows MDA5 to bind internally to dsRNA.

Interestingly, *ch*LGP2 resembles a chimera, combining a MDA5-like helicase domain and a RIG-I like CTD. The similarity of LGP2 to MDA5 is reflected in common structural features, not found in RIG-I, such as the mode of nucleotide binding (Figures 1D and 4D) and the length of the Hel2i domain helices (Figure S1) and susceptibility to protein V. However, the hydrophobic nature of the LGP2 CTD capping loop is clearly RIG-I like. Corresponding to these mixed structural features, LGP2 has the strong blunt-end binding characteristic of RIG-I yet is also able to make MDA5-like filaments, albeit less efficiently. Perhaps the chimeric nature of the LGP2 structure allows its CTD to adopt a different position closer to Hel2i, allowing LGP2 to form filaments similarly to MDA5, whereas RIG-I does not form well-ordered, extended filaments. The similarity in overall appearance and axial subunit translation of LGP2 filaments to MDA5 filaments suggests that LGP2 could also form head-to-tail packing on long dsRNA. Furthermore, modeling suggests that in MDA5, the capping loop could not only play a role in dsRNA binding (Figure 5D) but also be involved in the tail-to-head protein-protein interface. The same could be true in the case of LGP2, although it is unclear what alternative interactions the aromatic residues on the capping loop of LGP2 might make. This and related questions will be answered only by high-resolution EM reconstructions of LGP2 and MDA5 filaments.

### Head-to-head and Head-to-Tail Packing of MDA5

The head-to-head arrangement we observe for dimers of MDA5 bound to short dsRNA contrasts with the intermolecular packing arrangement derived from EM reconstructions of mouse MDA5-coated dsRNA filaments, which exhibit a polar head-to-tail packing (Berke et al., 2012, Wu et al., 2013). When the *ch*MDA5 structure was fitted into the EM map of the helical filament, the superposition also showed that the head-to-tail configuration fits significantly better (correlation coefficient 0.87 compared with 0.78 for head to head). However, the *ch*MDA5-24 structure indicates that on short dsRNA, and in the absence of stabilizing co-operative interactions forming an extended multimer, head-to-head stacking appears to be the preferred mode of interaction, and head-to-tail packing has not yet been observed in any crystal form. Thus it is possible that a head-to-head dimer nucleates filament formation. Interestingly, Dicer-related helicase 3, an ortholog of the Dicer and RIG-I that is essential for secondary small interfering RNA production in *Caenorhabditis elegans*, binds and recognizes 22G-RNA as a dimer, which has been modeled as a head-to-head dimer (Fitzgerald et al., 2014).

### Biological Role of LGP2 as a Regulator of RLH Signaling

Various hypotheses exist as to how LGP2 might play positive and negative roles in RLH signaling (Rodriguez et al., 2014). The high affinity of LGP2 end binding to short dsRNA supports that it could potentially compete for cognate RIG-I ligands and thus exert a negative effect, as proposed by others (Pippig et al., 2009, Rothenfusser et al., 2005). However, the affinity for 5′ppp-dsRNA is slightly lower than for RIG-I, suggesting that an excess of LGP2 over RIG-I might be required, as observed by others (Childs et al., 2013). Such a high unbalance between the two proteins, both of which are similarly transcriptionally upregulated by type I IFN (Rothenfusser et al., 2005), remains to be demonstrated in natural infection conditions. LGP2 has a considerably higher affinity for non-phosphorylated blunt-end dsRNA than RIG-I and thus could act as sponge to prevent misactivation of RIG-I by such RNAs, which may exist in the host cell. This would be an additional mechanism to promote self-discrimination or non-self-discrimination by RIG-I in parallel with the recently recognized role of the RIG-I ATPase activity and the CARD2-Hel2i interface to prevent deleterious constitutive activation of RIG-I by more weakly binding, non-cognate cellular RNAs (Anchisi et al., 2015, Lässig et al., 2015, Louber et al., 2015, Ramanathan et al., 2016, Rawling et al., 2015).

The mechanism of enhancement of MDA5 signaling by LGP2 in human cells has been proposed to depend on a direct effect of LGP2 on MDA5 filament formation (Bruns et al., 2014, Childs et al., 2013). It was concluded that LGP2 attenuates MDA5 filament length, giving rise to shorter complexes that more efficiently stimulate anti-viral signaling than longer MDA5 filaments (Bruns et al., 2014). In the chicken system, LGP2-stimulated MDA5 signaling could work by a similar mechanism. Additionally, our results suggest that LGP2 could potentially compensate for the lack of RIG-I through high-affinity end binding to a RIG-I like ligand, which could then nucleate, by RNA-dependent protein-protein interactions, signaling-competent MDA5 oligomer formation. Such a mechanism is supported by the observed strong RNA end binding mode of LGP2 (which is more permissive in terms of 3′ and 5′ modifications than RIG-I) and the functional requirement of an intact RNA binding site on both the CTD and helicase. Interestingly, the enhancing effect is observed only when RNA ligand and/or MDA5 are in limited amounts, as is likely to be the case early in infection.

In conclusion, our structural and functional results lay the basis for further studies to elucidate the exact mechanistic role of LGP2 in regulating MDA5 signaling by, for instance, determining the composition and structure of mixed filaments and/or oligomers containing both MDA5 and LGP2.

## Experimental Procedures

### Protein Preparation and Crystallization

Purified c*h*MDA5 (full-length, residues 1–1,001), *ch*MDA5ΔCARD (residues 298–994), *ch*MDA5ΔCARD-E436Q constructs, and a SUMO fusion of full-length *ch*LGP2 (1–674), expressed in *Escherichia coli*, were mixed with dsRNA of various lengths, prepared by in vitro T7 transcription, and either AMPPNP or ADP:AlF_4_ and initially screened for crystallization using a Cartesian robot.

### Crystallography

Optimized crystals were flash frozen and diffraction data collected on various beamlines at the European Synchrotron Radiation Facility (ESRF). Data were processed with XDS (Kabsch, 2010) and further analyzed using the CCP4 suite (Winn et al., 2011). Structures were determined by molecular replacement and refined with REFMAC5 (Murshudov et al., 1997).

### EM

Full-length *ch*MDA5 or *ch*LGP2 complexes with Φ6 bacteriophage dsRNA (Thermo Scientific), with or without ATP analogs, were examined by negative stain EM. For cryo-EM, 1:1 mixtures (one MDA5/LGP2 molecule per 15 bp of dsRNA) were frozen on glow-discharged grids (Quantifoil Micro Tools) and data collected with an FEI Polara microscope equipped with a K2 summit detector (Gatan). Power spectra were calculated from masked 2D class averages.

### Fluorescence Polarization Anisotropy

*ch*MDA5, *ch*LGP2, or *h*RIG-I was titrated into dsRNA solutions made by annealing 5′-FAM-labeled 12-mer RNA with unlabeled cRNA. Anisotropic measurements were made with excitation wavelength 495 nm and emission wavelength 515 nm during 100 s and K_d_ values derived by curve fitting.

### ATPase Activity Assays

dsRNA-dependent ATPase reactions were performed using a Malachite green assay kit (Bioassays) over 0.5–30 min, as detailed previously (Louber et al., 2015).

### Cellular Assays

IFN-β promoter activation upon transient expression of expression vectors coding for chicken or human MDA5 and LGP2 and poly(I:C) stimulation in chicken DF1 and human Huh7.5 cells was determined as previously described (Louber et al., 2015)

## Author Contributions

E.U. designed experiments and performed biochemistry, crystallization, data collection, and structural analysis. M.L. helped with protein production. H.M. with the help of E.U. performed the EM analysis. J.B. and D.G. designed, performed, and/or analyzed cellular experiments. S.C. directed the project, performed structural analysis, and wrote the paper with input from the other authors.

## Figures and Tables

**Table 1:** Diffraction Data and Refinement Statistics for *ch*LGP2

Crystal	*ch*LGP2 5′ppp 10-mer dsRNA ADP:AlF_4_:Mg^2+^	*ch*LGP2 5′p 10-mer dsRNA ADP:AlF_4_:Mg^2+^	*ch*LGP2 5′ppp 10-mer 3′ ovg dsRNA ADP:AlF_4_:Mg^2+^	*ch*LGP2 5′p 12-mer dsRNA
**Diffraction data**				

Space group	*P*2_1_2_1_2_1_	*P*2_1_2_1_2_1_	*P*2_1_2_1_2_1_	*P*6_4_
Cell dimensions (Å)	a = 70.14, b = 96.58	a = 69.59, b = 97.05	a = 69.86, b = 97.43	a = b = 90.09,
c = 122.86	c = 122.52	c = 122.58	c = 196.73
α = β = γ = 90	α = β = γ = 90	α = β = γ = 90	α = β = 90, γ = 120.00
Wavelength (Å)	0.8726	0.9786	1.072	0.9786
Resolution range of data (last shell) (Å)	50.0–2.2 (2.28–2.2)	50.0–1.50 (1.55–1.50)	50.0–2.0 (2.10–2.0)	45.04–3.60 (3.73–3.60)
Completeness (last shell) (%)	99.9 (99.4)	99.9 (99.8)	99.9 (98.7)	99.8 (99.0)
R-sym (last shell) (%)	16.9 (83.5)	4.8 (74.6)	25.9 (79.5)	6.7 (118.0)
I/σI (last shell)	8.73 (1.87)	17.6 (2.31)	6.31 (1.38)	20.74 (2.01)
Redundancy (last shell)	6.64 (6.50)	5.25 (5.27)	4.47 (4.35)	11.51 (10.32)

**Refinement**				

Reflections used in refinement work (free)	40,943 (2,114)	126,226 (6,681)	53,847 (2,814)	10,121 (542)
R-work (last shell)	0.188 (0.267)	0.133 (0.244)	0.218 (0.333)	0.253 (0.359)
R-free (last shell)	0.245 (0.294)	0.168 (0.292)	0.255 (0.317)	0.319 (0.346)
Number of non-hydrogen atoms	6,458	6,638	6,188	5,540
Protein	5,616	5,531	5,356	5,028
RNA	464	495	506	512
Ligand (nucleotide)	33	33	33	–
Solvent	345	579	293	–

**Geometry and B factors**				

Rms (bonds)	0.009	0.013	0.008	0.006
Rms (angles)	1.365	1.510	1.278	0.954
Ramachandran favored (%)	97.1	98.4	97.5	94
Ramachandran outliers (%)	0.72	0.15	0.15	0.32
Clash score	1.59	3.70	1.93	1.11
Average B factor	34.0	29.9	39.2	202.8
Protein	34.3	29.5	39.4	205.9
RNA	30.8	22.8	36.7	173.1
Ligand (nucleotide)	20.4	22.9	30.4	–
Solvent	33.6	40.7	38.7	–

Rms, root-mean-square.

**Table 2:** Diffraction Data and Refinement Statistics for *ch*MDA5

Crystal	chMDA5 5′p 10-mer dsRNA ADP-Mg^2+^ Twinned	chMDA5 5′p 10-mer dsRNA ADP-Mg^2+^	chMDA5 5′p 10-mer dsRNA ADP-Mg^2+^ Untwinned	chMDA5 5′p 24-mer dsRNA ADP-Mg^2+^
**Diffraction data**				

Space group	*P*2_1_	*P*2_1_2_1_2_1_	*P*2_1_	*P*2_1_2_1_2_1_
Cell dimensions (Å)	a = 70.16, b = 138.70, c = 100.42	a = 101.92, b = 132.47, c = 139.04	a = 72.08, b = 139.73, c = 103.19	a = 99.75, b = 133.40, c = 138.44
α = γ = 90, β = 109.48	α = β = γ = 90	α = γ = 90, β = 110.142	α = β = γ = 90
Wavelength (Å)	0.9724	0.9763	0.9763	0.9724
Resolution range of data (last shell) (Å)	50.0–2.60 (2.69–2.60)	50.0–2.60 (2.69–2.60)	48.6–2.95 (3.06–2.95)	50.0–2.75 (2.82–2.75)
Completeness (last shell) (%)	99.3 (99.6)	98.2 (99.2)	94.0 (96.6)	99.1 (99.2)
R-sym (last shell) (%)	9.60 (89.6)	9.60 (99.2)	9.4 (87.3)	16.8 (80.9)
I/σI (last shell)	9.83 (1.73)	10.31 (1.87)	9.94 (1.78)	6.58 (1.52)
Redundancy (last shell)	3.52 (3.63)	5.06 (5.21)	5.20 (5.05)	4.66 (4.89)

**Refinement**				

Reflections used in refinement work (free)	52,489 (2,811)	54,624 (2,901)	36,153 (1,907)	45,840 (2,433)
R-work (last shell)	0.271 (0.273)	0.277 (0.401)	0.256 (0.423)	0.290 (0.487)
R-free (last shell)	0.290 (0.286)	0.312 (0.418)	0.270 (0.421)	0.322 (0.492)
Number of non-hydrogen atoms	11,761	11,870	11,661	11,616
Protein	10,851	10,773	10,751	10,539
RNA	854	873	854	1,021
Ligand (nucleotide)	56	56	56	56
Solvent	–	168	–	–

**Geometry**				

Rms (bonds)	0.007	0.007	0.008	0.008
Rms (angles)	1.11	1.06	1.28	1.21
Ramachandran favored (%)	96.3	96.0	95.8	93.7
Ramachandran outliers (%)	0.31	0.31	0	0.4
Clash score	1.08	0.39	0.48	1.4
Average B factor	58.1	67.2	81.7	70.0
Protein	59.1	67.2	83.5	71.2
RNA	44.4	56.2	58.6	56.9
Ligand	59.5	65.1	96.1	74.0
Solvent	–	49.1	–	–

Rms, root-mean-square.

## Accession Numbers

The accession numbers for the structure factors and coordinates reported in this paper are as follows: *ch*LGP2, 5′p 10-mer dsRNA:ADP:AlF_4_:Mg^2+^, PDB: 5JAJ; *ch*LGP2, 5′ppp 10-mer dsRNA:ADP:AlF_4_:Mg^2+^, PDB: 5JB2; *ch*LGP2, 5′ppp 3ovg 26-mer hairpin RNA:ADP:AlF_4_:Mg^2+^, PDB: 5JBG; *ch*LGP2, 5′p 12-mer dsRNA, PDB: 5JBJ; *ch*MDA5 1:1 complex, 5′p 10-mer dsRNA:ADP:Mg^2+^, monoclinic form, PDB: 5JC3 (twinned), PDB: 5JCH (untwinned), orthorhombic form, PDB: 5JCF; *ch*MDA5 2:1 complex, 5′p 24-mer dsRNA:ADP:Mg^2+^, PDB: 5JC7.

## Supplemental Information

Document S1. Supplemental Experimental Procedures, Figures S1–S7, and Table S1

Document S2. Article plus Supplemental Information
