# Supplementary material for: Structural Analysis of dsRNA Binding to Anti-viral Pattern Recognition Receptors LGP2 and MDA5
Source: Mol Cell. 2016 May 19;62(4):586–602. doi: 10.1016/j.molcel.2016.04.021 (PMC4885022; doi:10.1016/j.molcel.2016.04.021)
Supplement: Document S1. Supplemental Experimental Procedures, Figures S1–S7, and Table S1 [file mmc1.pdf]

Molecular Cell, Volume *62*

Supplemental Information

Structural Analysis of dsRNA Binding to Anti-viral Pattern Recognition Receptors LGP2 and MDA5

Emiko Uchikawa, Mathilde Lethier, Hélène Malet, Joanna Brunel, Denis Gerlier, and Stephen Cusack

Structural analysis of dsRNA binding to

anti-viral pattern recognition receptors LGP2 and MDA5

Emiko Uchikawa[1,2], Mathilde Lethier[1,2], Hélène Malet[1,2],

Joanna Brunel[3-7], Denis Gerlier[3-7] and Stephen Cusack[1,2#]

[1]European Molecular Biology Laboratory, Grenoble Outstation, 71 Avenue des Martyrs, CS 90181, 38042 Grenoble Cedex 9, France

[2]University Grenoble Alpes-Centre National de la Recherche Scientifique-EMBL Unit of Virus Host-Cell Interactions, 71 Avenue des Martyrs, CS 90181, 38042 Grenoble Cedex 9, France.

[3]CIRI, International Center for Infectiology Research, Université de Lyon, Lyon, France;

[4]Inserm, U1111, Lyon, France;

[5]CNRS, UMR5308, Lyon, France;

[6]Ecole Normale Supérieure de Lyon, Lyon, France;

[7]Université Lyon 1, Centre International de Recherche en Infectiologie, Lyon, France

#Corresponding author:

Stephen Cusack

European Molecular Biology Laboratory, Grenoble Outstation, Grenoble Cedex 9, France.

Tel. (33)476207238, Email: cusack@embl.fr

Supplemental Information

- Supplementary Figure Legends S1-S7
- Supplementary Figures
- Supplementary Table S1
- Supplemental Experimental Procedures
- Supplemental References.

Supplementary Figure Legends.

Figure S1 Sequence alignment of chicken and human LGP2 and MDA5 and duck and human RIG-I (excluding the CARDs). Related to Figures 1 and 4.

The secondary structure of *ch*LGP2 and *ch*MDA5 are at the top and bottom respectively. Outline boxes in green, yellow, cyan, red and orange delimit the Hel1, Hel2i, Hel2, Pincer and CTD domains. Helicase conserved motifs are indicated in black on the bottom and important loops such as the Hel2i, Hel2, end-binding and capping loops are highlighted with a green, yellow, cyan, magenta or orange background respectively. Figure made with ESPript (Gouet et al., 1999).

Figure S2. ATP analogue binding and ATP hydrolysis by *ch*LGP2. Related to Figure 1.

(A) Diagram showing interaction network between *ch*LGP2 and ADP:AlF_4:Mg^{2+}. Residue colours are as in Figure1C with polar interactions (blue) and hydrophobic interactions (black).

(B) Ligand electron density for ADP:AlF_4:Mg^{2+} in the *ch*LGP2 5′p 10-mer dsRNA complex with Mg^{2+} (magenta), Al (grey), F (pale blue) and coordinated water (marine). Final 2Fo-Fc electron density is contoured at 2.0 σ.

(C) Comparative RNA-dependent ATP hydrolysis activity by LGP2, RIG-I and MDA5 showing initial reaction velocities versus ATP concentration. The concentration of proteins and dsRNAs are 0.125 μM *d*Rig-I and 0.5 μM dsRNA, 0.25 μM *ch*LGP2 and 1 μM dsRNA, 0.5 μM *ch*MDA5 and 2 μM dsRNA. Plotted values are mean ±SD (n=3).

(D) ATPase activity of RLHs as a function dsRNA length using 18-mer hairpin (5′-GGGCGGCUUCGGCCGCCC-3′), 24-mer (same dsRNA as for crystallization), 32-mer hairpin (5′-pppGGGCGAGCGUGCGCUUCGGCGCACGCUCGCCC-3′), or 38-mer (5′-GGGACGUAGCAUCCGAUGUACAUCGGAUGCUACGUCCC-3′). The graph compares the amount of hydrolysis after 16 minutes. The reaction conditions were 1 μM RLH, 4 μM dsRNA and 2 mM ATP. Poly(I:C) is polyinosinic-polycytidylic acid (Amersham Biosciences).

(E) Multiple sequence alignment of Motif I of RLHs

(F) Multiple sequence alignment of Motif VI of RLHs

Figure S3. Sequence alignment of LGP2 from representative vertebrates. Related to Figure 1.

Sequences aligned include those from birds (chicken, Muscovy duck), mammals (human, mouse), reptile (alligator), amphibians (frog) and fish (zebrafish). Annotation is similar to Figure S1.

Figure S4. 5′ tri-phosphate conformation and summary of RNAs used. Related to Figures 2, 3 and 5.

(A) Schematic diagram showing the interactions with the 5′ tri-phosphate in the *ch*LGP2_10ppp structure.

(B) Schematic diagram showing the interactions with the 5′ tri-phosphate in the *ch*LGP2_3′ovg structure.

(C) RNAs used in various experiments.

Figure S5. Comparative binding of RLHs to dsRNA. Related to Figure 2.

(A) Electron density showing residues from Hel2i helix α10 interacting directly or via water molecules with nucleotides from the 3′ (violet) and 5′ (yellow) strands in the *ch*LGP2 5′p 10-mer dsRNA complex. Final 2Fo-Fc electron density is contoured at 1.5 σ.

(B) Representative curves of fluorescence anisotropy changes measured by titrating full-length *h*RIG-I (upper panel), *ch*LGP2 (middle panel) or *ch*MDA5ΔCARD (bottom panel) to FAM labelled 5′OH, 5′ppp, 3′ overhang and 5′ overhang 12-mer dsRNA with or without various nucleotides.

Figure S6. Comparison of helicase opening in known RLH crystal structures and comparison of the closed and semi-closed states of *ch*LGP2. Related to all Figures.

(A) Disposition of helicase Hel1 (green) and Hel2 (cyan) domains for known RLH crystal structures (with PDB entry code indicated) after superposition of Hel1 of each structure onto that of the closed state of *ch*LGP2, used as reference. ATP analogues (slate blue) and Mg^{2+} (magenta) are shown with spheres and the dsRNA back bone (orange). Other domains are coloured as in Fig 1A. According to the relative displacement of Hel1 and Hel2, the structures are classified closed, semi-closed, semi-open and opened (see Table S1 for quantification).

(B) Comparison of the structures of the closed and semi-closed states of *ch*LGP2 after superposition of the Hel1 domain. The Hel1, Hel2, Hel2i and CTD domains are coloured green (pale green), cyan (pale cyan), yellow (sand) and orange (pale orange) for respectively and the dsRNA 5′ strand violet (pale violet) and 3′ strand yellow (sand) for the closed (semi-closed) states respectively.

(C) Schematic diagram comparing interactions made in the closed (left) and semi-closed (right) states of *ch*LGP2 to the dsRNA illustrating the shift of Hel2 interactions (red box) one phosphate down the 3′ strand.

Figure S7 . Co-operativity of LGP2 with MDA5 in human cells. Related to Figure 7.

(A) Expression of *ch*LGP2, *ch*LGP2 variants and *h*LGP2 in chicken DF1 cells (top) and expression of *h*LGP2 and *h*LGP2 variants in human Huh7.5 cells (bottom). The protein expression of other MDA5 constructs used in this work has been previously determined and found to be comparable to that of their *wt* counterpart (Louber et al., 2015).

(B) RIG-I and MDA5-mediated endogenous response to dsRNA in human HEK293 (in blue) and Huh7.5 (in red) cells. Activation of *hu*IFNβ promoter by RIG-I agonist 5′ppp-dsRNA (61mer), MDA5 agonist Φ6 bacteriophage 2.9, 4.1 and 6.4 kbp long dsRNA, and RIG-I/MDA5 agonist poly(I:C). Note the lack of detectable endogenous response observed in Huh7.5 cells and the neutral effect of the pCG-duF vector (noted "F") used as a complement loading DNA to ensure identical amounts of DNA in the experiments with expression vectors as a source of exogenous MDA5 and/or LGP2. (Related to Figure 7B)

(C) Enhancing effect of exogenously supplied *h*LGP2 on endogenous response to poly(I:C) in human HEK293 cells with the unit 1 corresponding to 5.5 ng LGP2 DNA/well. (Related to Figure 7A).

(D) Lack of signalling ability of exogenous *h*LGP2 upon activation with poly(I:C) or 5′ ppp-dsRNA (left) and dose-response enhancement by *h*LGP2 of the activation of exogenous *h*MDA5 by poly(I:C) (right) in human Huh7.5 cells. Note the high constitutive activation by MDA5_1 (corresponding to 5.5 ng DNA/well) (as

previously reported see (Louber et al., 2015) and references therein, see also panel E below, upper left histogram, condition 0 RNA) that is neither enhanced by exogenously added *h*LGP2 (compare histograms with no RNA (0) nor upon transfection of RIG-I agonist $^{5'ppp}$dsRNA with or without exogenous *h*LGP2 (compare histograms with 0 and $^{5'ppp}$dsRNA conditions). (Related to Figure 7B).

(E) Enhancing effect of *h*LGP2 on exogenous *h*MDA5 is observed only when transfected in limited amount and/or activated by a sub-optimal amount poly(I:C) in human Huh7.5 cells. Note the loss of dose-dependent enhancing effect of *h*LGP2 (with the unit 1 corresponding to 5.5 ng LGP2 DNA/well) in the presence of 0.05 ng versus 0.005 ng of poly(I:C) in the presence of 0.55, 1,67 and 5.5 ng/well of *h*MDA5 DNA (MDA5_0.1, MDA5_0.3, MDA5_1 at the upper right, bottom left and bottom right, respectively) and the reduction of this enhancing effect with increasing amount of *h*MDA5 (compare luciferase signal gradation according to *h*LGP2 amounts for a same amount of poly(I:C) in the presence of *h*MDA5 (MDA5_0.1, MDA5_0.3, MDA5_1)). Note also that the enhancing effect of *h*LGP2 on *h*MDA5 is restricted to poly(I:C) and is not observed with another MDA5 RNA agonist, the Φ6 bacteriophage 2.9, 4.1 and 6.4 kbp long dsRNA, while *h*MDA5 do respond also to this agonist in the absence of *hu*LGP2 (compare Φ6 and the no (0) conditions in the upper left panel for each amount of transfected *h*MDA5). (Related to Figure 7B).

(F) RNA binding deficient *h*LGP2 mutants (K138E/R492E or hel°, K650E/K651E or CTD°, K138E/R492E/K650E/K651E or hel°CTD°) but not ATPase-deficient E132Q mutant (see Figure 1E for ATPase activity) no longer exhibit enhancing activity for *h*MDA5 activated by poly(I:C) in human Huh7.5 cells (Related to Figure 7B).

(G) RNA binding deficient *h*LGP2 mutants (K138E/R492E or hel°, K650E/K651E or CTD°, K138E/R492E/K650E/K651E or hel°CTD°) but not ATPase-deficient E132Q

mutant no longer exhibit enhancing activity for the homopolymerisation-defective *h*MDA5 IE/KR mutant activated by poly(I:C) in human Huh7.5 cells. Cells were transfected with a mixture of LGP2 (27.5 ng DNA/well) and MDA5 (1.67 ng DNA/well), i.e. at 5:0.3 ratio (Related to Figure 7C).

Figure S1

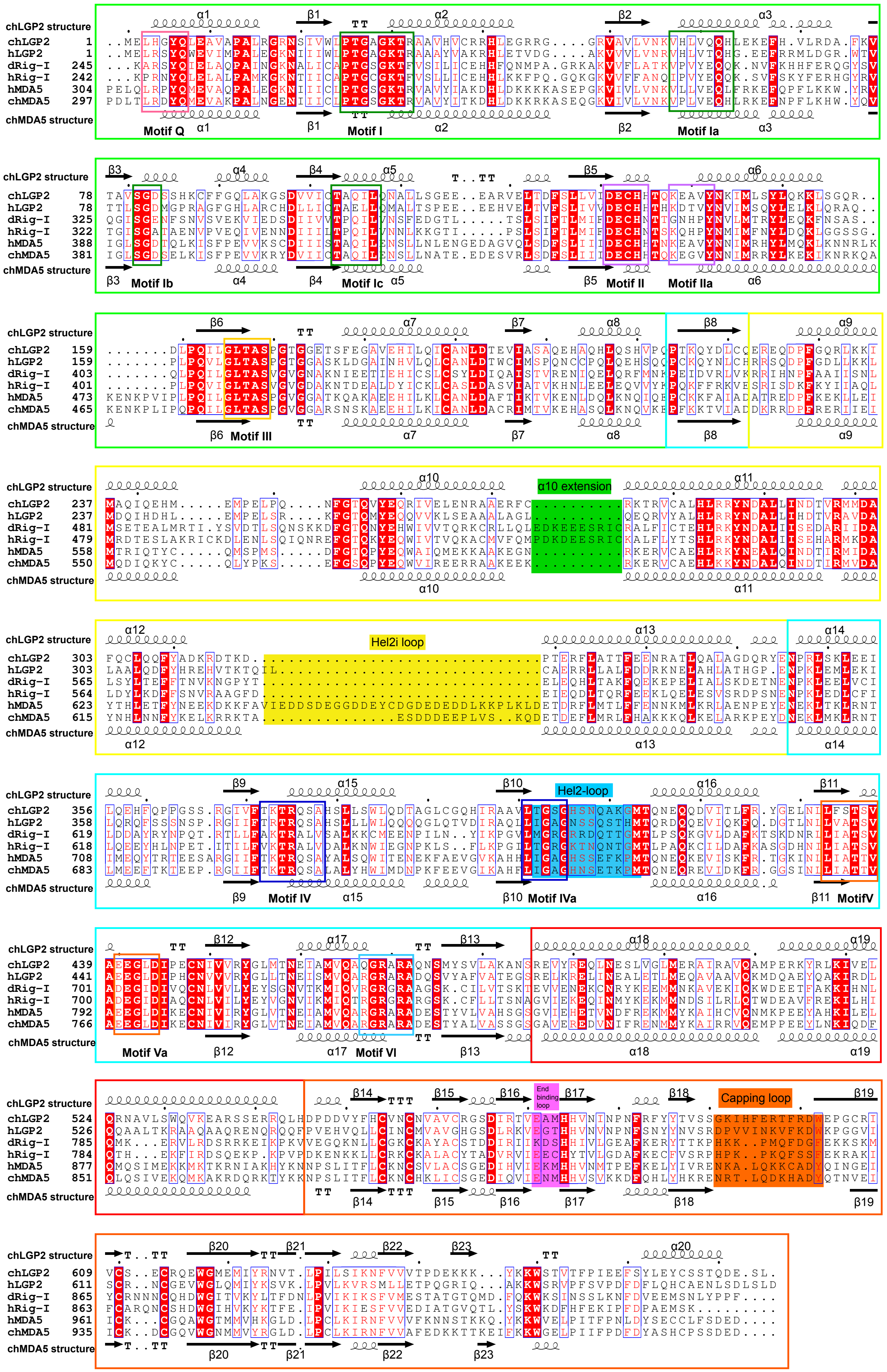
chLGP2 structure
chLGP2
hLGP2
dRig-I
hRig-I
hMDA5
chMDA5
chMDA5 structure
Motif Q
Motif I
Motif Ia
Motif Ib
Motif Ic
Motif II
Motif IIa
Motif III
α10 extension
Hel2i loop
Hel2-loop
Motif IV
Motif IVa
MotifV
Motif Va
Motif VI
End binding loop
Capping loop

Figure S2

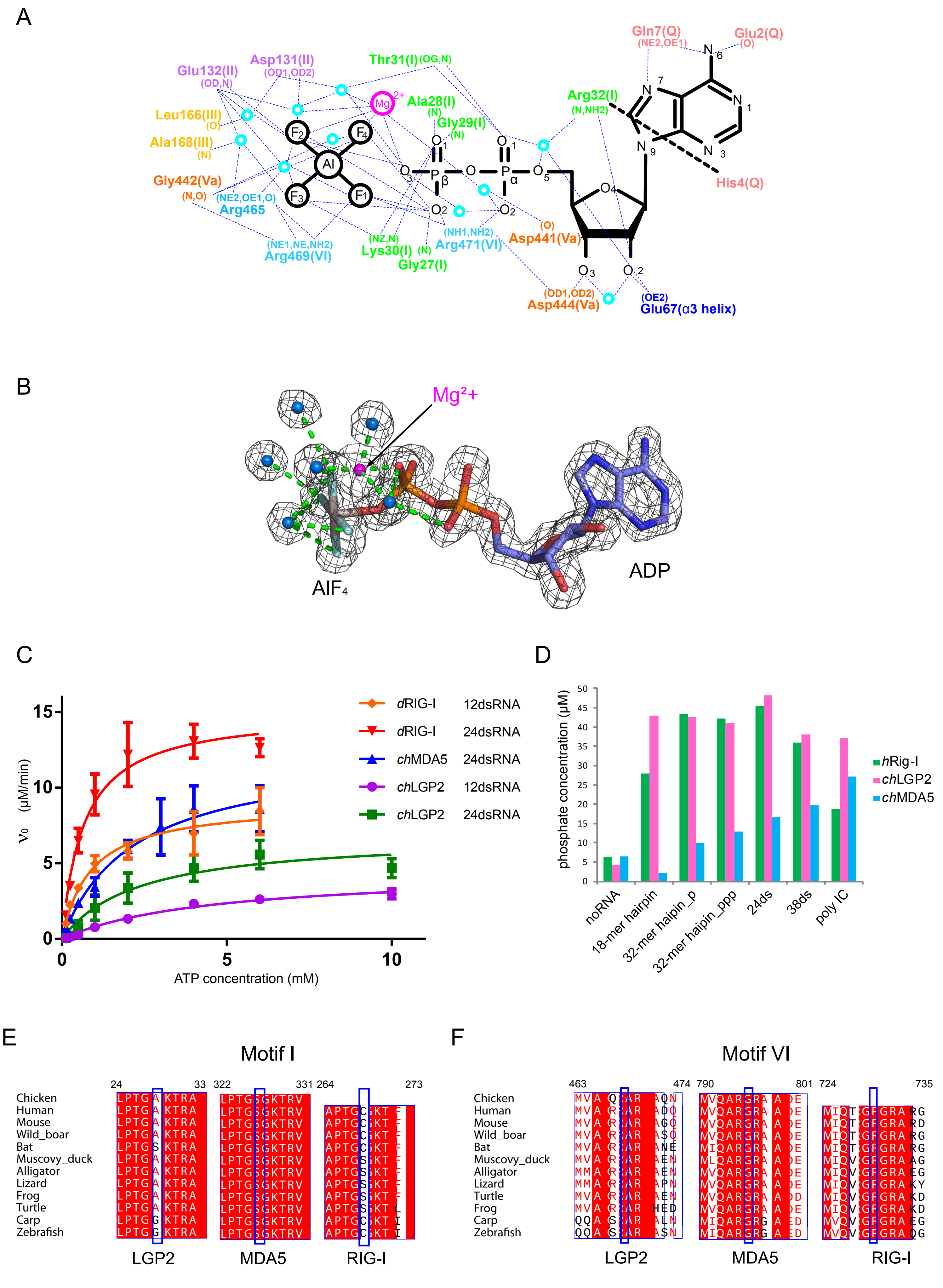
A
Gln7(Q)
(NE2,OE1)
Glu2(Q)
(O)
Asp131(II)
(OD1,OD2)
Thr31(I)(OG,N)
Glu132(II)
(OD,N)
Mg2+
Ala28(I)
(N)
Arg32(I)
(N,NH2)
Leu166(III)
(O)
Gly29(I)
(N)
Ala168(III)
(N)
Al
F1
F2
F3
F4
His4(Q)
Gly442(Va)
(N,O)
(NE2,OE1,O)
Arg465
(O)
Asp441(Va)
(NE1,NE,NH2)
Arg469(VI)
(NZ,N)
Lys30(I)
(N)
Gly27(I)
(NH1,NH2)
Arg471(VI)
(OD1,OD2)
Asp444(Va)
(OE2)
Glu67(α3 helix)
B
Mg²+
AlF4
ADP
C
dRIG-I 12dsRNA
dRIG-I 24dsRNA
chMDA5 24dsRNA
chLGP2 12dsRNA
chLGP2 24dsRNA
v0 (μM/min)
ATP concentration (mM)
D
phosphate concentration (μM)
hRig-I
chLGP2
chMDA5
noRNA
18-mer hairpin
32-mer haipin_p
32-mer haipin_ppp
24ds
38ds
poly IC
E
Motif I
Chicken
Human
Mouse
Wild_boar
Bat
Muscovy_duck
Alligator
Lizard
Frog
Turtle
Carp
Zebrafish
LGP2
MDA5
RIG-I
F
Motif VI
Chicken
Human
Mouse
Wild_boar
Bat
Muscovy_duck
Alligator
Lizard
Turtle
Frog
Carp
Zebrafish
LGP2
MDA5
RIG-I

Figure S3

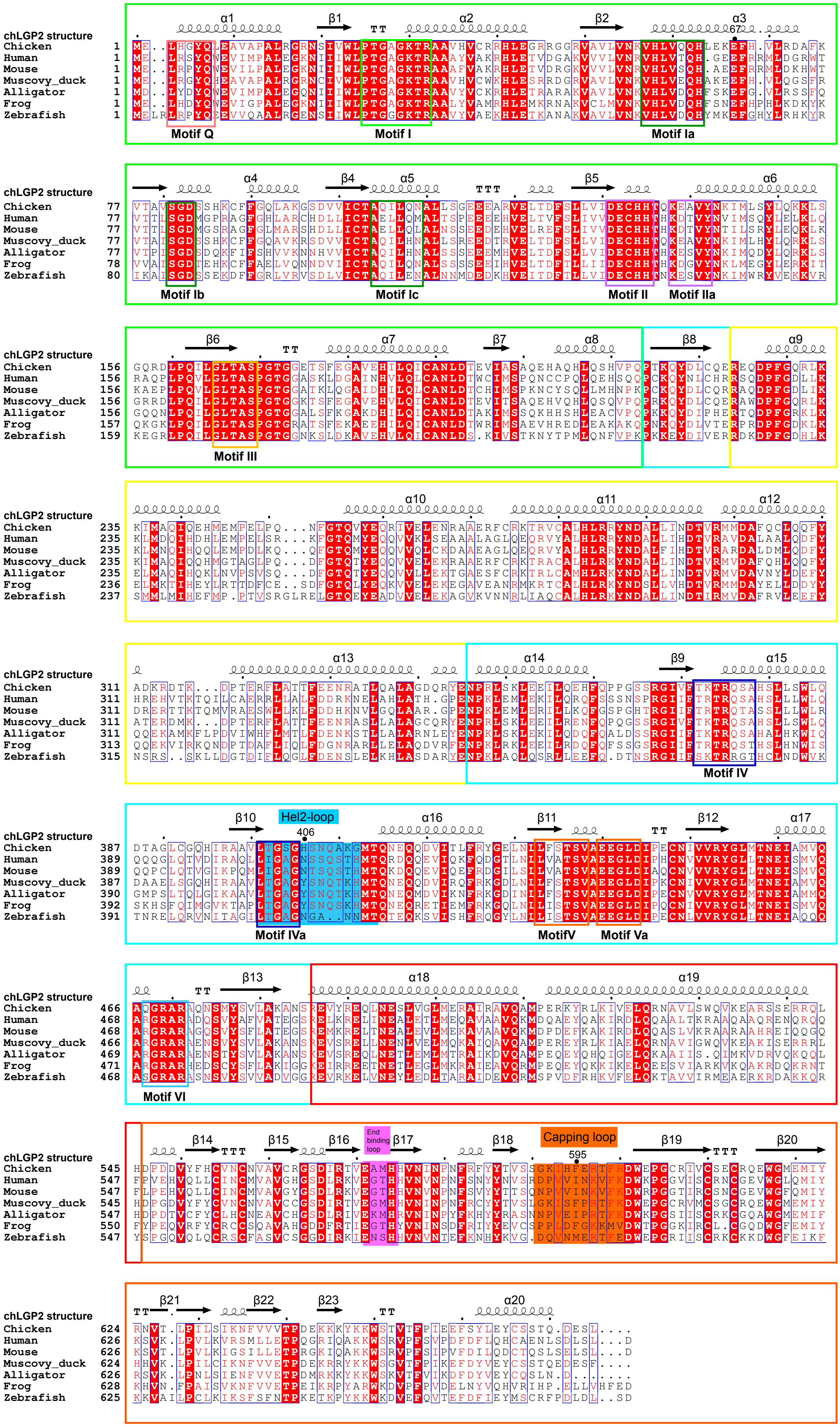

chLGP2 structure
Chicken
Human
Mouse
Muscovy_duck
Alligator
Frog
Zebrafish
Motif Q
Motif I
Motif Ia
Motif Ib
Motif Ic
Motif II
Motif IIa
Motif III
Motif IV
Hel2-loop
Motif IVa
MotifV
Motif Va
Motif VI
End binding loop
Capping loop

Figure S4

A

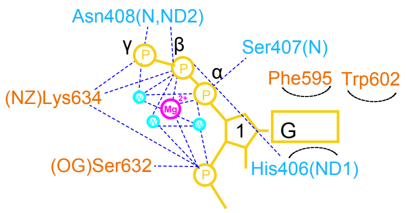

B

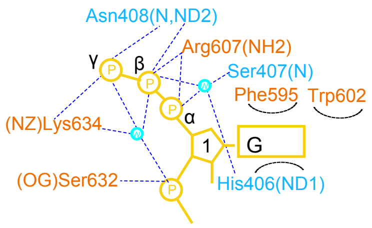

C

Anisotropy	RNA
12 dsRNA$_{OH}$	5′-$_{OH}$GGGCUCCACAUG -3′<br>3′- CCCGAGGUGUAC$_{(FAM)}$-5′
12 dsRNA$_{ppp}$	5′-$_{ppp}$GGGCUCCACAUG -3′<br>3′- CCCGAGGUGUAC$_{(FAM)}$-5′
3’ overhang$_{OH}$	5′- $_{OH}$GGCCAGUGCGAA -3′<br>3′- AACCGGUCACGC$_{(FAM)}$ -5′
5’ overhang$_{OH}$	5′-$_{OH}$AACCGGUCACGC -3′<br>3′- $_{(FAM)}$GGCCAGUGCGAA$_{OH}$-5′
*ch*LGP2 crystallisation	
10 dsRNA$_{p}$	5′- $_{p}$GGUACGUACC -3′<br>3′- CCAUGCAUGG$_{p}$ -5′
10 dsRNA$_{ppp}$	5′- $_{ppp}$GGUACGUACC -3′<br>3′- CCAUGCAUGG$_{ppp}$-5′
12 dsRNA$_{p}$	5′- $_{p}$GGUAGCGCUACC -3′<br>3′- CCAUCGCGAUGG$_{p}$-5′
5′-ppp and 3' two nucleotide (GG) overhang hairpin RNA duplex	5′- $_{ppp}$GGAGCGUGCCG U<br>3′- GGCCUCGCACGGC A
*ch*MDA5 crystallisation	
10 dsRNA$_{p}$	5′- $_{p}$GGUACGUACC -3′<br>3′- CCAUGCAUGG$_{p}$ -5′
24 dsRNA$_{p}$	5′-$_{p}$GGGACGUCAUGCGCAUGACGUCCC -3′<br>3′- CCCUGCAGUACGCGUACUGCAGGG$_{p}$ -5′
26 dsRNA$_{p}$	5′-$_{p}$GGGACGUUCAUGCGCAUGAACGUCCC -3′<br>3′- CCCUGCAAGUACGCGUACUUGCAGGG$_{p}$-5′
27 dsRNA$_{p}$	5′-$_{p}$GGGCACGUGCAGGUCCUGCACGUGCCC -3′<br>3′- CCCGUGCACGUCCAGGACGUGCACGGG$_{p}$-5′

Figure S5

A

B

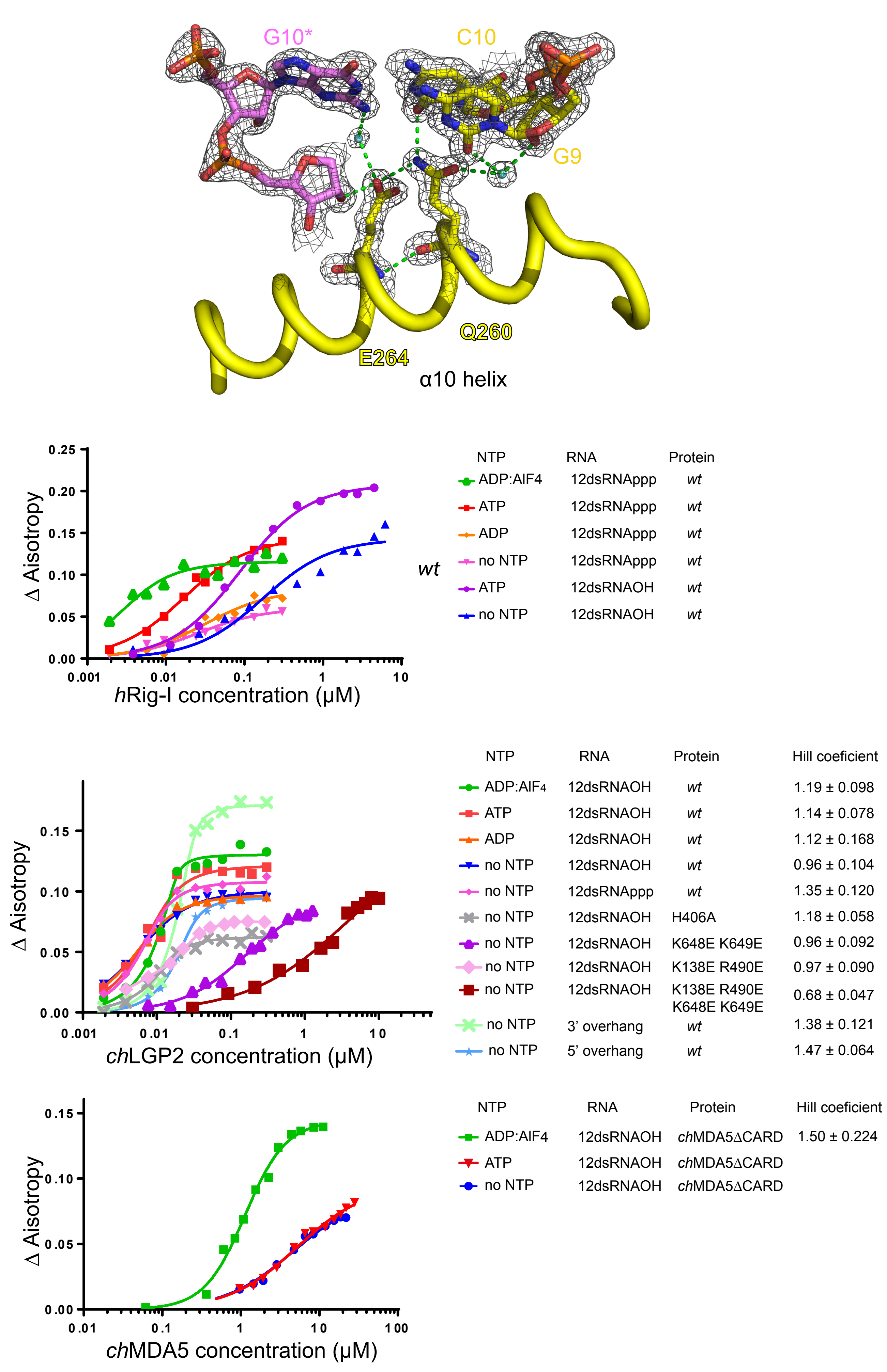

G10*
C10
G9
Q260
E264
α10 helix
Δ Aisotropy
hRig-I concentration (μM)
wt
NTP RNA Protein
ADP:AlF4 12dsRNAppp wt
ATP 12dsRNAppp wt
ADP 12dsRNAppp wt
no NTP 12dsRNAppp wt
ATP 12dsRNAOH wt
no NTP 12dsRNAOH wt
Δ Aisotropy
chLGP2 concentration (μM)
NTP RNA Protein Hill coeficient
ADP:AlF4 12dsRNAOH wt 1.19 ± 0.098
ATP 12dsRNAOH wt 1.14 ± 0.078
ADP 12dsRNAOH wt 1.12 ± 0.168
no NTP 12dsRNAOH wt 0.96 ± 0.104
no NTP 12dsRNAppp wt 1.35 ± 0.120
no NTP 12dsRNAOH H406A 1.18 ± 0.058
no NTP 12dsRNAOH K648E K649E 0.96 ± 0.092
no NTP 12dsRNAOH K138E R490E 0.97 ± 0.090
no NTP 12dsRNAOH K138E R490E K648E K649E 0.68 ± 0.047
no NTP 3' overhang wt 1.38 ± 0.121
no NTP 5' overhang wt 1.47 ± 0.064
Δ Aisotropy
chMDA5 concentration (μM)
NTP RNA Protein Hill coeficient
ADP:AlF4 12dsRNAOH chMDA5ΔCARD 1.50 ± 0.224
ATP 12dsRNAOH chMDA5ΔCARD
no NTP 12dsRNAOH chMDA5ΔCARD

Figure S6

A

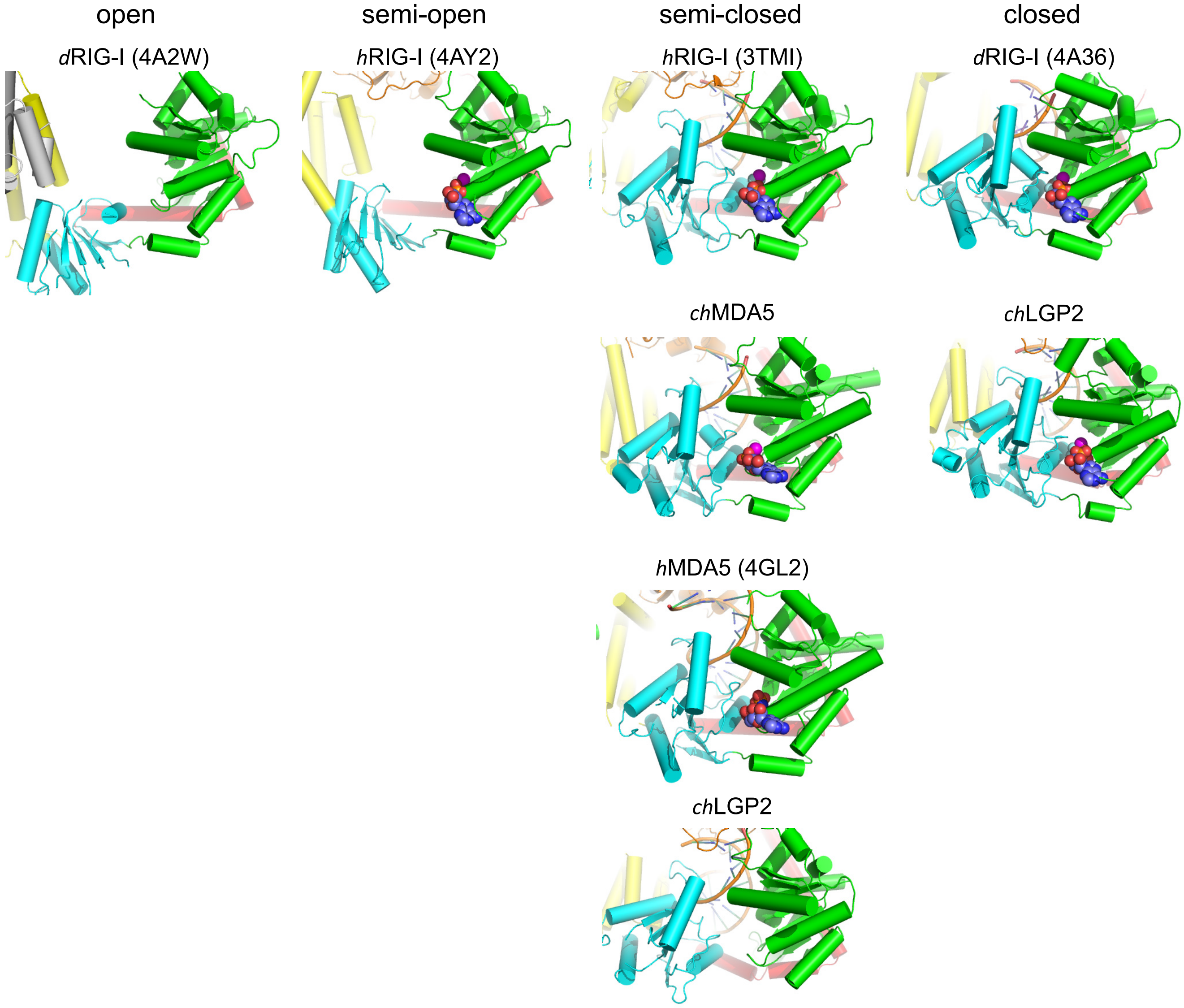

B

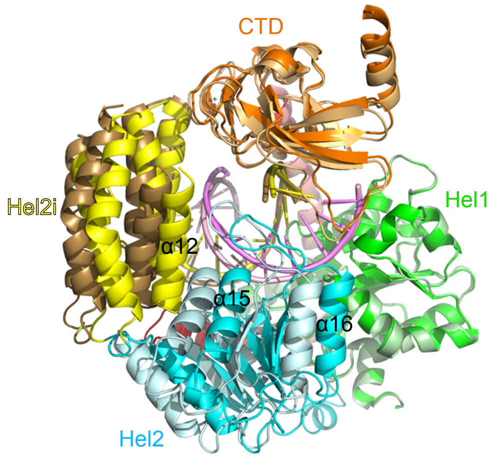

C

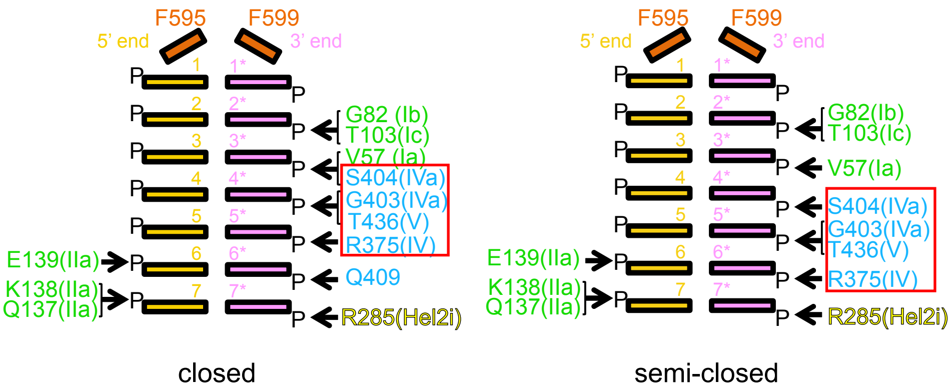

Figure S7

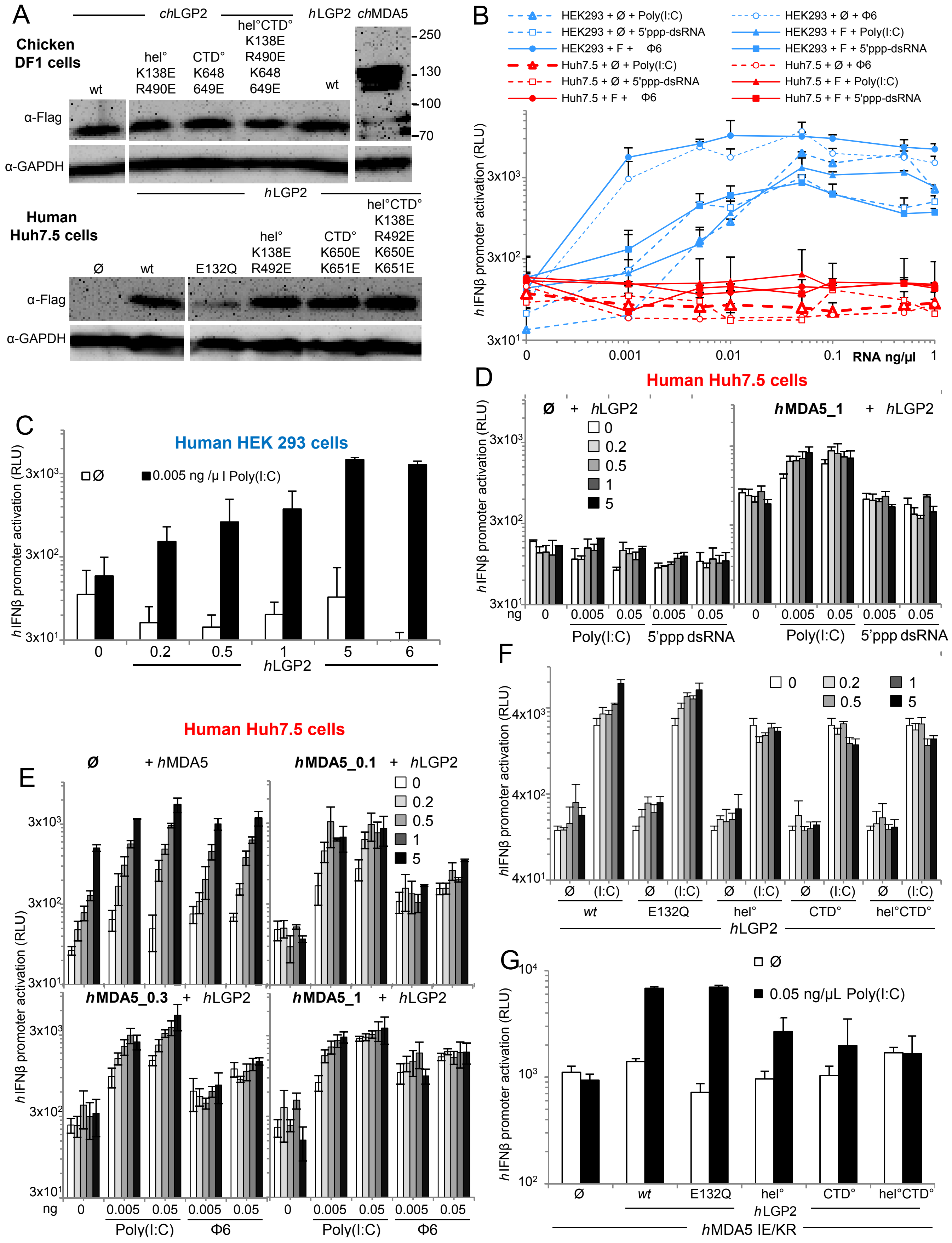

A
chLGP2
hLGP2
chMDA5
Chicken DF1 cells
wt
hel° K138E R490E
CTD° K648 649E
hel°CTD° K138E R490E K648 649E
wt
α-Flag
α-GAPDH
250
130
100
70
hLGP2
Human Huh7.5 cells
Ø
wt
E132Q
hel° K138E R492E
CTD° K650E K651E
hel°CTD° K138E R492E K650E K651E
B
HEK293 + Ø + Poly(I:C)
HEK293 + Ø + 5'ppp-dsRNA
HEK293 + F + Φ6
Huh7.5 + Ø + Poly(I:C)
Huh7.5 + Ø + 5'ppp-dsRNA
Huh7.5 + F + Φ6
HEK293 + Ø + Φ6
HEK293 + F + Poly(I:C)
HEK293 + F + 5'ppp-dsRNA
Huh7.5 + Ø + Φ6
Huh7.5 + F + Poly(I:C)
Huh7.5 + F + 5'ppp-dsRNA
hIFNβ promoter activation (RLU)
RNA ng/µl
C
Human HEK 293 cells
Ø
0.005 ng /µ l Poly(I:C)
hLGP2
D
Human Huh7.5 cells
Ø + hLGP2
hMDA5_1 + hLGP2
Poly(I:C)
5'ppp dsRNA
E
Human Huh7.5 cells
Ø + hMDA5
hMDA5_0.1 + hLGP2
hMDA5_0.3 + hLGP2
hMDA5_1 + hLGP2
Poly(I:C)
Φ6
F
wt
E132Q
hel°
CTD°
hel°CTD°
hLGP2
G
Ø
0.05 ng/µL Poly(I:C)
hMDA5 IE/KR

Table S1. Quantification of the degree of opening (rotation angle and center of mass distance) of the Hel1 and Hel2 helicase domains in published RLH structures. Related to Figures 1, 4 and S6.

RLH Structure	PDB ID	RNA	Bound Nucleotide	Angle (degree)	Distance* (Å)	State
*ch*LGP2 (Reference)	5JAJ	10 base pair 5′p	ADP:AlF_4	0.0	0.0	closed
*d*RIG-I	4A36	19 base pair 5′OH	ADP:AlF_4	2.5	1.3	closed
*h*MDA5	4GL2	12 base pair 5′OH	AMPPNP	8.0	3.3	semi-closed
*ch*MDA5	5JC3	10 base pair 5′p	ADP	10.1	3.9	semi-closed
*h*RIG-I	5E3H (3TMI)	14 base pair 5′OH	ADP:BeF_3	12.1	5.3	semi-closed
*ch*LGP2	5JBJ	12 base pair 5′p	-	13.1	5.4	semi-closed
*h*RIG-I	4AY2	20-mer hairpin 5′ppp	ADP	39.3	14.9	semi-open
*h*RIG-I	2YKG	10 base pair 5′OH	SO_4	40.3	15.9	open
*d*RIG-I	4A2W(A)	no RNA	-	51.8	18.5	open
*d*RIG-I	4A2W(B)	no RNA	-	59.8	19.2	open
*m*RIG-I	3TBK	no RNA	AMPPNP	68.2	24.4	open

Supplementary Experimental Procedures

Protein preparation

***ch*MDA5**. Chicken (*Gallus gallus*) MDA5 constructs (Genbank: NP_001180567) full-length *ch*MDA5 (residues 1-1001), *ch*MDA5_ΔCARD (residues 298-994), *ch*MDA5ΔCARD-Q (residues 298-994 with E436Q mutation) were cloned into pETM11 (EMBL) using a synthetic gene (GeneArt). All constructs were expressed in *E. coli* Rosetta 2 (Novagen) and cells were harvested and lysed by sonication in buffer containing 50 mM Tris pH 7.6, 500 mM NaCl, 10 % glycerol, 20 mM imidazole and 1 mM DTT. After centrifugation the lysate was applied to nickel-NTA-Superflow resin (QIAGEN). The protein was dialyzed against dialysis buffer (20 mM Hepes pH 7.5, 100 mM NaCl, 5% Glycerol, 1 mM DTT) at 4°C for 16 hours. The N-terminal 6-histidine-tag was cleaved with his-tagged Tobacco Etch Virus (TEV) protease during dialysis and cleaved protein repurified on nickel-NTA resin. *ch*MDA5 constructs were further purified with a cation exchange column (HiTrap SP, GE Healthcare) and size-exclusion chromatography (Superdex 200, GE Helthcare) in a buffer containing 20 mM Hepes pH 7.5, 150 mM NaCl and 2 mM DTT.

***ch*LGP2**. A synthetic gene (GeneArt) comprising the coding sequence of *ch*LGP2 (Genbank: AEK21509) with an added N-terminal GGGGS linker was cloned into pETM11SUMO (EMBL). Full-length *ch*LGP2 full length was expressed in *E.coli* Rosetta 2 as a Sumo fusion protein and cells were harvested and lysed by sonication in sonication buffer (50 mM Na_2HPO_4 pH 8, 300 mM NaCl, 5% glycerol, 50 mM arginine, 5 mM β-mercapto-ethanol). After centrifugation the lysate was applied to nickel-NTA-Superflow resin. The protein was dialyzed against sonication buffer at 4°C for 16 hours. The 6-histidine-tag and Sumo-tag was cleaved with TEV protease during dialysis. Cleaved protein was further purified with anion exchange column (HiTrap Q GE Healthcare) and size-exclusion chromatography (Superdex 200, GE Healthcare) in a buffer containing 20 mM Hepes pH 7.5, 200 mM NaCl and 250 µM TCEP (tris(2-carboxyethyl)phosphine).

***h*LGP2**. Full-length *h*LGP2 (Genbank NM_024119.2) was cloned into the pETM11SUMO vector (EMBL) and expressed in *E.coli* and purified as for *ch*LGP2. The chromatograph of a HiTrap Q purification shows two peaks, corresponding to *h*LGP2 monomers or dimers (the majority), as deduced from size-exclusion chromatography.

RIG-I. Full-length *h*RIG-I was cloned into pFastBAC and expressed in HiFive insect cells. Cells were harvested and lysed by sonication in buffer (50 mM Tris pH7.6, 500 mM NaCl, 10% glycerol, 20 mM Imidazole, 1 mM DTT). After centrifugation the lysate was applied to nickel-NTA-Superflow resin and further purified as for *ch*LGP2. Full-length *d*RIG-I was purified from insect cells as described (Kowalinski et al., 2011).

Crystallization

For protein crystallization, 576 different conditions were initially screened in vapour diffusion sitting drop format using a Cartesian robot. After screening, the crystal conditions were optimized in 24-well hanging drop crystallization plates.

***ch*MDA5 1:1 complex:** Directly after size exclusion chromatography *ch*MDA5ΔCARD-Q was mixed with 10 bp dsRNA in a 1:1 molar ratio and incubated for 30 minutes on ice. The complex was concentrated using an Amicon Ultra concentrator to around 10 mg/ml and 2 mM AMPPNP (adenosine 5′-(β,γ-imido)triphosphate lithium salt hydrate) was added. Sample and reservoir buffer (0.025 M Bis-Tris pH 6.5, 0.075 M succinic acid pH 7.0, 12-14% PEG 3350, 2% sucrose) were mixed in a 2:1 ratio. Three hours after setup, cover glasses with drops were transferred from a reservoir containing 12-14% PEG 3350 to one containing 8% PEG 3350. Crystals grew in one week at 20°C and were harvested in cryo-protectant solution (0.025 M Bis-Tris pH 6.5, 0.075 M succinic acid pH 7.0, 25% 3350, 10% ethylene glycol) before flash freezing with liquid nitrogen.

***ch*MDA5 2:1 complexes:** Directly after size exclusion chromatography *ch*MDA5ΔCARD-Q was mixed with 24, 26 or 27 bp dsRNA in a 0.5:1 molar ratio and incubated for 30 minutes

on ice. The complexes were concentrated with an Amicon Ultra concentrator to around 10 mg/ml and 2 mM AMPPNP and 200 mM NDSB211 (Dimethyl(2-hydroxyethyl)ammonium propane sulfonate, Hampton Research) were added.

***ch*MDA5ΔCARD-Q 24-mer dsRNA-AMPPNP** was mixed with reservoir buffer (0.1 mM Hepes pH 7.5, 11-12% PEG 3350, 2-4% sucrose) in a 2:1 ratio. Crystals grew in three days at 20 degree and were harvested in cryo-protecting solution (0.1 mM Hepes pH 7.5, 25% 3350, 10% ethylene glycol) before flash freezing with liquid nitrogen.

***ch*MDA5ΔCARD-Q : 27-mer dsRNA-AMPPNP** was mixed with reservoir buffer (0.1 mM Hepes pH 7.5, 4-6% PEG 6000, 2-4% sucrose) in a 2:1 ratio. Crystals grew in three days at 20°C. The cover glasses of crystal-containing drops were transferred to a reservoir containing cryo-protecting solution (0.1 mM Hepes pH 7.5, 10% PEG 6000, 25% ethylene glycol) 12 hours before harvesting and flash freezing with liquid nitrogen.

***ch*LGP2:** *ch*LGP2, directly after size exclusion chromatography, was mixed with dsRNA in a 1:1 ratio and incubated for 30 minutes on ice. The complexes were concentrated with an Amicon Ultra concentrator to around 10 mg/ml and then 2 mM ADP:AlF_4 (adenosine-5′-diphosphate: aluminium fluoride) and 2 mM $MgCl_2$ were added. All *ch*LGP2 crystals were harvested in the reservoir buffer containing 25% ethylene glycol and flash frozen with liquid nitrogen.

***ch*LGP2 12-mer dsRNA-ADP:AlF_4** complex was mixed with reservoir buffer (0.1 M Bis-Tris propane pH 6.5, 0.05-0.1 M magnesium formate) in a 2:1 ratio. Crystals grew in three days at 4°C.

***ch*LGP2 5′ monophosphate 10-mer dsRNA-ADP:AlF_4** complex was mixed with reservoir buffer (0.1 M tri-sodium citrate dihydrate pH 6.1, 0.04-0.08 M ammonium dihydrogen phosphate) in a 2:1 ratio. Crystals grew in one week at 4°C.

***ch*LGP2 5′ triphosphate 10-mer dsRNA-ADP:AlF_4** complex was mixed with reservoir

buffer (0.1 M Mg formate, 20% PEG3350M) in a 2:1 ratio. Crystals grew in one week at 4°C. **chLGP2 5′ triphosphate, 3′-GG overhang hairpin RNA-ADP:AlF4** complex was mixed with reservoir buffer (0.1 M citric acid pH 5.0, 0.8 M ammonium sulphate) in a 1:1 ratio. Crystals grew in one day at 4°C from a sitting drop 96 well plate.

Crystallography

Diffraction data were collected at 100 K on beamlines ID23-1 or ID29 equipped with a Pilatus 6M detector or ID23-2 equipped with a Pilatus 2M-F (DECTRIS), at the European Synchrotron Radiation Facility (ESRF), Grenoble, France. Data were processed with the XDS suite and scaled with XSCALE (Kabsch, 2010). All further analysis was done with the CCP4 suite (Winn et al., 2011) and refinement performed with REFMAC5 (Murshudov, 1997) or PHENIX (Adams et al., 2002). Models were built using COOT (Emsley and Cowtan, 2004), structure validation by MOLPROBITY (Chen et al., 2010) and structure figures drawn with PYMOL (DeLano, 2002). Buried surface are calculations performed with PISA (http://www.ebi.ac.uk/pdbe/prot_int/pistart.html) (Krissinel and Henrick, 2007). Crystallographic and refinement statistics are given in Tables 1 and 2.

The *ch*MDA5 structure was initially determined by molecular replacement with PHASER (McCoy et al., 2007) using the *h*MDA5 structure (PDB 4GL2). The *ch*LGP2 structure was initially determined with PHASER using the *h*LGP2 CTD (PDB: 2W4R) and RIG-I helicase sub-domains as search models.

Crystals of MDA5 with 10- and 24-mer dsRNAs were of highly variable quality, requiring testing of many to obtain usable data. For the 10-mer complex, several related space-groups were obtained with two ($P2_1$ or $P2_12_12_1$) or four (large $P2_1$) 1:1 complexes in the asymmetric unit. Both 10- and 24-mer crystals were generally twinned (probably lattice translocation disorder), this being manifested by weak but significant residual extra density after refinement that could be explained by the same model but referred to a different origin.

One 10-mer crystal in the $P2_1$ form was not twinned and gave a cleaner map and lower R-factors (Table 2).

RNA preparation

The palindromic 10 bp dsRNA with 5′ mono-phosphate or 5′ tri-phosphate (5′-pGGUACGUACC-3′ or 5′-pppGGUACGUACC-3′), 12 bp dsRNA with 5′ mono-phosphate (5′-pGGUAGCGCUACC-3′), 24 bp dsRNA with 5′ mono-phosphate (5′-pGGGACGUCAUGCGCAUGACGUCCC-3′) and 27 bp dsRNA with 5′ monophosphate (5′-pGGGCACGUGCAGGACCUGCACGUGCCC-3′) and 5′ triphosphate 3′-GG overhang 26-mer hairpin RNA (5′-pppGGAGCGUGCCGUACGGCACGCUCCGG-3′) were prepared by *in vitro* T7 transcription. Five times excess of GMP over GTP was added to the reaction for the 5′ mono-phosphate RNAs. RNA was purified by excising the band from denaturing urea-PAGE. A summary of RNAs used is shown in Figure S4.

Electron microscopy

Φ6 RNA (Thermo Scientific) is a mixture of 2948 bp (S segment), 4063 bp (M), and 6374 bp (L) dsRNAs. To form complexes, full-length *ch*MDA5 or *ch*LGP2 and Φ6 bacteriophage dsRNA were incubated during 5 min at 20°C in a buffer containing 20 mM Hepes pH 7.5, 150 mM NaCl, 2 mM ATP and 2 mM $MgCl_2$. Different protein:RNA ratios were used with a 1:1 ratio of MDA5/LGP2:dsRNA corresponding to one MDA5/LGP2 molecule for 15 bp of dsRNA. Where indicated, ATP was replaced by ADP, ADP:AlF_4 or no NTP analogues in order to test their influence.

For each condition tested, 4 µl of sample was applied to the clear side of carbon on a carbon-mica interface and stained with 1% (wt/vol) uranyl acetate. Images were recorded under low-dose conditions with a JEOL 1200 EX II microscope at 100 kV with a nominal magnification of 15,000×.

Cryo-EM grids were prepared by mixing 4 µl of MDA5 or LGP2 at 5 µM with

dsRNA in a 1:1 ratio. The sample was applied onto a glow-discharged quantifoil grid 400 mesh 3.5/1 (Quantifoil Micro Tools GmbH, Germany), the excess solution was blotted for 2 s with a Vitrobot (FEI) and the grid frozen in liquid ethane. Data collection was performed on a FEI Polara microscope operated at 300 kV. Fifty cryoEM micrographs were manually collected on a K2 summit electron direct detector (Gatan) at a magnification of 20,000×, giving a pixel size of 0.97 Å. Movies of 6 frames were collected with a total exposure time of 6 s and a total dose of $30e^{-}/Å^{2}$. The contrast transfer function (CTF) for each micrograph was determined with CTFFIND3 (Mindell and Grigorieff, 2003) and showed defocuses between 2.2 and 3.8 µm. A total of 7185 overlapping segments of MDA5 and 3153 overlapping segments of LGP2 (400 pixels long) were picked using EMAN boxer (Ludtke et al., 1999) with a shift of 64 pixels between adjacent segments. Corresponding segments were suggested to 2D classification in Relion1.3 (Scheres, 2012). Power spectra were calculated from masked 2D class averages.

Fluorescence polarisation anisotropy

dsRNAs used for anisotropy experiments were formed by annealing the following separate strands for 1 min at 95 °C and cooling down on ice in a buffer containing 20 mM Hepes, 2 mM EDTA at pH 6.8 (see also Figure S5 for RNA sequences):

12-mer dsRNA : 5′-$_{FAM}$CAUGUGGAGCCC-3′ and 5′-$_{OH}$GGGCUCCACAUG-3′,

12-mer dsRNAppp : 5′-$_{FAM}$CAUGUGGAGCCC-3′ and 5′-$_{ppp}$GGGCUCCACAUG-3,

3′ overhang dsRNA : 5′-$_{OH}$GGCCAGUGCGAA-3′ and 5′-$_{FAM}$CGCACUGGCCAA-3′,

5′ overhang dsRNA : 5′-$_{OH}$AACCGGUCACGC3′ and 5′-$_{OH}$AAGCGUGACCGG$_{FAM}$-3′.

Anisotropy measurements were performed at room temperature using a multimodular fluorometer (Quantamaster QM4CW; Manufacturer: Photon Technology International). RNA concentration was adjusted to 2.5 nM (12-mer 5′ppp-dsRNA) or 5 nM (12-mer 5′OH-dsRNA, 3′ 2 nt overhang dsRNA and 5′ 2 nt overhang dsRNA for LGP2 measurements), 13

or 50 nM (12-mer 5′OH-dsRNA for MDA5 measurements) by mixing in the cuvette with buffer (20 mM Hepes pH 7.5 150 mM NaCl, 200 µM TCEP, 4 mM $MgCl_2$). Protein was titrated into the cuvette and incubated for 5 minutes before measurements. The total volume of titrated protein was less than 10% of the final volume. Anisotropy measurements were made with excitation wavelength 495 nm and emission wavelength 515 nm for 100 seconds. Experiments were performed more than three times per system. Binding data was analysed by Graph Pad Prism software and fitted with the following equation (eq. 1).

$$y = \frac{(\mathrm{R}+P^n+Kd^n)-\sqrt{(R+P^n+kd^n)^2-4RP^n}}{2R} \times b + (m-b) \qquad \text{(eq. 1)}$$

The experimental anisotropy (y), Initial anisotropy (b), the maximum anisotropy (m), the RNA concentration (R), and the protein concentration (P), equilibrium binding constant (*K*d) and hill coefficient (n).Rig-I measurement curves are fitted with n=1 and the value of n are written in Figure S4. The error bars represent the S.D. value of at least three individual experiments.

ATPase activity assays

ATPase reactions were monitored using a Malachite green assay kit (Bioassays) over 0.5-30 min time courses. All proteins were pre-incubated with 4-fold molar excess of dsRNA for 10 minutes at 28 °C in ATP hydrolysis buffer (20 mM Hepes pH7.5, 100 mM NaCl, 1% glycerol, 2 mM DTT, 4 mM $MgCl_2$). The reaction was initiated by adding 2 mM ATP. For determination of kinetic parameters, the ATP concentration was varied from 0.125 to 10 mM. The reactions were quenched at 5 time points between 30 seconds to 4 minutes. The 10 µl reaction aliquots were quenched by mixing with 10 µl of quenching buffer (20 mM Hepes pH 7.5, 100 mM NaCl, 1% glycerol, 2 mM DTT, 100 mM EDTA). 80 µl of 5 times diluted Malachite green solution was added and developed for 30 minutes at room temperature. The absorbance at 622 nm was measured with a plate reader. For each ATP concentration, the

initial velocity was plotted and *K*m and *k*cat calculated using the Michaelis–Menten equation (eq. 2) in Graph Pad software Prism.

Y=Et*kcat*X/(*K*m+X) (eq. 2)

X is the substrate concentration, Y is enzyme velocity and Et is the concentration of enzyme.

Cellular assays.

Myc-*h*MDA5 and Flag-*h*RIG-I constructs in pEF-BOS vector have been described previously (Kowalinski et al., 2011; Louber et al., 2015; Louber et al., 2014) and *ch*MDA5, *ch*LGP2, *h*LGP2 tagged at the N terminus with Flag peptide were sub-cloned into pCDNA3 (chicken) and pEF-BOS (human) vector as detailed elsewhere (Louber et al., 2015; Louber et al., 2014).

Chicken DF1 (Himly et al., 1998) and human Huh7.5 (Sumpter et al., 2005) cells were maintained in Dulbecco's Minimum Essential Medium culture medium supplemented with 10% foetal calf serum, 10 mM HEPES, 2 mM L-glutamine, 10 mg/ml gentamycin and 1% non-essential amino acids for Huh7.5 cells at 37°C and 5% CO2. Being of chicken origin, DF1 cells naturally lack endogenous RIG-I (Barber et al., 2010), but express functional endogenous MDA5, LGP2 and type I IFN system (Karpala et al., 2011; Liniger et al., 2012). Huh7.5 cell line is defective in MDA5 and IFN receptor expression and expresses inactive T55I RIG-I (Binder et al., 2011; Eguchi et al., 2000; Keskinen et al., 1999; Li et al., 2005; Sumpter et al., 2005), a phenotype verified experimentally by the inability of these cells to respond to both RIG-I and MDA5 RNA agonists (Figure S7 and our previous data (Louber et al., 2015)).

Protein expression was analysed after lysis of DF1 and Huh7.5 cells (6-well plate, 6x105 cells/well) transfected the day before with 2.5 µg DNA using TransIT®-LT1 Transfection Reagent (Mirus Bio) (7.5 µl/well), separation by polyacrylamide gel

electrophoresis and visualisation by western immunoblotting using anti-Flag (1:1,000; M2; Sigma, St. Louis, MO, USA), anti-C-Myc (1:50) 9E10, anti-GAPDH (1:2,000; Millipore, Billerica, MA, USA) monoclonal antibodies

The human IFNβ promoter luciferase assay was performed essentially as described (Louber et al., 2015; Louber et al., 2014). Briefly, Huh7.5 cells seeded one day before (96 wells, 2 x104 cells/well) were co-transfected with MDA5 and LGP2 expressing vectors (33 ng of DNA/well to which was added 17 ng of DNA/well of pHRL-tk-Renilla-Luc (Promega)) together with the reporter pβIFN-fl-lucter (King and Goodbourn, 1994) (50 ng of DNA/well), using TransIT®-LT1 Transfection Reagent (Mirus Bio) (0.3 µl /well), followed 24 hours later by transfection of either poly(I:C) (Amersham Biosciences), Φ6 dsRNA (Thermo Scientific) or T7-transcribed 61-mer 5′ppp-dsRNA (Marq et al., 2011) in complex with Oligofectamine™ Transfection Reagent (Invitrogen) (0.6 µl/well). The chicken IFNβ promoter luciferase assay was performed following exactly the same procedure, but using chicken adapted material, i.e. DF1 cells, pCDNA3 based expression vectors for MDA5 and LGP2, pHRL-CMV-Renilla-Luc (Promega) instead of pHRL-tk-Renilla-Luc, the reporter pGL3-P_*ch*IFNβ-Luc (Liniger et al., 2012). For both human and chicken assay, control experiments were included by transfecting the same amount of a neutral plasmid (pCG-duF) (Cathomen et al., 1995). This plasmid was also used to complement transfected DNA so as to reach total DNA amount of 100 ng per well. Data were expressed as mean +/-SD of normalized luciferase activity. Statistical analysis (Student t test) was done on data from three independent experiments, each done in independent triplicates.

Supplementary References

Adams, P.D., Grosse-Kunstleve, R.W., Hung, L.W., Ioerger, T.R., McCoy, A.J., Moriarty, N.W., Read, R.J., Sacchettini, J.C., Sauter, N.K., and Terwilliger, T.C. (2002). PHENIX: building new software for automated crystallographic structure determination. Acta Crystallogr D Biol Crystallogr *58*, 1948-1954.

Barber, M.R., Aldridge, J.R., Jr., Webster, R.G., and Magor, K.E. (2010). Association of RIG-I with innate immunity of ducks to influenza. Proc Natl Acad Sci U S A *107*, 5913-5918.

Binder, M., Eberle, F., Seitz, S., Mucke, N., Huber, C.M., Kiani, N., Kaderali, L., Lohmann, V., Dalpke, A., and Bartenschlager, R. (2011). Molecular mechanism of signal perception and integration by the innate immune sensor retinoic acid-inducible gene-I (RIG-I). The Journal of biological chemistry *286*, 27278-27287.

Cathomen, T., Buchholz, C.J., Spielhofer, P., and Cattaneo, R. (1995). Preferential initiation at the second AUG of the measles virus F mRNA: a role for the long untranslated region. Virology *214*, 628-632.

Chen, V.B., Arendall, W.B., 3rd, Headd, J.J., Keedy, D.A., Immormino, R.M., Kapral, G.J., Murray, L.W., Richardson, J.S., and Richardson, D.C. (2010). MolProbity: all-atom structure validation for macromolecular crystallography. Acta Crystallogr D Biol Crystallogr *66*, 12-21.

DeLano, W.L. (2002). PyMOL Molecular Graphics System. available online at http://wwwpymolsourceforgenet.

Eguchi, H., Nagano, H., Yamamoto, H., Miyamoto, A., Kondo, M., Dono, K., Nakamori, S., Umeshita, K., Sakon, M., and Monden, M. (2000). Augmentation of antitumor activity of 5-fluorouracil by interferon alpha is associated with up-regulation of p27Kip1 in human hepatocellular carcinoma cells. Clinical cancer research : an official journal of the American Association for Cancer Research *6*, 2881-2890.

Emsley, P., and Cowtan, K. (2004). Coot: model-building tools for molecular graphics. Acta Crystallogr D Biol Crystallogr *60*, 2126-2132.

Gouet, P., Courcelle, E., Stuart, D.I., and Metoz, F. (1999). ESPript: analysis of multiple sequence alignments in PostScript. Bioinformatics (Oxford, England) *15*, 305-308.

Himly, M., Foster, D.N., Bottoli, I., Iacovoni, J.S., and Vogt, P.K. (1998). The DF-1 chicken fibroblast cell line: transformation induced by diverse oncogenes and cell death resulting from infection by avian leukosis viruses. Virology *248*, 295-304.

Kabsch, W. (2010). Xds. Acta Crystallogr D Biol Crystallogr *66*, 125-132.

Karpala, A.J., Stewart, C., McKay, J., Lowenthal, J.W., and Bean, A.G. (2011). Characterization of chicken Mda5 activity: regulation of IFN-beta in the absence of RIG-I functionality. Journal of immunology *186*, 5397-5405.

Keskinen, P., Nyqvist, M., Sareneva, T., Pirhonen, J., Melen, K., and Julkunen, I. (1999). Impaired antiviral response in human hepatoma cells. Virology *263*, 364-375.

King, P., and Goodbourn, S. (1994). The beta-interferon promoter responds to priming through multiple independent regulatory elements. The Journal of biological chemistry *269*, 30609-30615.

Kowalinski, E., Lunardi, T., McCarthy, A.A., Louber, J., Brunel, J., Grigorov, B., Gerlier, D., and Cusack, S. (2011). Structural basis for the activation of innate immune pattern-recognition receptor RIG-I by viral RNA. Cell *147*, 423-435.

Krissinel, E., and Henrick, K. (2007). Inference of macromolecular assemblies from crystalline state. Journal of molecular biology *372*, 774-797.

Li, K., Chen, Z., Kato, N., Gale, M., Jr., and Lemon, S.M. (2005). Distinct poly(I-C) and virus-activated signaling pathways leading to interferon-beta production in hepatocytes. The Journal of biological chemistry *280*, 16739-16747.

Liniger, M., Summerfield, A., Zimmer, G., McCullough, K.C., and Ruggli, N. (2012). Chicken cells sense influenza A virus infection through MDA5 and CARDIF signaling involving LGP2. Journal of virology *86*, 705-717.

Louber, J., Brunel, J., Uchikawa, E., Cusack, S., and Gerlier, D. (2015). Kinetic discrimination of self/non-self RNA by the ATPase activity of RIG-I and MDA5. BMC biology *13*, 54.

Louber, J., Kowalinski, E., Bloyet, L.M., Brunel, J., Cusack, S., and Gerlier, D. (2014). RIG-I Self-Oligomerization Is Either Dispensable or Very Transient for Signal Transduction. PloS one *9*, e108770.

Ludtke, S.J., Baldwin, P.R., and Chiu, W. (1999). EMAN: semiautomated software for high-resolution single-particle reconstructions. J Struct Biol *128*, 82-97.

Marq, J.B., Hausmann, S., Veillard, N., Kolakofsky, D., and Garcin, D. (2011). Short double-stranded RNAs with an overhanging 5' ppp-nucleotide, as found in arenavirus genomes, act as RIG-I decoys. The Journal of biological chemistry *286*, 6108-6116.

McCoy, A.J., Grosse-Kunstleve, R.W., Adams, P.D., Winn, M.D., Storoni, L.C., and Read, R.J. (2007). Phaser crystallographic software. J Appl Crystallogr *40*, 658-674.

Mindell, J.A., and Grigorieff, N. (2003). Accurate determination of local defocus and

specimen tilt in electron microscopy. J Struct Biol *142*, 334-347.

Murshudov, G.N. (1997). Refinement of macromolecular structures by the maximum-likelihood method. Acta Crystallogr D Biol Crystallogr *53*, 240-255.

Scheres, S.H. (2012). RELION: implementation of a Bayesian approach to cryo-EM structure determination. J Struct Biol *180*, 519-530.

Sumpter, R., Jr., Loo, Y.M., Foy, E., Li, K., Yoneyama, M., Fujita, T., Lemon, S.M., and Gale, M., Jr. (2005). Regulating intracellular antiviral defense and permissiveness to hepatitis C virus RNA replication through a cellular RNA helicase, RIG-I. Journal of virology *79*, 2689-2699.

Winn, M.D., Ballard, C.C., Cowtan, K.D., Dodson, E.J., Emsley, P., Evans, P.R., Keegan, R.M., Krissinel, E.B., Leslie, A.G., McCoy, A., *et al.* (2011). Overview of the CCP4 suite and current developments. Acta Crystallogr D Biol Crystallogr *67*, 235-242.
