# Supplementary material for: Structural Analysis of dsRNA Binding to Anti-viral Pattern Recognition Receptors LGP2 and MDA5
Source: Mol Cell. 2016 May 19;62(4):586–602. doi: 10.1016/j.molcel.2016.04.021 (PMC4885022; doi:10.1016/j.molcel.2016.04.021)
Supplement: Document S2. Article plus Supplemental Information [file mmc2.pdf]

Article

Molecular Cell

Structural Analysis of dsRNA Binding to Anti-viral Pattern Recognition Receptors LGP2 and MDA5

Graphical Abstract

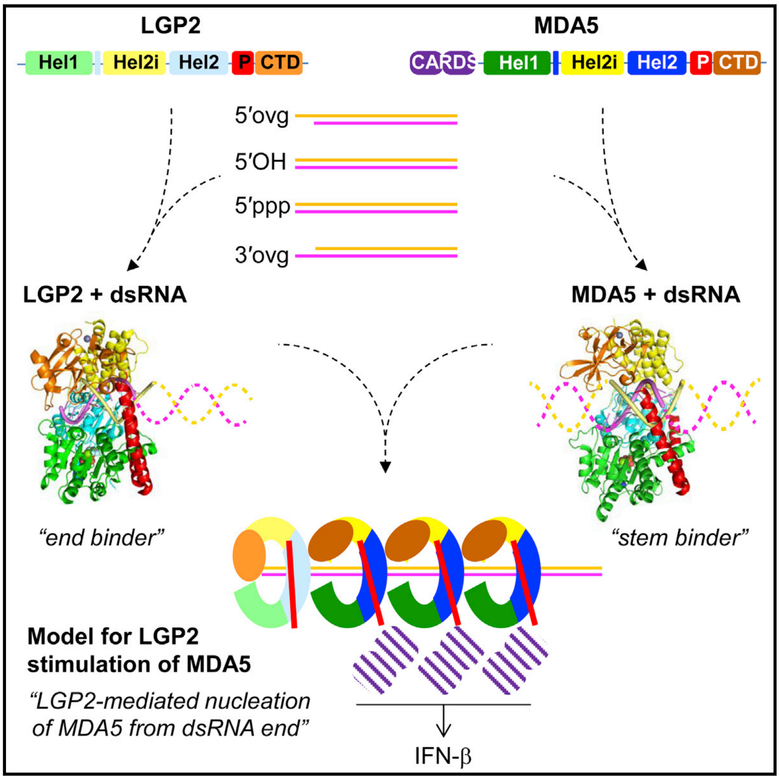

Authors

Emiko Uchikawa, Mathilde Lethier, Hélène Malet, Joanna Brunel, Denis Gerlier, Stephen Cusack

Correspondence

cusack@embl.fr

In Brief

Uchikawa et al. reveal structural details of dsRNA recognition by MDA5 and LGP2 that synergistically sense viral RNA and activate interferon expression. LGP2 is primarily a dsRNA end binder but can also coat dsRNA, but less co-operatively than MDA5. Functional studies show that LGP2 enhancement of MDA5 signaling is RNA dependent.

Highlights

- *ch*LPG2-dsRNA structures reveal RIG-I like end binding, but overhangs are possible
- *ch*MDA5-dsRNA complex structures show head-to-head packing on short dsRNAs
- LGP2 also has MDA5-like behavior, coating dsRNA but with less cooperativity
- Both human and chicken LGP2 enhance MDA5 signaling in an RNA-dependent manner

Accession Numbers

5JAJ, 5JB2, 5JBG, 5JBJ, 5JC3, 5JCH, 5JCF, 5JC7

Uchikawa et al., 2016, Molecular Cell *62*, 586–602
May 19, 2016 © 2016 The Author(s). Published by Elsevier Inc.
http://dx.doi.org/10.1016/j.molcel.2016.04.021

Molecular Cell
Article

Structural Analysis of dsRNA Binding to Anti-viral Pattern Recognition Receptors LGP2 and MDA5

Emiko Uchikawa,[1,2] Mathilde Lethier,[1,2] Hélène Malet,[1,2] Joanna Brunel,[3,4,5,6,7] Denis Gerlier,[3,4,5,6,7] and Stephen Cusack[1,2,*]

[1]European Molecular Biology Laboratory, Grenoble Outstation, 71 Avenue des Martyrs, CS 90181, 38042 Grenoble Cedex 9, France
[2]University Grenoble Alpes, Centre National de la Recherche Scientifique, EMBL Unit of Virus Host-Cell Interactions, 71 Avenue des Martyrs, CS 90181, 38042 Grenoble Cedex 9, France
[3]CIRI, International Center for Infectiology Research, Université de Lyon, 69007 Lyon, France
[4]Inserm, U1111, 69007 Lyon, France
[5]CNRS, UMR5308, 69007 Lyon, France
[6]Ecole Normale Supérieure de Lyon, 69007 Lyon, France
[7]Université Lyon 1, Centre International de Recherche en Infectiologie, 69007 Lyon, France
*Correspondence: cusack@embl.fr
http://dx.doi.org/10.1016/j.molcel.2016.04.021

SUMMARY

RIG-I and MDA5 sense virus-derived short 5′ppp blunt-ended or long dsRNA, respectively, causing interferon production. Non-signaling LGP2 appears to positively and negatively regulate MDA5 and RIG-I signaling, respectively. Co-crystal structures of chicken (*ch*) LGP2 with dsRNA display a fully or semi-closed conformation depending on the presence or absence of nucleotide. LGP2 caps blunt, 3′ or 5′ overhang dsRNA ends with 1 bp longer overall footprint than RIG-I. Structures of 1:1 and 2:1 complexes of *ch*MDA5 with short dsRNA reveal head-to-head packing rather than the polar head-to-tail orientation described for long filaments. *ch*LGP2 and *ch*MDA5 make filaments with a similar axial repeat, although less co-operatively for *ch*LGP2. Overall, LGP2 resembles a chimera combining a MDA5-like helicase domain and RIG-I like CTD supporting both stem and end binding. Functionally, RNA binding is required for LGP2-mediated enhancement of MDA5 activation. We propose that LGP2 end-binding may promote nucleation of MDA5 oligomerization on dsRNA.

INTRODUCTION

Homologous double-stranded RNA (dsRNA) dependent ATPases RIG-I, MDA5, and LGP2 (RIG-I-like helicases [RLHs]) are key cytosolic pattern recognition receptors in the vertebrate innate immune response against RNA viruses. RIG-I senses primarily 5′ppp blunt-end dsRNA (5′ppp-dsRNA), whereas MDA5 is activated by long dsRNA. Consequently, the two sensors respond to different but overlapping sets of viruses (Yoo et al., 2014). Both activated receptors trigger the same downstream signaling pathway, leading to interferon (IFN) induction (Goubau et al., 2013). RIG-I and MDA5 possess tandem N-terminal caspase activation and recruitment domains (CARDs), a central DECH-box helicase domain (Fairman-Williams et al., 2010), and a C-terminal domain (CTD). LGP2 differs in lacking CARDs (Figure 1A) and thus independent signaling activity.

Extensive studies of RIG-I and MDA5 have elucidated their mode of RNA binding and activation and signaling mechanisms (Ahmad and Hur, 2015; Hopfner, 2014). RIG-I binding to short 5′ppp-dsRNA via its CTD and helicase domains releases the CARDs from an auto-inhibitory state, in an ATP-dependent manner (Kowalinski et al., 2011). This allows them to interact with and oligomerize, in a poly-ubiquitin-dependent fashion, downstream signaling partner MAVS (Peisley et al., 2014; Wu et al., 2014). By contrast, MDA5 binds co-operatively to long dsRNA to form protein-coated filaments (Berke et al., 2012; Peisley et al., 2011; Wu et al., 2013); the resulting oligomerization of MDA5 CARDs activates MAVS (Wu et al., 2013).

LGP2 is reported to be both a positive and negative regulator of the anti-viral response (Rodriguez et al., 2014; Zhu et al., 2014). A positive role for LGP2 in anti-viral signaling is supported by the higher susceptibility of LGP2 knockout mice to certain RNA viruses (Satoh et al., 2010) and co-operative activity with MDA5 (Childs et al., 2013). Indeed, small amounts of LGP2 enhance MDA5-mediated signaling (Bruns et al., 2014), and a picornavirus-derived MDA5 agonist was found through its interaction with LGP2 (Deddouche et al., 2014). Furthermore, LGP2, like MDA5, is specifically targeted for inactivation by paramxyovirus V protein (Childs et al., 2012; Rodriguez and Horvath, 2013). A negative role for LGP2 emerged from inhibitory activity observed upon overexpression of LGP2 (Bruns et al., 2013; Liniger et al., 2012, and references therein).

To gain further insight into the role of LGP2 in the anti-viral response and its co-operative role in MDA5 signaling, we performed structural, biochemical, and cell-based studies on chicken (*ch*) LGP2 and MDA5. Interestingly, chicken (Barber et al., 2010) and another *Galliforme*, turkey (according to its draft genome), both lack a RIG-I gene, unlike other vertebrates,

 © 2016 The Author(s). Published by Elsevier Inc.
This is an open access article under the CC BY license (http://creativecommons.org/licenses/by/4.0/).

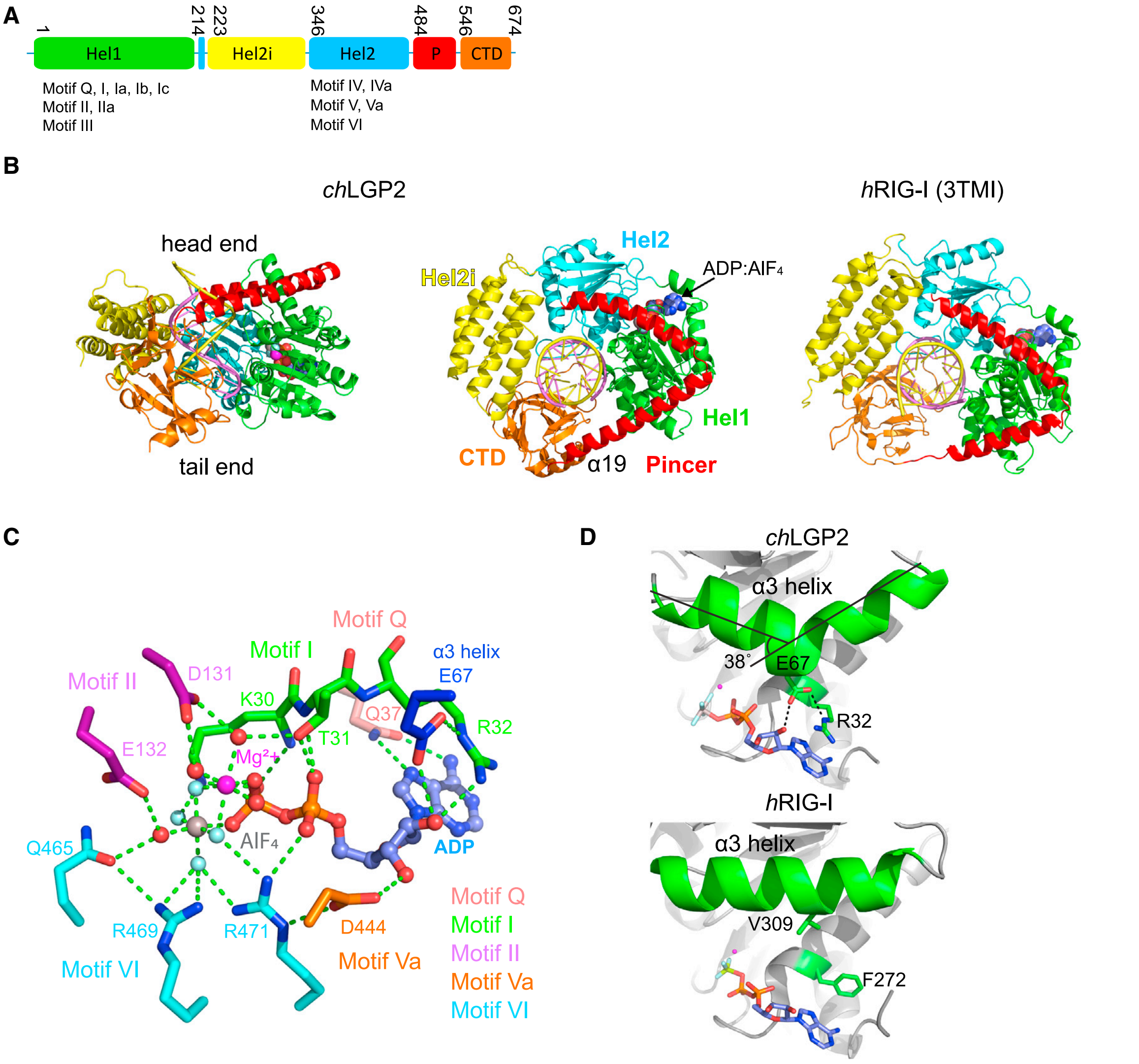

E

Protein	*RNA* 5′ monophosphate	*Km* mM	*kcat* min⁻¹	*kcat/Km* min⁻¹ mM⁻¹
*ch*LGP2	12dsRNA	4.56 ± 0.72	18.0 ± 1.3	3.95
	24dsRNA	2.31 ± 0.62	27.4 ± 2.76	11.90
*d*RIG-I	12dsRNA	0.881 ± 0.158	72.3 ± 4.07	82.11
	24dsRNA	0.665 ± 0.095	120.5 ± 4.98	181.26
*ch*MDA5	12dsRNA	NA	NA	NA
	24dsRNA	2.20 ± 0.47	24.9 ± 2.25	11.30

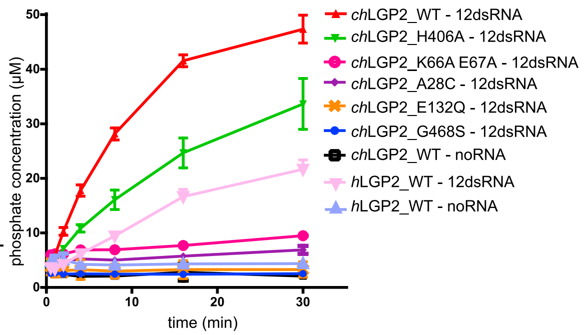

Figure 1. Overall Structure of *ch*LGP2-dsRNA-ADP:AlF_4 Complex

(A) Domain structure of *ch*LGP2. Domain colors are green (Hel1), yellow (Hel2i), cyan (Hel2), red (pincer motif), and orange (CTD).

(B) Side and head-end cartoon view of *ch*LGP2-dsRNA-ADP:AlF_4 complex (left and middle) compared with *h*RIG-I-dsRNA-ADP:BeF_3 complex (PDB: 5E3H [3TMI]) (right). In the side view, the head end contains the pincer motif and the tail end contains the CTD. Domain colors are as in (A), with the zinc atom in the CTD a black sphere. The dsRNA 3′ and 5′ strands are, respectively, violet and yellow. The ADP:AlF_4 is in spheres representation. Note that the second pincer domain helix (α19) of *ch*LGP2 extends right up to the CTD, unlike in *h*RIG-I.

(legend continued on next page)

including many birds (Chen et al., 2013). Nevertheless, chicken cells express MDA5, LGP2, and MAVS (Karpala et al., 2011; Liniger et al., 2012) and produce type I IFN in response to highly pathogenic avian influenza virus, most likely via *ch*MDA5 in co-operation with *ch*LGP2 (Hayashi et al., 2014; Liniger et al., 2012). We present the crystal structure of *ch*LGP2 bound to 5′ mono-phosphate (5′p), 5′ tri-phosphate (5′ppp), and 3′ overhang dsRNA at, respectively, 1.5, 2.2, and 2.0 Å resolution and characterize the RNA binding and ATPase activity of *ch*LGP2 and human (*h*) LGP2. We also report crystal structures of 1:1 and 2:1 CARD-deleted *ch*MDA5-dsRNA complexes at, respectively, 2.60 and 2.75 Å resolution. We demonstrate by electron microscopy (EM) that both *ch*MDA5 and *ch*LGP2 make filaments with dsRNA with the same axial repeat. Finally, functional studies reveal that LGP2 enhanced poly(I:C)-dependent MDA5 signaling, in both chicken and human cells, requires an intact RNA binding site on both the LGP2 helicase and CTD domains.

RESULTS

Overall Structure of *ch*LGP2

We determined three co-crystal structures of full-length *ch*LGP2 with the ATP transition state analog adenosine 5′-diphosphate: aluminum fluoride (ADP:AlF_4) and either a 10-mer palindromic 5′p dsRNA (*ch*LGP2_10p) or 5′ppp dsRNA (*ch*LGP2_10ppp) or a 5′ppp and 3′ two nucleotide (GG) overhang hairpin RNA duplex (*ch*LGP2-3ovg) at, respectively, 1.5, 2.2, and 2.0 Å resolution. A fourth structure of *ch*LGP2 bound to a 12-mer palindromic dsRNA has no bound nucleotide. See Table 1 for crystallographic details.

Overall, *ch*LGP2 resembles other dsRNA-bound RLHs with the two RecA-like helicase domains (Hel1, Hel2), helicase insertion domain (Hel2i), pincer domain (P), and CTD wrapping around the dsRNA stem. Hel1 contains the conserved helicase motifs Q, I, Ia, Ib, Ic, II, IIa, and III, whereas Hel2 contains motifs IV, IVa, V, Va, and VI (Figures 1A and 1B; see also Figure S1 for the secondary structure of *ch*LGP2 and sequence alignment with other RLHs). The unusually high resolution of the structures reveals details of the ADP:AlF_4 binding site and the highly hydrated *ch*LGP2-dsRNA interface. One distinctive feature of LGP2 is that the second pincer domain helix (α19) has eight turns and connects directly to the CTD, whereas in RIG-I, α19 has only six turns, followed by an extended, proline-rich connecting peptide (Figure 1B). Thus in LGP2, the CTD-pincer linkage appears to be more constrained and lacking the functionally important flexibility observed in RNA free RIG-I (Kowalinski et al., 2011). The structure of *ch*LGP2-dsRNA-ADP:AlF_4 complex is in a highly ordered, closed conformation, mimicking the transition state for ATP hydrolysis, and thus most closely resembles the duck RIG-IΔCTD-dsRNA-ADP:AlF_4 complex (*d*RIG-I; Protein Data Bank [PDB]: 4A36) (Kowalinski et al., 2011) in the disposition of the domains, except that the CTD is present as well. In the *ch*LGP2 structure without nucleotide, the helicase is in a semi-closed state with Hel2 separated from Hel1 and partially disordered. See the Discussion for an analysis of the different nucleotide-dependent conformations observed for RLHs.

ADP:AlF_4 Binding and ATP Hydrolysis Activity of *ch*LGP2

ADP:AlF_4 is tightly bound at the interface between the *ch*LGP2 Hel1 and Hel2 with conserved motifs Q, I, and II from Hel1 and motifs Va and VI from Hel2 engaged in the interaction (Figures 1C and S2A). The adenosine base is partially stacked between His4 (motif Q) and LGP2 conserved Arg32 (motif I), and base-specific interactions are provided by the main-chain carbonyl of Glu2 and side chain of Gln7 (motif Q). This differs from RIG-I, where the base stacks between motif Q Arg244 (*h*RIG-I) and motif I Phe272 (equivalent to His4 and Arg32 in LGP2, respectively). The ADP ribose hydrogen bonds with conserved Glu67, which itself is stabilized by a salt bridge with Arg32 (Figures 1C and 1D). Glu67 emerges from a kink in helix α3 (Figure 1D), a feature that is conserved in MDA5 (see below; Figure S1), but not RIG-I, where the equivalent helix is straight and not involved in nucleotide binding (Figure 1D). Motif I (25-PTGAGKTR-32) wraps around the α- and β-phosphates of the ADP, making several hydrogen bonds. Motif Va (440-EEGLD-444) and motif VI (467-QGRARA-472) directly interact with ADP:AlF_4, a characteristic of the closed form. Motif Va Asp444 interacts with the ADP ribose and stabilizes Arg471 of motif VI. Arg471 interacts with the α-phosphate and two fluorines of AlF_4. Arg469, stabilized by Gln465, interacts with a third fluorine, mimicking stabilization of the transition state (Figures 1C and S2B). The high-resolution structure reveals the complex hydrogen bonding network involving Asp131 and Glu132 (motif II), AlF_4, Mg^{2+}, and water molecules in the presumed transition state. The octahedrally coordinated Mg^{2+} ion ligates one oxygen atom of the β-phosphate, two fluorine atoms, and three water molecules. Two magnesium coordinated water molecules form direct contacts with the side chains of Thr31 (motif I) and Asp131 (motif II) (Figures 1C and S2B). One water molecule co-ordinated by Glu132 (motif II, DECH) and Gln465 (motif VI) likely represents the attacking nucleophilic water, which catalyzes ATP hydrolysis (Figures 1C and S2B).

According to Bruns et al. (2013), *h*LGP2 has significant basal (dsRNA-independent) ATPase activity. We find that both *ch*LGP2 and *h*LGP2 ATPase activity is strictly dsRNA dependent (Figure 1E). Unlike MDA5, the activity is not dsRNA length dependent (Figures S2C and S2D). Moreover the ATP catalytic

(C) Details of the immediate protein ligands of the ADP:AlF_4:Mg^{2+} bound in *ch*LGP2, all of which (except Glu67) come from helicase motifs Q, I and II (Hel1), and Va and VI (Hel2), which are colored as indicated. The Mg^{2+} ion, aluminum, and fluorine atoms are, respectively, purple, gray, and light blue spheres. Glu132 and Gln465 coordinate the mimic of the attacking water molecule (red sphere) in this transition-state analog complex.

(D) Diagram showing how in *ch*LGP2, Glu67 emerges from helix *a*3 to be involved in ATP ribose binding and stabilization of Arg32, which stacks on the adenine base (top). A very similar situation occurs in MDA5 (see Figure 4D) but not RIG-I (bottom).

(E) Left: table of K_m and *k*cat values for the RNA-dependent ATPase activity of wild-type *ch*LGP2, *d*RIG-I, and *ch*MDA5. Right: representative ATP hydrolysis curves for *ch*LGP2 and *h*LGP2 with without dsRNA and various mutations of *ch*LGP2. See also Figure S2.

See also Figures S1–S3.

Table 1. Diffraction Data and Refinement Statistics for *ch*LGP2

Crystal	*ch*LGP2 5′ppp 10-mer dsRNA ADP:AlF_4:Mg^{2+}	*ch*LGP2 5′p 10-mer dsRNA ADP:AlF_4:Mg^{2+}	*ch*LGP2 5′ppp 10-mer 3′ ovg dsRNA ADP:AlF_4:Mg^{2+}	*ch*LGP2 5′p 12-mer dsRNA
Diffraction data				
Space group	$P2_12_12_1$	$P2_12_12_1$	$P2_12_12_1$	$P6_4$
Cell dimensions (Å)	a = 70.14, b = 96.58	a = 69.59, b = 97.05	a = 69.86, b = 97.43	a = b = 90.09,
	c = 122.86	c = 122.52	c = 122.58	c = 196.73
	$\alpha = \beta = \gamma = 90$	$\alpha = \beta = \gamma = 90$	$\alpha = \beta = \gamma = 90$	$\alpha = \beta = 90$, $\gamma = 120.00$
Wavelength (Å)	0.8726	0.9786	1.072	0.9786
Resolution range of data (last shell) (Å)	50.0–2.2 (2.28–2.2)	50.0–1.50 (1.55–1.50)	50.0–2.0 (2.10–2.0)	45.04–3.60 (3.73–3.60)
Completeness (last shell) (%)	99.9 (99.4)	99.9 (99.8)	99.9 (98.7)	99.8 (99.0)
R-sym (last shell) (%)	16.9 (83.5)	4.8 (74.6)	25.9 (79.5)	6.7 (118.0)
I/σI (last shell)	8.73 (1.87)	17.6 (2.31)	6.31 (1.38)	20.74 (2.01)
Redundancy (last shell)	6.64 (6.50)	5.25 (5.27)	4.47 (4.35)	11.51 (10.32)
Refinement				
Reflections used in refinement work (free)	40,943 (2,114)	126,226 (6,681)	53,847 (2,814)	10,121 (542)
R-work (last shell)	0.188 (0.267)	0.133 (0.244)	0.218 (0.333)	0.253 (0.359)
R-free (last shell)	0.245 (0.294)	0.168 (0.292)	0.255 (0.317)	0.319 (0.346)
Number of non-hydrogen atoms	6,458	6,638	6,188	5,540
Protein	5,616	5,531	5,356	5,028
RNA	464	495	506	512
Ligand (nucleotide)	33	33	33	–
Solvent	345	579	293	–
Geometry and B factors				
Rms (bonds)	0.009	0.013	0.008	0.006
Rms (angles)	1.365	1.510	1.278	0.954
Ramachandran favored (%)	97.1	98.4	97.5	94
Ramachandran outliers (%)	0.72	0.15	0.15	0.32
Clash score	1.59	3.70	1.93	1.11
Average B factor	34.0	29.9	39.2	202.8
Protein	34.3	29.5	39.4	205.9
RNA	30.8	22.8	36.7	173.1
Ligand (nucleotide)	20.4	22.9	30.4	–
Solvent	33.6	40.7	38.7	–

Rms, root-mean-square.

efficiency, as indicated by *k*cat/*K*m, is much lower for both *ch*LGP2 and *ch*MDA5 than for *d*RIG-I (Figures 1E and S2C). The importance of the interaction of Glu67 with the nucleotide ribose is underlined by the abolished ATPase activity of a *ch*LGP2 K66A/E67A mutant (Figure 1E). Within the highly conserved motif I sequence (25-PTGAGKTR-32, *ch*LGP2) the fourth position differs, being C/S in RIG-I, S in MDA5, and A/G/S in LGP2 (Figure S2E), and an A28C mutation abolishes the ATPase activity of *ch*LGP2 (Figure 1E). Glu132 (motif II) (Civril et al., 2011; Louber et al., 2015) and Gly468 (motif VI; Figure S2F) (Funabiki et al., 2014) are absolutely conserved and crucial for ATP hydrolysis by RIG-I and MDA5, correlating with their mutation underlying genetic disease (Funabiki et al., 2014; Jang et al., 2015). Accordingly, E132Q or G468S mutations abolish the ATPase activity of *ch*LGP2 (Figure 1E).

Recognition of dsRNA by *ch*LGP2

Because of the full closure of all domains around the dsRNA (Figure 2A), many residues of *ch*LGP2 make direct interactions to the dsRNA. These are detailed in Figure 2B, in which numerous water-mediated protein-RNA or direct water-RNA interactions are omitted for clarity. The protein-RNA interface buries a total solvent accessible surface of 4,700 $Å^2$ (10p) or 4,786 $Å^2$ (10ppp), as compared with 3,109 $Å^2$ for *h*RIG-I in the semi-closed state (PDB: 5E3H [3TMI]). RNA binding residues come from the CTD and the conserved motifs Ia, Ib, Ic, and IIa from Hel1 and IV, IVa, and V from Hel2. In addition, Hel2i and the pincer domain interact with the dsRNA. Hel2i residues from helix α10 (Gln256, Gln260, Arg261, Glu264, and Asn267) as well as Arg285 from helix α11 interact with both dsRNA strands via the minor groove. Arg486 and Arg490

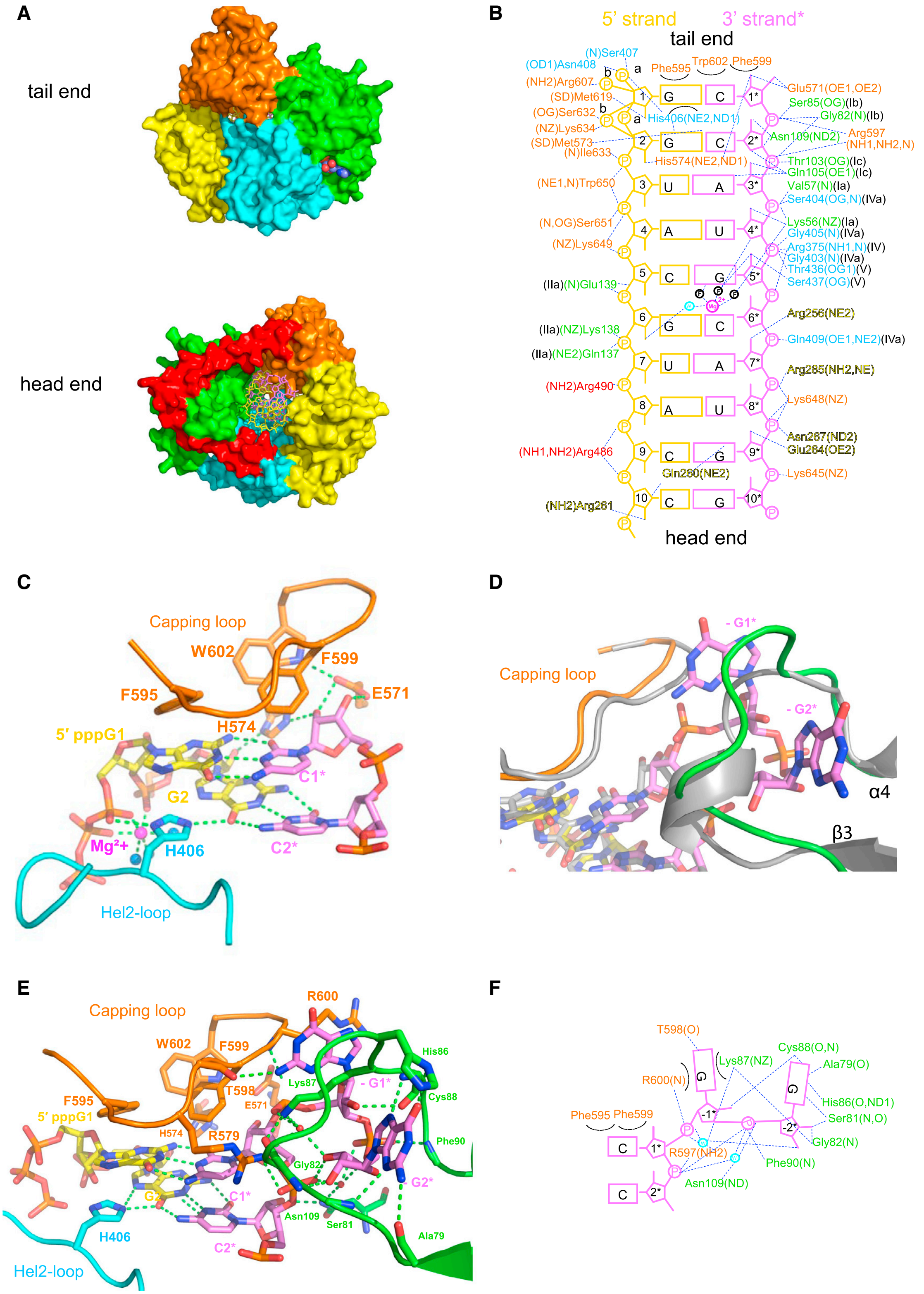

Figure 2. dsRNA Binding by *ch*LGP2

(A) Surface representation of the *ch*GP2-dsRNA-ADP:AlF_4 complex viewed from the tail (left) and head (right) ends, colored as in Figure 1. At the tail end, the protein completely caps the blunt end of the dsRNA, whereas at the head end, the dsRNA can be extended.

(legend continued on next page)

from the long helix α18 of the pincer domain also interact directly with the phosphate backbone of the dsRNA 5′ strand. The above-cited interacting residues are conserved or conservatively substituted polar residues in LGP2 from different organisms (Figure S3).

Within the full-length *ch*LGP2 structure, the CTD interacts with the dsRNA extremity in a similar fashion to previously reported for the isolated *h*LGP2 CTD (PDB: 3EQT) (Li et al., 2009; Pippig et al., 2009), with the terminal base pair (5′-G1:C1*-3′) making extensive hydrophobic interactions with aromatic residues Phe595, Phe599, and Trp602 from the 591–602 "capping loop" between strands β18 and β19 (Figure 2C). This loop also makes significant contacts with Hel1 in the vicinity of motif Ib, thereby reinforcing the closure of LGP2 over the RNA. The 3′ end interacts mainly with the turn between strands β16 and β17 (the 571–574 "3′ end-binding loop"; Figure S1), with fully conserved Glu571 hydrogen bonding with both hydroxyls of C1* and His574 bridging between the C1* and G2 riboses (Figure 2C). These interactions would appear to block extension of the 3′ strand of the dsRNA (but see below). The 5′ end interacts with both the CTD and the extended 402–413 "Hel2-loop" (Figure S1), which emerges between strand β10 and helix α16 and crosses the major groove of the dsRNA (Figure 2C). In *ch*LGP2, the G1 base is imperfectly stacked between His406 and tilted Phe595, whereas the equivalent residues in *h*LGP2 are aliphatic, Asn408 and Ile597, respectively. Interestingly, an aromatic residue (His or Tyr) is found at position 406 in the Hel2-loop only in bird, reptile, and amphibian LGP2s (i.e., not in mammals or fish, in which it is usually an Asn), whereas only birds (with a few exceptions) and frogs have an aromatic residue at position 595 (Figure S3). The deletion of the Hel2-loop significantly affects ATP hydrolysis by *h*MDA5 (Wu et al., 2013). We found that the H406A mutation in *ch*LGP2 also reduced ATP hydrolysis but did not significantly change the affinity to RNA (Figures 1E and 3B). The CTD of LGP2 contains a lysine-rich motif (644-KKKYKKWS-651), with highly conserved Lys648 and Lys649 contacting phosphates of both strands across the major groove (respectively A7*, U8*, and C5) and conserved Trp650 to U3 and A4 phosphates (Figure 2B). The K648/K649E double mutation reduces *ch*LGP2 affinity to dsRNA 56 times compared with wild-type (WT). A quadruple mutant with the additional glutamate substitutions of the conserved helicase residues Lys138 and Arg490 that interact with consecutive 5′ strand phosphates (Figure 2B) has ~1,500 times less affinity, while the helicase mutations alone reduce the affinity by only 2.7 times (Figure 3B). This confirms the strong direct binding of the CTD to the RNA binding compared to the helicase domain and reveals the cooperativity of dsRNA binding to the two domains.

*ch*LGP2 Binds One Extra Base Pair of dsRNA Compared with RIG-I

An intriguing observation is that the blunt end of the dsRNA is located 1 bp deeper into the *ch*LGP2 CTD than occurs in RIG-I (Figure 3A). This is not because the entire CTD is raised up but because the capping loop of LGP2 is flat, whereas that of RIG-I bends downward placing the side chain of Phe853 at the same level as the blunt-end base pair in LGP2 (Figure 3A). This shifts all the canonical contacts between helicase motifs and backbone phosphates 1 bp down from the blunt end in LGP2 compared with RIG-I, with motifs Ib and Ic contacting phosphate 1 and phosphate 2 of the 3′ strand in RIG-I and LGP2, respectively (Figure 3A). Consequently, the overall footprint of LGP2 on dsRNA is 10 bp for LGP2 rather than 9 bp for RIG-I, consistent with the larger surface area of protein-RNA contact for LGP2.

*ch*LGP2 Binds to dsRNA with Modified 5′ and 3′ Ends

To further characterize RNA binding to *ch*LGP2, we determined structures with the 5′ppp form the same 10-mer dsRNA (i.e., the cognate RIG-I ligand) and a 5′ppp hairpin dsRNA with 3′ GG overhang at 2.2 Å (*ch*LGP2-10ppp) and 2.0 Å (*ch*LGP2-3′ovg) resolution, respectively (Table 1). Note that the hairpin stem has some base pair differences from the 10-mer dsRNA beyond the second base pair (Figure S4). We also measured the binding affinity of *ch*LGP2 to different RNAs with and without various nucleotides and compared the results with *h*RIG-I (Figure 3B).

In the *ch*LGP2-10ppp structure, the 5′ end has a unique conformation (corresponding to one of the *ch*LGP2-10p conformations), with the 5′ppp being accommodated without structural changes. The α- and β-phosphates interact with His406, Ser407, and Asn408 from the Hel2-loop, and the β- and γ-phosphates interact with Lys634 (Figure S4A). An octahedrally coordinated magnesium ion directly interacts with the α- and β-phosphates as well as the phosphate of G2 (Figure 2C). The 5′ppp conformation in the *ch*LGP2-3′ovg structure is different and resembles the other 5′ end conformation observed in the *ch*LGP2-10p structure. The tri-phosphate makes alternative interactions with Asn408, Arg607, and Lys634, and there is no bound magnesium (Figure S4B).

The *ch*LGP2-3ovg structure is overall similar to the previous structures, the major difference being a shift of the first G1-C1* base pair toward Hel1, a slight shift in the opposite direction of the CTD (including the capping and 3′ end-binding loops), and a

(B) Schematic diagram showing the interactions of *ch*LGP2 residues with the 10-mer dsRNA. Residues are colored according to domain and labeled with the conserved motif they belong to and the atom involved in the interaction. Polar interactions are indicated with a blue dotted line (cutoff 3.5 Å) and hydrophobic interactions by an arc. Numerous direct or water-mediated interactions are omitted for clarity. The 3′ strand nucleotides are numbered with an asterisk (i.e., 5′-G1:C1*-3′ is the first base pair from the tail end). The two observed alternative conformations of the first and second 5′ phosphates are shown.

(C) Details of the *ch*LGP2-RNA interactions that cap the blunt end of the 5′ppp dsRNA showing the role of aromatic residues Phe595, Phe599, and Trp602 from the capping loop, Glu571 and His574 from the 3′ end-binding loop, and His406 from the Hel2-loop. The Mg^{2+} coordinated by the 5′ppp is a magenta sphere.

(D) Comparison of *ch*LGP2 structures with 5′ppp-dsRNA without (gray) or with (colors) a 3′-GG overhang, showing how a slight displacement of the capping loop and rearrangement of the β3-α4 loop accommodates the 3′ end extrusion.

(E) Schematic diagram showing interactions with the 3′-GG overhang nucleotides (denoted G-1* and G-2*), annotated as in (B).

(F) Structural details of the network of interactions between *ch*LGP2 and the 3′-GG overhang nucleotides. Compared with (C), Glu571 and His574 no longer interact with C1* ribose.

See also Figures S4 and S5.

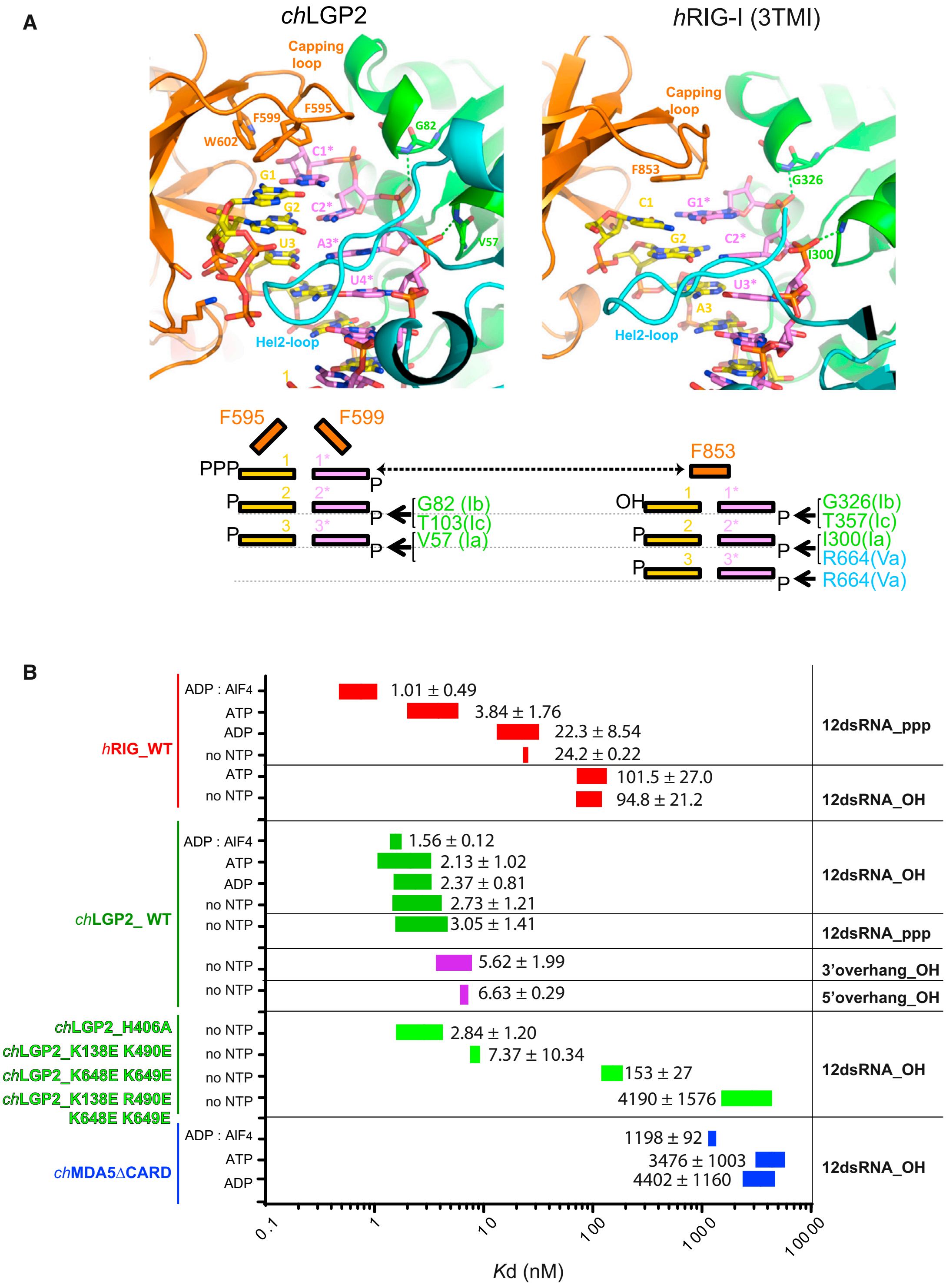

Figure 3. Comparison of *ch*LGP2 and *h*RIG-I Binding to dsRNA

(A) Structural and schematic diagrams comparing the mode of end binding of *ch*LGP2 and *h*RIG-I illustrating the extra base pair sequestered by *ch*LGP2, which is at the same level as Phe853 from the capping loop of RIG-I. Conserved interactions with motifs Ia, Ib, and Ic are shown.

(B) K_d values between *ch*LGP2 (green), full-length *h*RIG-I (red), and *ch*MDA5 (blue) and a 12-mer dsRNA with different 5′ modifications were measured without nucleotide (no NTP) and with various nucleotides (ADP-AlF_4, ADP, ATP). The values shown correspond to K_d (nM) ± SD on the basis of the fluorescence anisotropy binding curves shown in Figure S5B.

See also Figures S4 and S5.

CellPress

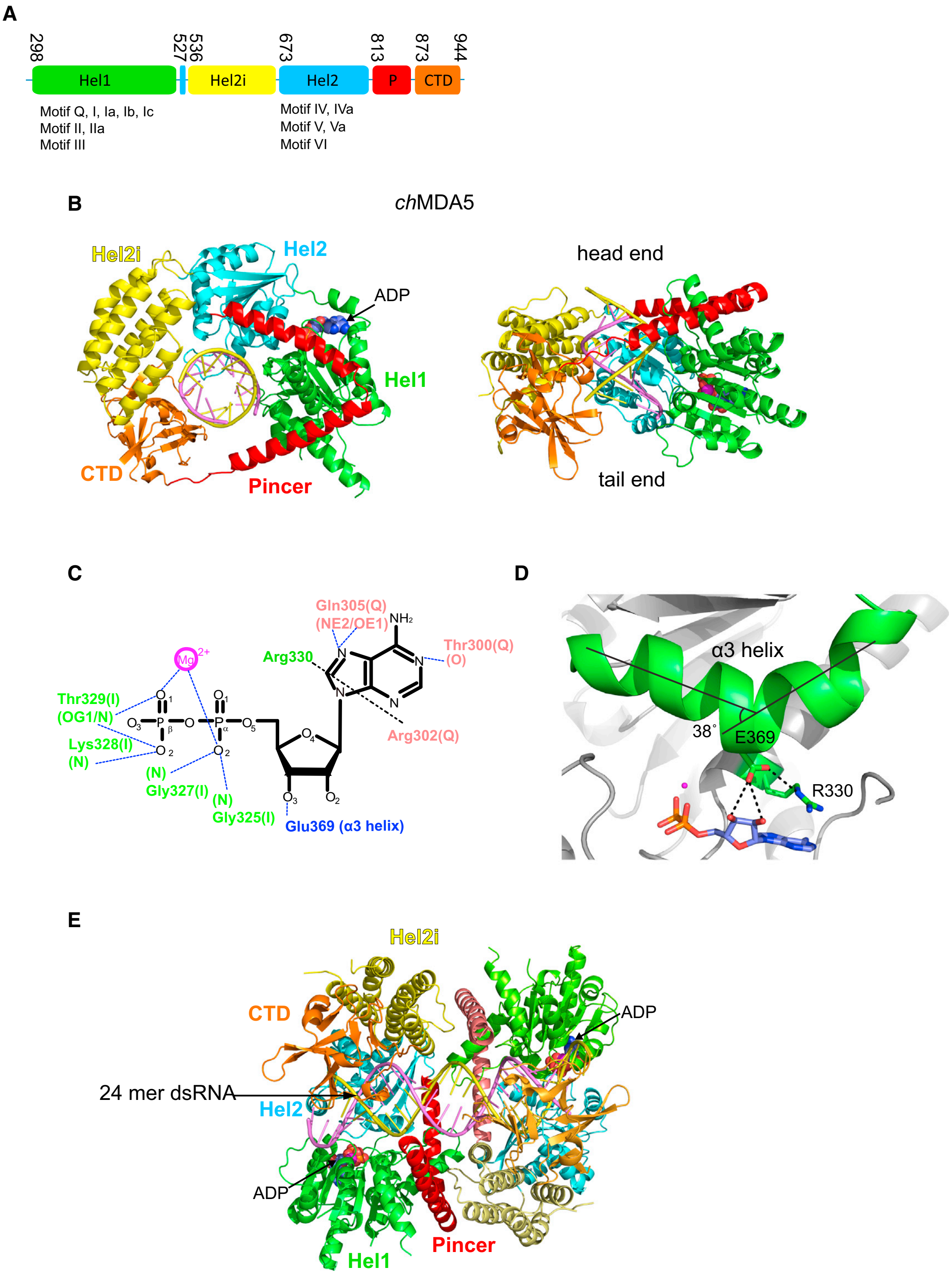

Figure 4. Overall Structure of *ch*MDA5ΔCARD-ADP Complex

(A) Domain structure of *ch*MDA5ΔCARD, omitting the N-terminal tandem CARD domains.

(B) Ribbon diagram of the *ch*MDA5ΔCARD-10-mer dsRNA-ADP complex from head end along (left) and perpendicular (right) to the dsRNA axis, colored according to Figure 2A.

(C) Diagram showing the interactions of *ch*MDA5 with ADP:Mg.

(legend continued on next page)

significant rearrangement of the β3-α4 loop (residues 79–90), which would otherwise clash with the additional bases (Figure 2D). The net result is to disengage Glu571 and His574 from the ribose of C1 allowing 3′ extension of the RNA by extrusion of overhang nucleotide G-1* between the capping loop and the refolded β3-α4 loop. The G-1* base is stacked between the side chains of Arg600 and Lys87, and the resulting conformation of the β3-α4 loop permits G-1* and G-2* to make numerous interactions with residues 79–90, including a number of base-specific interactions (Figures 2E and 2F). This together with the burial of the G-2* ribose, whose 3′ OH makes a hydrogen bond to the phosphate of C1*, suggests that the observed structure may be specific to this particular 3′-GG overhang but does show how relatively minor structural perturbations allow extrusion of a 3′ overhang, while extension of the double helical stem is prevented by maintenance of the stacking of the capping loop over the first base pair.

The affinities of LGP2 to various dsRNA ligands were determined by fluorescence anisotropy (Figure 3B). *ch*LGP2 binds to a blunt-end 12-mer dsRNA with an affinity independent of whether the 5′ is a hydroxyl or a tri-phosphate (Figures 3B and S5B), consistent with the lack of significant additional interactions to the tri-phosphate (and its variable conformation) observed in the crystal structures. By comparison, *h*RIG-I, as expected, binds to 5′ppp more tightly than to 5′-OH dsRNA, a result of the interactions of the 5′ppp with three lysines and a histidine, all strictly conserved (Luo et al., 2012). In addition, while *h*RIG-I's affinity to 5′ppp dsRNA depends on which nucleotide is also bound, being highest for ADP:AlF_4 ($K_d \sim 1$ nM) and 20 times weaker with ADP or no nucleotide (Figure 3B), the affinity of *ch*LGP2 for dsRNA is independent of bound nucleotide (Figure 3B). Finally, consistent with the 3′-ovg structure, the affinity of *ch*LGP2 for 3′ and 5′ overhangs is only marginally reduced by a factor of two. In comparison, *h*RIG-I can also bind 3′ overhangs with high affinity (provided there is a 5′ppp), but 5′ overhangs are far less well accepted (Ramanathan et al., 2016).

Overall Structure of *ch*MDA5

In the absence of endogenous RIG-I, it is thought that *ch*MDA5, perhaps in co-operation with *ch*LGP2, is responsible for the RNA dependent anti-viral response to viruses that are usually detected by RIG-I (Liniger et al., 2012). We therefore determined the crystal structure of *ch*MDA5 (see Figure 4A for the domain structure) and investigated its ability, as well as that of *ch*LGP2, to form filaments with dsRNA.

For co-crystallization, we used 5′p palindromic duplex RNAs, the ATP analog AMPPNP, and a construct denoted *ch*MDA5Δ CARD-Q, comprising the helicase and CTD (residues 298–994) with the mutation E436Q and lacking seven residues at the C terminus. The E436Q mutant in conserved motif II (i.e., DECH becomes DQCH) virtually abolishes ATPase activity (Louber et al., 2015) and for another DEAD-box helicase, VASA, permitted trapping of the helicase substrate RNA (Xiol et al., 2014). Unlike for the *h*MDA5 crystal structure (Wu et al., 2013) the long, acidic loop between helixes α12 and α13 of Hel2i, which is 17 residues shorter in *ch*MDA5, was not deleted (Figure S1). The structure of a 1:1 complex of *ch*MDA5ΔCARD-Q with ADP:Mg^{2+} and 10 bp dsRNA (*ch*MDA5-10) and a 2:1 complex with 24 bp dsRNA (*ch*MDA5-24) were solved by molecular replacement at 2.60 and 2.75 Å, respectively (Table 2), as well as a 2:1 complex with 27 bp dsRNA at 7.2 Å resolution.

Overall, the *ch*MDA5-dsRNA structures resemble that of *h*MDA5 bound to a 12-mer dsRNA (PDB: 4GL2; root-mean-square deviation of 1.16 Å for all Cαs) (Figure 4) but with considerably higher resolution (*h*MDA5 is at 3.56 Å), and the model is more complete. Interestingly, the *h*MDA5, *ch*MDA5-10 (Figure 4B), *ch*MDA5-24 (Figure 4E), and *ch*MDA5-27 structures all capture the dsRNAs bound in different longitudinal positions. The *ch*MDA5-24 and *ch*MDA5-27 structures reveal two different 2:1 complexes with two MDA5 molecules bound to the same dsRNA. Following Wu et al. (2013), looking perpendicular to the RNA axis, we denote the head end of MDA5 as the pincer domain containing face and the tail end as that containing the CTD (see Figures 4B and S1 for the secondary structure of *ch*MDA5 and sequence alignments).

*ch*MDA5 Bound to dsRNA and ADP:Mg^{2+} Is in the Semi-closed Conformation

In all *ch*MDA5-dsRNA structures, the helicase is in the semi-closed conformation (Figures 4B and S6; Table S1), but unlike the semi-closed forms of RIG-I and LGP2, Hel2 is well ordered. In the two higher resolution structures, the electron density in the nucleotide binding site clearly corresponds to ADP:Mg^{2+}, presumably because of slow AMPPNP hydrolysis. The ADP:Mg^{2+} is bound in canonical fashion by motifs Q and I, with the adenosine stacked between Arg330 (motif I) and Arg302 (motif Q) (Figure 4C). This arrangement is more similar to that in *ch*LGP2 than RIG-I, with helix α3 being kinked toward the nucleotide binding cleft in the same way, allowing Glu369, stabilized by Arg330, to hydrogen-bond to the 3′ OH of ADP (corresponding to Glu67 and Arg32 in *ch*LGP2; compare Figures 1D and 4D).

*ch*MDA5 Interaction with dsRNA

MDA5 binds to the dsRNA stem using the canonical helicase motifs Ia, Ib, Ic, IIa, IV, IVa, and V as well as conserved glutamines Gln568 and Gln572 from α10 of Hel2i, in a similar manner to other RLHs (Figure 5). The distinguishing feature of MDA5 (Wu et al., 2013) is that its CTD interacts intimately with Hel2i and is displaced relative to the position in LGP2 or RIG-I to allow a dsRNA helix to pass through the molecule. Thus, the domain arrangement in MDA5, at the tail end, resembles an open horseshoe rather than a closed circle (compare Figures 5A and 2A). The Hel2-loop (729–740 in *ch*MDA5, notably residues His733, Asn734, and Lys738; Figure S1), senses the major groove of the dsRNA, as in LGP2 and RIG-I, but without making any specific interactions (Figure 5C). The CTD 3′ end-binding loop (898-ENMH-901 in *ch*MDA5) contacts the backbone of the 5′ strand,

(D) Diagram showing how Glu369 emerges from helix a3 to be involved in ribose binding and stabilization of Arg330, which stacks on the adenine base. A very similar situation occurs in LGP2 but not RIG-I (see Figure 1D).

(E) Ribbon diagram of the head-to-head *ch*MDA5ΔCARD-24-mer dsRNA-ADP complex perpendicular to the dsRNA axis. Domains and dsRNA are colored according to Figure 2A, with lighter colors for the second monomer.

Table 2. Diffraction Data and Refinement Statistics for *ch*MDA5

Crystal	chMDA5 5′p 10-mer dsRNA ADP-Mg^{2+} Twinned	chMDA5 5′p 10-mer dsRNA ADP-Mg^{2+}	chMDA5 5′p 10-mer dsRNA ADP-Mg^{2+} Untwinned	chMDA5 5′p 24-mer dsRNA ADP-Mg^{2+}
Diffraction data				
Space group	$P2_1$	$P2_12_12_1$	$P2_1$	$P2_12_12_1$
Cell dimensions (Å)	a = 70.16, b = 138.70, c = 100.42	a = 101.92, b = 132.47, c = 139.04	a = 72.08, b = 139.73, c = 103.19	a = 99.75, b = 133.40, c = 138.44
	$\alpha = \gamma = 90$, $\beta = 109.48$	$\alpha = \beta = \gamma = 90$	$\alpha = \gamma = 90$, $\beta = 110.142$	$\alpha = \beta = \gamma = 90$
Wavelength (Å)	0.9724	0.9763	0.9763	0.9724
Resolution range of data (last shell) (Å)	50.0–2.60 (2.69–2.60)	50.0–2.60 (2.69–2.60)	48.6–2.95 (3.06–2.95)	50.0–2.75 (2.82–2.75)
Completeness (last shell) (%)	99.3 (99.6)	98.2 (99.2)	94.0 (96.6)	99.1 (99.2)
R-sym (last shell) (%)	9.60 (89.6)	9.60 (99.2)	9.4 (87.3)	16.8 (80.9)
I/σI (last shell)	9.83 (1.73)	10.31 (1.87)	9.94 (1.78)	6.58 (1.52)
Redundancy (last shell)	3.52 (3.63)	5.06 (5.21)	5.20 (5.05)	4.66 (4.89)
Refinement				
Reflections used in refinement work (free)	52,489 (2,811)	54,624 (2,901)	36,153 (1,907)	45,840 (2,433)
R-work (last shell)	0.271 (0.273)	0.277 (0.401)	0.256 (0.423)	0.290 (0.487)
R-free (last shell)	0.290 (0.286)	0.312 (0.418)	0.270 (0.421)	0.322 (0.492)
Number of non-hydrogen atoms	11,761	11,870	11,661	11,616
Protein	10,851	10,773	10,751	10,539
RNA	854	873	854	1,021
Ligand (nucleotide)	56	56	56	56
Solvent	–	168	–	–
Geometry				
Rms (bonds)	0.007	0.007	0.008	0.008
Rms (angles)	1.11	1.06	1.28	1.21
Ramachandran favored (%)	96.3	96.0	95.8	93.7
Ramachandran outliers (%)	0.31	0.31	0	0.4
Clash score	1.08	0.39	0.48	1.4
Average B factor	58.1	67.2	81.7	70.0
Protein	59.1	67.2	83.5	71.2
RNA	44.4	56.2	58.6	56.9
Ligand	59.5	65.1	96.1	74.0
Solvent	–	49.1	–	–

Rms, root-mean-square.

without blocking extension of the 3′ end, and the conserved 977–980 turn, toward the end of the CTD, also makes backbone interactions to both strands in the major groove (Figure 5C). Another major distinctive feature of MDA5 is the position and nature of the CTD "capping loop" (918–927 in *ch*MDA5; Figure S1). While in all MDA5 crystal structures with dsRNA this loop is disordered, it is well structured in the isolated *h*MDA5 CTD structure (PDB: 3GA3), revealing it to be more compact than in LGP2 or RIG-I. Moreover, the MDA5 loop, unlike those of LGP2 and RIG-I, lacks the bulky hydrophobic residues that would favor interaction with a blunt end base pair. Modeling of the loop from the superposed *h*MDA5 CTD structure suggests that when binding to a continuous dsRNA the capping loop and N-terminal end of α16 of Hel2 could favorably interact with the minor groove and extended 5′ strand backbone, respectively (Figure 5D).

Dimers of *ch*MDA5 on dsRNA Have the Head-Head Configuration

In the *ch*MDA5 24-mer structure, two MDA5 molecules are stacked head to head on one dsRNA, with a two-fold symmetry axis perpendicular to the dsRNA and between the two molecules (Figures 4E and 6A); additionally there is a 35° bend in the dsRNA axis (Figures 6A and 6B). In the *ch*MDA5 10-mer structure, there are two 1:1 complexes in the asymmetric unit, which are arranged exactly as in the 24-mer structure except that the dsRNA is not continuous, lacking one base pair between the molecules (Figures 6A and 6B). The *h*MDA5 12-mer structure has two 1:1 complexes in the asymmetric unit, but with no pseudo-continuous RNA helix running through multiple MDA5 molecules. Compared with the *ch*MDA5 10-mer structure, the *h*MDA5 12-mer structure has three extra base pairs at the tail end but one fewer at the head

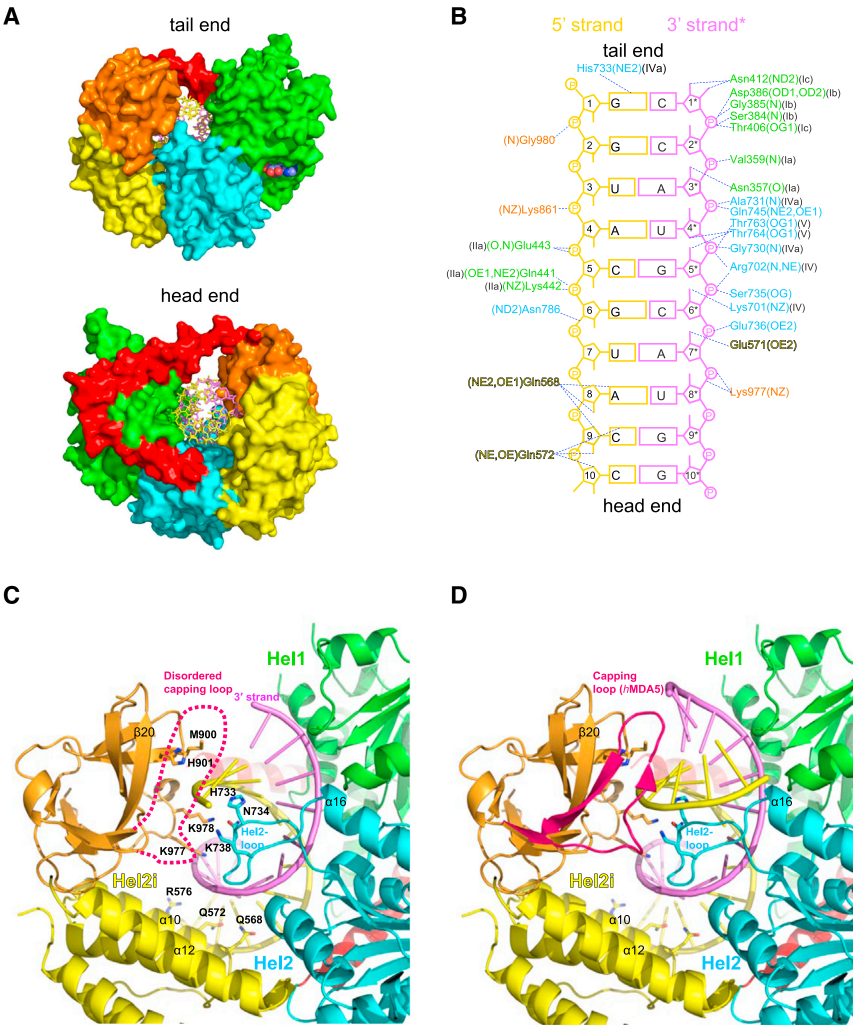

Figure 5. Interactions of *ch*MDA5 with dsRNA

(A) Head and tail views down the dsRNA axis of the chMDA5 structure showing that the dsRNA can continue from both ends (see also D). Compared with LGP2 (Figure 2A), MDA5 appears from the tail end as an open horseshoe rather than a disk.

(B) Schematic diagram showing the interactions of *ch*MDA5 residues with the 10-mer dsRNA, annotated as in Figure 2B.

(C) Ribbon diagram showing protein-RNA interactions in the *ch*MDA5 10-mer structure at different levels along the dsRNA, including those from helix α12 of Hel2i, the Hel2 loop, and two loops of the CTD. The putative position of the disordered MDA5 capping loop is shown dotted on the basis of the crystal structure of the isolated *h*MDA5 CTD. Protein-RNA interactions mediated by the Hel1 and Hel2 domains have been omitted for clarity (see B).

(D) As in (C), but the dsRNA has been extended by 3 bp to emerge from the tail end of the molecule. Modeling suggests that the MDA5 capping loop could be involved in another level of protein-RNA interactions as well as possibly mediating the tail-to-head protein-protein interface.

end (Figures 6A and 6B). By comparison, the *ch*MDA5 24-mer structure gains 1 bp at the head end (making the dsRNA continuous between the two molecules) and 1–2 bp at the tail end (because the 24-mer structure probably corresponds to a superposition of two structures with a shift of 1 bp). More base pairs at the tail end correlate with a slight movement of the CTD toward the dsRNA. The bend in the dsRNA axis in the 1:1 and 2:1 *ch*MDA5 structures allows two MDA5 molecules to pack closely together in head-to-head fashion, with a center-of-mass to center-of-mass distance of 43.1 Å and total buried protein-protein surface of 1,961 and 1,907 Å^2, respectively. The head-to-head interface involves two-fold symmetric interactions between the pincer domain of one molecule and two protruding loops of the other molecule (Figure 6C). The beginning of the pincer domain (812-SGSAVER-817) interacts with the loop preceding Hel1 helix α9 (489-RSNS-492), which protrudes from the surface of the neighboring molecule. The end of the pincer domain (852-LQSI-855) interacts with the loop (562-KSE-564) between helices α11 and α12 of the second molecule. Note that residues V816 and E817 in *ch*MDA5 are equivalent to I841 and E842, implicated in the head-to-tail interaction in *h*MDA5 (Wu et al., 2013).

In the *ch*MDA 27-mer complex, the dsRNA is straight. Moreover, compared with the 24-mer complex, the two MDA5 head-to-head molecules are more distantly separated by 48.7 Å, and their relative rotation differs, with the second molecule of the 27-mer dimer being rotated a further 106° around and translated 10.6 Å further along the dsRNA (Figures 6A and 6B). Only regions 852–855 and 562–564 are close enough to make contact, thus reducing the total buried surface area to 484 Å^2. Thus, whereas in the case of the 24-mer 2:1 complex, stronger protein-protein interactions are able to distort the path of the dsRNA, the 27-mer structure paradoxically suggests that *ch*MDA5 prefers to bind at the end of a short dsRNA, even if this means reducing protein-protein interactions. However, it cannot be ruled out that the observed arrangement is dependent on crystal contacts.

*ch*LGP2 Makes Filaments with dsRNA

To test the ability of *ch*LGP2 to form filaments on continuous dsRNA, complexes with Φ6 2,948, 4,063, and 6,374 bp long dsRNA were studied by EM. When mixed with a protein/RNA ratio of 0.2:1 in the presence of 2 mM ATP and imaged using negative stain, *ch*LGP2 forms short and discrete multimeric clusters on Φ6 dsRNA (Figure 6D). At a ratio of 1:1, corresponding to one protein per 15 bp, as in *h*MDA5 polar filaments (Berke et al., 2012; Wu et al., 2013), more extensive and regular dsRNA coating interspersed with naked dsRNA is observed (Figure 6D). The presence of ATP or ADP:AlF_4 favors the formation of filamentous regions. In comparison, at a 1:1 ratio, full-length *ch*MDA5 completely coats dsRNA, independently of bound

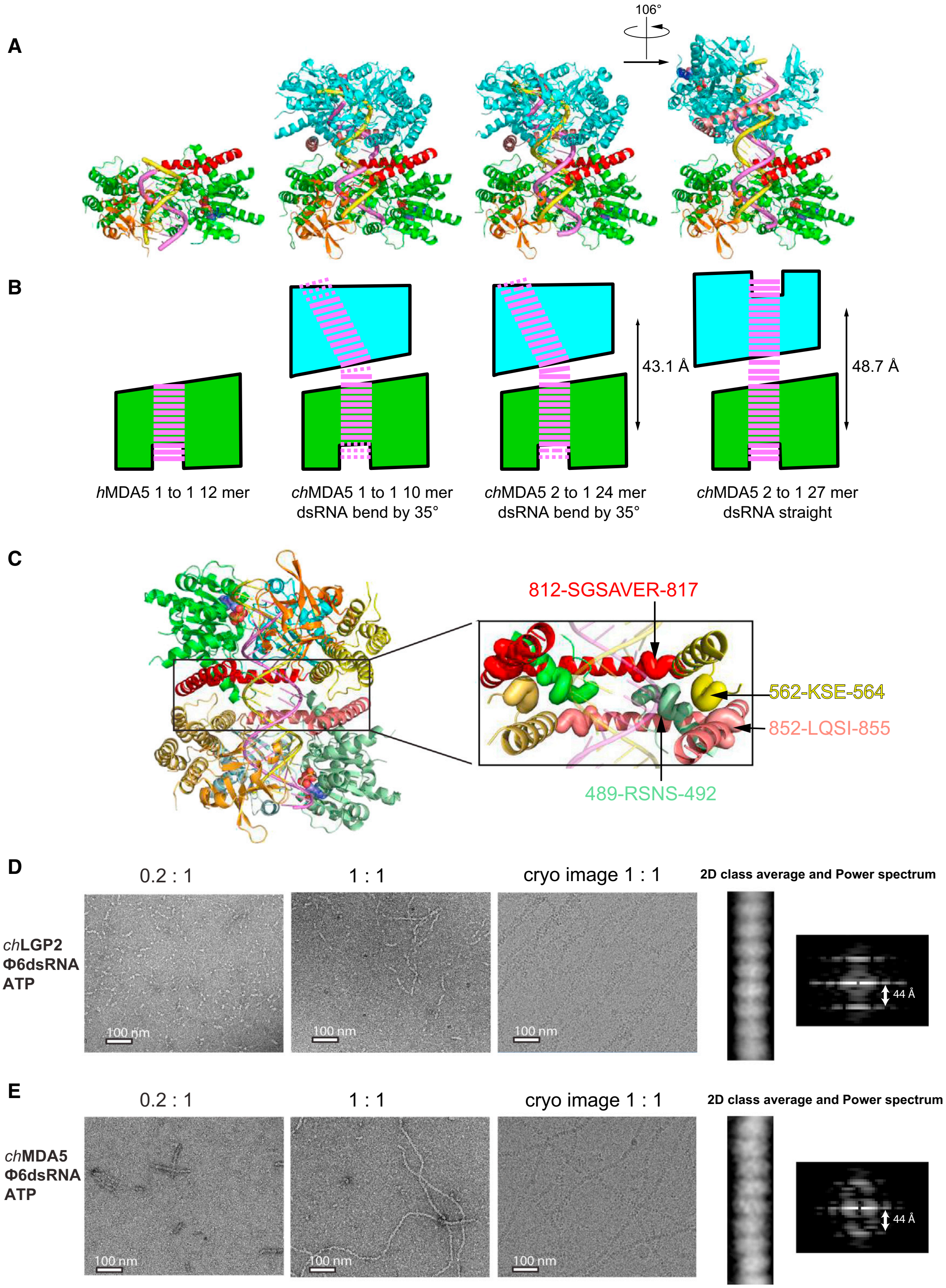

Figure 6. Dimer and Filament Formation by *ch*MDA5 and *ch*LGP2

(A) Comparison of monomeric and dimeric structures of MDA5-dsRNA complexes. *h*MDA5 12-mer (PDB: 4GL2) (left), *ch*MDA5 10-mer (middle-left), *ch*MDA5 24-mer (middle-right), and *ch*MDA5 27-mer (right). In all cases, the bottom molecule (green ribbons with CTD in orange, pincer domain in red) is in the same orientation. The top molecule is cyan except for the pincer domain (pink). The *ch*MDA5 10-mer and 24-mer structures are essentially the same apart from the lack of continuity of the dsRNA in the former. In both cases, the axis of the dsRNA is bent by 35° between the two head-to-head molecules, but in the *ch*MDA5 27-mer complex, it is straight.

(legend continued on next page)

CellPress

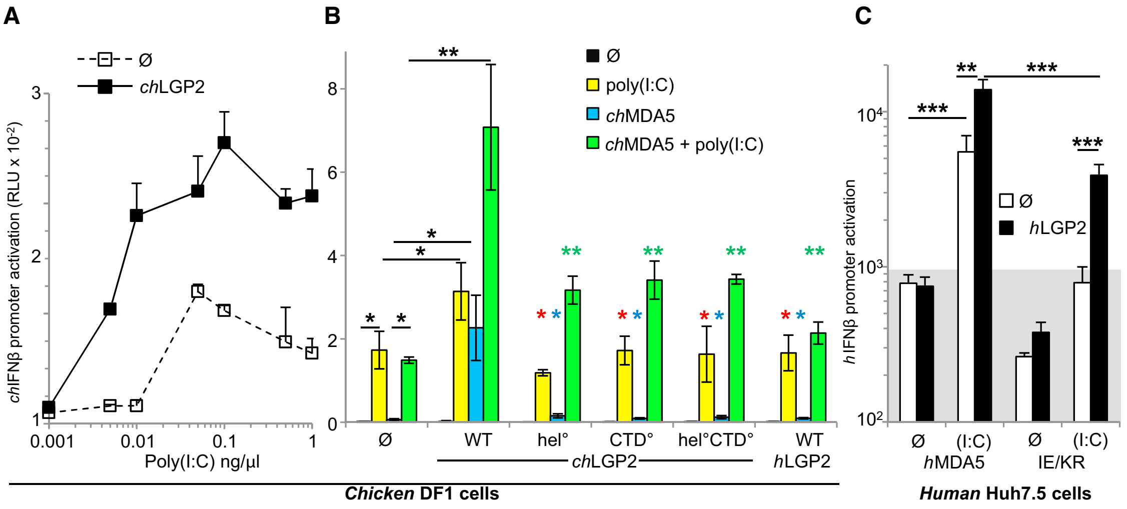

Figure 7. Cooperativity of LGP2 with MDA5 in Cells

(A) Enhancement of poly(I:C)-mediated activation of endogenous *ch*MDA5 by exogenously supplied *ch*LGP2 (27.5 ng DNA/well) in chicken DF1 cells. See also Figures S7A–S7C.

(B) Effect of *ch*LGP2, *ch*LGP2 variants (K138E/R490E or hel°, K648E/K649E or CTD°, K138E/R490E/K648E/K649E or hel°CTD°), and *h*LGP2 (27.5 ng DNA/well) on the activation of *ch*IFNβ promoter by endogenous *ch*MDA5 stimulated (yellow bars) or not (blue bars) by poly(I:C) and by exogenous *ch*MDA5 (1.67 ng DNA /well) in the absence (black bars) and presence (green bars) of poly(I:C) in chicken DF1 cells. *p < 0.05 and **p > 0.025 and below. Comparison of endogenous MDA5 and exogenous MDA5 co-transfected or not with LGP2 and activated or not with poly(I:C) are in black, LGP2 activated *ch*LGP2 variants with WT counterpart are in red (effect on endogenous *ch*MDA5), blue (effect on exogenous *ch*MDA5), and green (effect on exogenous *ch*MDA5 + poly[I:C]). Data are mean ± SD of three independent experiments, with each combination done in triplicate each time. See also Figures S7D–S7F.

(C) Effect of hLGP2 on the activation of exogenous *h*MDA5 and *h*MDA5 IE/KR (1.67 ng DNA/well) (I841K/E842R) mutant by poly(I:C) (denoted [I:C]) in the absence (white bars) and presence (black bars) of exogenously supplied *h*LGP2 (27.5 ng DNA/well) in human Huh7.5 cells. **p < 0.01 and ***p > 0.0025. Background signal (mean value) of cells transfected with control plasmid DNA followed or not by transfection of poly(I:C) is indicated by the shaded area. See also Figure S7G. See also Figure S7.

nucleotide, and at a ratio of 0.2:1, long clusters are observed on a few dsRNA molecules (Figure 6E). Thus, *ch*LGP2 binding to dsRNA is less co-operative than *ch*MDA5 (this work) and *h*MDA5 (Berke et al., 2012; Peisley et al., 2011; Wu et al., 2013). Imaging of *ch*LGP2 and full-length *ch*MDA5 filaments by cryo-EM shows that they are qualitatively similar, with a subunit axial translation of ~44 Å (Figures 6D and 6E), identical to that reported for *h*MDA5 (Berke et al., 2012).

Functional Studies of LGP2/MDA5 Cooperativity

The mechanism of *ch*LGP2 stimulation of *ch*MDA5 function was investigated using the RNA binding-deficient *ch*LGP2 mutants in the CTD, helicase, or both, which we characterized above and monitoring IFN-β activation in chicken DF1 cells upon stimulation with poly(I:C). Exogenously provided *ch*LGP2 readily augments the response of endogenous *ch*MDA5 to poly(I:C) (Figures 7A and S7A), as also found in the human system (Figures S7B and S7C), whereas LGP2 itself lacks transduction ability (Figures 7B and S7D) (Rothenfusser et al., 2005). Unlike the human system, in which exogenous *h*MDA5 exhibits a constitutive activity not enhanced by exogenous *h*LGP2 (Figure S7D, no RNA), exogenous *ch*MDA5 lacks constitutive activity, but co-transfection with *ch*LGP2 results in significant activation of the *ch*IFNβ promoter (Figure 7B, compare WT and Ø, blue bars) suggesting that *ch*LGP2 overexpression can stabilize the binding of *ch*MDA5 to cellular RNAs (as found with ATP hydrolysis mutants of MDA5; Louber et al., 2015). Co-transfection of *ch*LGP2 has a strong enhancing effect on the activation of limiting amounts of exogenous *ch*MDA5 by poly(I:C) (Figure 7B, compare green and blue bars for wild-type *ch*LGP2), similar to

(B) Schematic representation of (A) highlighting the dsRNA conformation. The distance between the center of mass of the two molecules in the head-to-head dimer is indicated. Compared with the *h*MDA5 12-mer structure (left), the *ch*MDA5 10-mer structure (middle left) lacks 3 bp at the tail end and gains 1 bp at the head end, and there is a 1 bp gap between the RNAs in the two head-to-head molecules in the dimer (dotted base pairs indicate potential extra base pairs not in the structure). In the *ch*MDA5 24-mer (middle right), the dsRNA is continuous between the molecules in the dimer but bent. Because there is 1 bp between the two MDA5 molecules and 24 bp overall, the structure is likely a superposition of structures with either 11 or 12 bp bound to one MDA5 (i.e., 11.5 on average), as indicated by the dashed line. In the low-resolution *ch*MDA5 27-mer, it is unclear whether there are extra base pairs between the molecules, which are more widely separated, or at the tail ends, but the dsRNA is straight.

(C) Detail of the four regions involved in protein-protein interactions mediating the head-to-head packing.

(D) EM analysis of *ch*LGP2:Φ6 dsRNA complexes in the presence of ATP. Left: molar ratio 0.2:1 with uranyl acetate negative stain. Middle left: molar ratio 1:1 with negative stain. Middle right: molar ratio 1:1 with cryo-EM. Right: cryo-EM image 2D class average and power spectrum.

(E) EM analysis of *ch*MDA5:Φ6 dsRNA complexes in the presence of ATP. Left: molar ratio 0.2:1 with negative stain. Middle left: molar ratio 1:1 with negative stain. Middle right: molar ratio 1:1 with cryo-EM. Right: cryo-EM image 2D class average and power spectrum.

the dose-dependent enhancement observed in the human system (Figures S7D and S7E) (Bruns et al., 2013, 2014; Pippig et al., 2009). Enhancing activities were most evident when either MDA5 or poly(I:C) was in limited amounts. Furthermore there appears to be a species restriction, as *h*LGP2 readily enhances *h*MDA5 activation in human Huh7.5 cells (Figures S7C–S7E) but is inactive toward *ch*MDA5 in DF1 cells (Figure 7B). RNA binding-deficient *ch*LGP2 mutants in the CTD, helicase, or both show poor enhancing activity toward *ch*MDA5 (Figure 7B, CTD°, hel°, and CTD°-hel° constructs), also observed in the human system (Figure S7F). Finally, *h*LGP2 also exhibited a potent enhancing effect on the poly(I:C)-mediated activation of the *h*MDA5 IE/KR mutant, which has been reported to be deficient in signaling because of a defect in homopolymerization (Louber et al., 2015; Wu et al., 2013) (Figure 7C), this property being abrogated with RNA binding-deficient *h*LGP2 mutants (Figure S7G).

DISCUSSION

We describe unusually high-resolution crystal structures of *ch*LGP2 bound to dsRNA and ADP:AlF_4, which show *ch*LGP2 to be in the fully closed, ATP hydrolysis transition state. *ch*LGP2 binds to dsRNA with its CTD capping the blunt end, similarly to RIG-I, but differs in having a longer overall footprint and by accommodating 5′ or 3′ overhangs with relatively minor perturbations in structure and little loss in affinity.

We also present several structures of *ch*MDA5, all of which are in the semi-closed state with ADP:Mg^{2+} bound. *ch*MDA5 seems to prefer head-to-head packing when bound to short dsRNA (24-mer to 27-mer) in contrast to the polar head-to-tail packing described for long, signaling active *h*MDA5-dsRNA filaments (Berke et al., 2012; Wu et al., 2013). *ch*LGP2 also forms filaments on dsRNA structurally resembling those of MDA5, although *ch*LGP2 dsRNA coating is less co-operative.

Functionally, conserved RNA binding determinants in both the CTD and helicase are required for LGP2-mediated enhancement of MDA5 signaling in both chicken and human, suggesting a conserved underlying mechanism for this phenomenon.

Open and Closed Helicase Conformations of RLHs

Structures of RIG-I and MDA5 can be classified into four states depending on the degree of closure of the Hel1 and Hel2 domains, and this is usually correlated to the type of bound nucleotide (Table S1; Figure S6A). RNA free structures of full-length RIG-I (in which the CARDs are bound to Hel2i) or the helicase domain alone are in the very flexible “open” conformation with Hel1 and Hel2 well separated and Hel2 poorly ordered (Kowalinski et al., 2011; Civril et al., 2011; Deimling et al., 2014). Most *h*RIG-I helicase-CTD-dsRNA structures with no nucleotide, sulfate, or ADP are in the “semi-open” conformation with the poorly ordered Hel2 not contacting the RNA (Kohlway et al., 2013; Luo et al., 2011, 2012). In one “semi-closed” state structure, co-crystallized with ATP analog ADP:BeF_3, Hel2 is better ordered, but motifs V and VI do not engage with the nucleotide (Jiang et al., 2011). The only fully “closed” RIG-I structure in which all motifs are correctly positioned for ATP hydrolysis, is that of the isolated dRIG-I helicase domain bound to dsRNA and ADP:AlF4 (Kowalinski et al., 2011). The closed conformation of the *ch*LGP2-dsRNA-ADP:AlF4 complexes reported here thus closely resembles the latter *d*RIG-I structure. In contrast, the *ch*LGP2 12-mer dsRNA structure without nucleotide is semi-closed with Hel2 poorly ordered, except where it contacts the RNA. Intriguingly, a closed structure of RIG-I including the CTD and an ATP analog has not yet been reported.

To characterize quantitatively these different conformations, we superposed, via the Hel1 domain, all RLH structures on the *ch*LGP2 closed state as reference. The rotation angle required to superpose the Hel2 domain of the test structure on that of the reference structure varies systematically in concordance with the qualitative description above, being 0° to 3° for the closed state, 7° to 13° for the semi-closed state, ~40° for the semi-open state, and 50° to 60° for the open state (Table S1).

The two different *ch*LGP2 conformations described here provide a unique opportunity to compare the closed and semi-closed states of the same RLH. The ~10° rotation of Hel2 between the closed state and semi-closed states corresponds to a shift of the backbone interactions made by Hel2 (mediated by motifs IVa and V) by one phosphate down the 3′ strand of the dsRNA, whereas the interactions of Hel1 (mediated by motifs Ia, Ib, Ic, and IIa) with the dsRNA are unchanged (Figures S6B and S6C). The movement of Hel2 is coupled to that of Hel2i, which pivots around its contact with the CTD (which itself does not shift much), while maintaining similar contacts with the RNA (Figure S6B). This comparison highlights an ATP-dependent structural transition, which in one sense can correspond to a co-operative tightening of dsRNA interactions upon ATP binding and in the other sense the relaxation of the grip upon the dsRNA upon ATP hydrolysis. This structural transition needs to be taken into account in understanding the role of ATP binding and hydrolysis in RLH function, including filament formation.

End Binder and/or Stem Binder?

The stem binder MDA5 and end-capping LGP2/RIG-I critically differ in the position of the CTD and the nature of the residues on the CTD “capping loop” (Figure S1). In LGP2 and RIG-I “capping loop” aromatic hydrophobic amino acids (Phe595, Phe599, and Trp602 in *ch*LGP2; Phe853 and Phe856 in *h*RIG-I) contact the blunt-end base pair of the dsRNA by edge-on or stacking interactions, essentially preventing binding to a continuous stem. In MDA5, the loop contains no bulky hydrophobic residues and is disordered in all structures of MDA5 bound to dsRNA so far. However, it is ordered in the structure of the *h*MDA5 CTD alone (PDB: 3GA3), where it forms an additional β strand to the main β sheet of the CTD rather than bulging out as in RIG-I and LGP2 (Figure 5D). As pointed out previously (Wu et al., 2013), MDA5 CTD is significantly displaced away from the dsRNA compared with RIG-I and is able to pack closer to Hel2i because of the 10 residues shorter α12 in MDA5 Hel2i. In *ch*MDA5, the pining of the CTD to Hel2i is reinforced by the wrapping of the extended loop between α14 and α15 of Hel2i (residues 629–646) over the CTD (e.g., Asp635 interacts with His914 and Arg916 of CTD domain). In *ch*MDA5, this α14-α15 loop is shorter than that of *h*MDA5 (638–670) but longer than in RIG-I or LGP2. The close CTD-Hel2i interaction combined with the less hydrophobic and less intrusive CTD capping loop allows MDA5 to bind internally to dsRNA.

Interestingly, *ch*LGP2 resembles a chimera, combining a MDA5-like helicase domain and a RIG-I like CTD. The similarity of LGP2 to MDA5 is reflected in common structural features, not found in RIG-I, such as the mode of nucleotide binding (Figures 1D and 4D) and the length of the Hel2i domain helices (Figure S1) and susceptibility to protein V. However, the hydrophobic nature of the LGP2 CTD capping loop is clearly RIG-I like. Corresponding to these mixed structural features, LGP2 has the strong blunt-end binding characteristic of RIG-I yet is also able to make MDA5-like filaments, albeit less efficiently. Perhaps the chimeric nature of the LGP2 structure allows its CTD to adopt a different position closer to Hel2i, allowing LGP2 to form filaments similarly to MDA5, whereas RIG-I does not form well-ordered, extended filaments. The similarity in overall appearance and axial subunit translation of LGP2 filaments to MDA5 filaments suggests that LGP2 could also form head-to-tail packing on long dsRNA. Furthermore, modeling suggests that in MDA5, the capping loop could not only play a role in dsRNA binding (Figure 5D) but also be involved in the tail-to-head protein-protein interface. The same could be true in the case of LGP2, although it is unclear what alternative interactions the aromatic residues on the capping loop of LGP2 might make. This and related questions will be answered only by high-resolution EM reconstructions of LGP2 and MDA5 filaments.

Head-to-head and Head-to-Tail Packing of MDA5

The head-to-head arrangement we observe for dimers of MDA5 bound to short dsRNA contrasts with the intermolecular packing arrangement derived from EM reconstructions of mouse MDA5-coated dsRNA filaments, which exhibit a polar head-to-tail packing (Berke et al., 2012; Wu et al., 2013). When the *ch*MDA5 structure was fitted into the EM map of the helical filament, the superposition also showed that the head-to-tail configuration fits significantly better (correlation coefficient 0.87 compared with 0.78 for head to head). However, the *ch*MDA5-24 structure indicates that on short dsRNA, and in the absence of stabilizing co-operative interactions forming an extended multimer, head-to-head stacking appears to be the preferred mode of interaction, and head-to-tail packing has not yet been observed in any crystal form. Thus it is possible that a head-to-head dimer nucleates filament formation. Interestingly, Dicer-related helicase 3, an ortholog of the Dicer and RIG-I that is essential for secondary small interfering RNA production in *Caenorhabditis elegans*, binds and recognizes 22G-RNA as a dimer, which has been modeled as a head-to-head dimer (Fitzgerald et al., 2014).

Biological Role of LGP2 as a Regulator of RLH Signaling

Various hypotheses exist as to how LGP2 might play positive and negative roles in RLH signaling (Rodriguez et al., 2014). The high affinity of LGP2 end binding to short dsRNA supports that it could potentially compete for cognate RIG-I ligands and thus exert a negative effect, as proposed by others (Pippig et al., 2009; Rothenfusser et al., 2005). However, the affinity for 5′ppp-dsRNA is slightly lower than for RIG-I, suggesting that an excess of LGP2 over RIG-I might be required, as observed by others (Childs et al., 2013). Such a high unbalance between the two proteins, both of which are similarly transcriptionally upregulated by type I IFN (Rothenfusser et al., 2005), remains to be demonstrated in natural infection conditions. LGP2 has a considerably higher affinity for non-phosphorylated blunt-end dsRNA than RIG-I and thus could act as sponge to prevent mis-activation of RIG-I by such RNAs, which may exist in the host cell. This would be an additional mechanism to promote self-discrimination or non-self-discrimination by RIG-I in parallel with the recently recognized role of the RIG-I ATPase activity and the CARD2-Hel2i interface to prevent deleterious constitutive activation of RIG-I by more weakly binding, non-cognate cellular RNAs (Anchisi et al., 2015; Lässig et al., 2015; Louber et al., 2015; Ramanathan et al., 2016; Rawling et al., 2015).

The mechanism of enhancement of MDA5 signaling by LGP2 in human cells has been proposed to depend on a direct effect of LGP2 on MDA5 filament formation (Bruns et al., 2014; Childs et al., 2013). It was concluded that LGP2 attenuates MDA5 filament length, giving rise to shorter complexes that more efficiently stimulate anti-viral signaling than longer MDA5 filaments (Bruns et al., 2014). In the chicken system, LGP2-stimulated MDA5 signaling could work by a similar mechanism. Additionally, our results suggest that LGP2 could potentially compensate for the lack of RIG-I through high-affinity end binding to a RIG-I like ligand, which could then nucleate, by RNA-dependent protein-protein interactions, signaling-competent MDA5 oligomer formation. Such a mechanism is supported by the observed strong RNA end binding mode of LGP2 (which is more permissive in terms of 3′ and 5′ modifications than RIG-I) and the functional requirement of an intact RNA binding site on both the CTD and helicase. Interestingly, the enhancing effect is observed only when RNA ligand and/or MDA5 are in limited amounts, as is likely to be the case early in infection.

In conclusion, our structural and functional results lay the basis for further studies to elucidate the exact mechanistic role of LGP2 in regulating MDA5 signaling by, for instance, determining the composition and structure of mixed filaments and/or oligomers containing both MDA5 and LGP2.

EXPERIMENTAL PROCEDURES

Protein Preparation and Crystallization

Purified *ch*MDA5 (full-length, residues 1–1,001), *ch*MDA5ΔCARD (residues 298–994), *ch*MDA5ΔCARD-E436Q constructs, and a SUMO fusion of full-length *ch*LGP2 (1–674), expressed in *Escherichia coli*, were mixed with dsRNA of various lengths, prepared by in vitro T7 transcription, and either AMPPNP or ADP:AlF_4 and initially screened for crystallization using a Cartesian robot.

Crystallography

Optimized crystals were flash frozen and diffraction data collected on various beamlines at the European Synchrotron Radiation Facility (ESRF). Data were processed with XDS (Kabsch, 2010) and further analyzed using the CCP4 suite (Winn et al., 2011). Structures were determined by molecular replacement and refined with REFMAC5 (Murshudov et al., 1997).

EM

Full-length *ch*MDA5 or *ch*LGP2 complexes with Φ6 bacteriophage dsRNA (Thermo Scientific), with or without ATP analogs, were examined by negative stain EM. For cryo-EM, 1:1 mixtures (one MDA5/LGP2 molecule per 15 bp of dsRNA) were frozen on glow-discharged grids (Quantifoil Micro Tools) and data collected with an FEI Polara microscope equipped with a K2 summit detector (Gatan). Power spectra were calculated from masked 2D class averages.

Fluorescence Polarization Anisotropy

*ch*MDA5, *ch*LGP2, or *h*RIG-I was titrated into dsRNA solutions made by annealing 5′-FAM-labeled 12-mer RNA with unlabeled cRNA. Anisotropic measurements were made with excitation wavelength 495 nm and emission wavelength 515 nm during 100 s and K_d values derived by curve fitting.

ATPase Activity Assays

dsRNA-dependent ATPase reactions were performed using a Malachite green assay kit (Bioassays) over 0.5–30 min, as detailed previously (Louber et al., 2015).

Cellular Assays

IFN-β promoter activation upon transient expression of expression vectors coding for chicken or human MDA5 and LGP2 and poly(I:C) stimulation in chicken DF1 and human Huh7.5 cells was determined as previously described (Louber et al., 2015)

ACCESSION NUMBERS

The accession numbers for the structure factors and coordinates reported in this paper are as follows: *ch*LGP2, 5′p 10-mer dsRNA:ADP:AlF_4:Mg^{2+}, PDB: 5JAJ; *ch*LGP2, 5′ppp 10-mer dsRNA:ADP:AlF_4:Mg^{2+}, PDB: 5JB2; *ch*LGP2, 5′ppp 3ovg 26-mer hairpin RNA:ADP:AlF_4:Mg^{2+}, PDB: 5JBG; *ch*LGP2, 5′p 12-mer dsRNA, PDB: 5JBJ; *ch*MDA5 1:1 complex, 5′p 10-mer dsRNA:ADP:Mg^{2+}, monoclinic form, PDB: 5JC3 (twinned), PDB: 5JCH (untwinned), orthorhombic form, PDB: 5JCF; *ch*MDA5 2:1 complex, 5′p 24-mer dsRNA:ADP:Mg^{2+}, PDB: 5JC7.

SUPPLEMENTAL INFORMATION

Supplemental Information includes Supplemental Experimental Procedures, seven figures, and one table and can be found with this article online at http://dx.doi.org/10.1016/j.molcel.2016.04.021.

AUTHOR CONTRIBUTIONS

E.U. designed experiments and performed biochemistry, crystallization, data collection, and structural analysis. M.L. helped with protein production. H.M. with the help of E.U. performed the EM analysis. J.B. and D.G. designed, performed, and/or analyzed cellular experiments. S.C. directed the project, performed structural analysis, and wrote the paper with input from the other authors.

ACKNOWLEDGMENTS

We thank members of the European Synchrotron Radiation Facility (ESRF)-European Molecular Biology Laboratory (EMBL) Joint Structural Biology Group for access to ESRF beamlines and the staffs of the EMBL high-throughput crystallization and eukaryotic expression facilities. We also thank Thomas Lunardi, Stefan Reich, Morana Dulic, and Jan Kadlec for advice and discussions and C. Rice, S. Goodbourn, D. Garcin, R. Cattaneo, and N. Rugli for reagents. This work used the platforms of the Grenoble Instruct Center (ISBG:UMS 3518 CNRS-CEA-UJF-EMBL) with support from the French Infrastructure for Integrated Structural Biology (ANR-10-INSB-05-02) and the Grenoble Alliance for Integrated Structural Cell Biology (ANR-10-LABX-49-01) within the Partnership for Structural Biology. The EM facility is supported by Rhône-Alpes Region, Fondation Recherche Medicale, Fonds FEDER, and GIS-Infrastructures en Biologie Sante et Agronomie. The project was supported consecutively by Fondation Innovations en Infectiologie, Agence Nationale de la Recherche (ANR) grant CARDINNATE (ANR12-BSV3-0010-01), and European Research Council Advanced Grant V-RNA (322586) to S.C. Plasmids are available upon request via the Addgene repository.

Received: October 8, 2015
Revised: March 21, 2016
Accepted: April 19, 2016
Published: May 19, 2016

REFERENCES

Ahmad, S., and Hur, S. (2015). Helicases in antiviral immunity: dual properties as sensors and effectors. Trends Biochem. Sci. *40*, 576–585.

Anchisi, S., Guerra, J., and Garcin, D. (2015). RIG-I ATPase activity and discrimination of self-RNA versus non-self-RNA. MBio *6*, e02349.

Barber, M.R., Aldridge, J.R., Jr., Webster, R.G., and Magor, K.E. (2010). Association of RIG-I with innate immunity of ducks to influenza. Proc. Natl. Acad. Sci. U S A *107*, 5913–5918.

Berke, I.C., Yu, X., Modis, Y., and Egelman, E.H. (2012). MDA5 assembles into a polar helical filament on dsRNA. Proc. Natl. Acad. Sci. U S A *109*, 18437–18441.

Bruns, A.M., Pollpeter, D., Hadizadeh, N., Myong, S., Marko, J.F., and Horvath, C.M. (2013). ATP hydrolysis enhances RNA recognition and antiviral signal transduction by the innate immune sensor, laboratory of genetics and physiology 2 (LGP2). J. Biol. Chem. *288*, 938–946.

Bruns, A.M., Leser, G.P., Lamb, R.A., and Horvath, C.M. (2014). The innate immune sensor LGP2 activates antiviral signaling by regulating MDA5-RNA interaction and filament assembly. Mol. Cell *55*, 771–781.

Chen, S., Cheng, A., and Wang, M. (2013). Innate sensing of viruses by pattern recognition receptors in birds. Vet. Res. (Faisalabad) *44*, 82.

Childs, K., Randall, R., and Goodbourn, S. (2012). Paramyxovirus V proteins interact with the RNA Helicase LGP2 to inhibit RIG-I-dependent interferon induction. J. Virol. *86*, 3411–3421.

Childs, K.S., Randall, R.E., and Goodbourn, S. (2013). LGP2 plays a critical role in sensitizing mda-5 to activation by double-stranded RNA. PLoS ONE *8*, e64202.

Civril, F., Bennett, M., Moldt, M., Deimling, T., Witte, G., Schiesser, S., Carell, T., and Hopfner, K.P. (2011). The RIG-I ATPase domain structure reveals insights into ATP-dependent antiviral signalling. EMBO Rep. *12*, 1127–1134.

Deddouche, S., Goubau, D., Rehwinkel, J., Chakravarty, P., Begum, S., Maillard, P.V., Borg, A., Matthews, N., Feng, Q., van Kuppeveld, F.J., and Reis e Sousa, C. (2014). Identification of an LGP2-associated MDA5 agonist in picornavirus-infected cells. eLife *3*, e01535.

Deimling, T., Cui, S., Lammens, K., Hopfner, K.P., and Witte, G. (2014). Crystal and solution structure of the human RIG-I SF2 domain. Acta Crystallogr. F Struct. Biol. Commun. *70*, 1027–1031.

Fairman-Williams, M.E., Guenther, U.P., and Jankowsky, E. (2010). SF1 and SF2 helicases: family matters. Curr. Opin. Struct. Biol. *20*, 313–324.

Fitzgerald, M.E., Vela, A., and Pyle, A.M. (2014). Dicer-related helicase 3 forms an obligate dimer for recognizing 22G-RNA. Nucleic Acids Res. *42*, 3919–3930.

Funabiki, M., Kato, H., Miyachi, Y., Toki, H., Motegi, H., Inoue, M., Minowa, O., Yoshida, A., Deguchi, K., Sato, H., et al. (2014). Autoimmune disorders associated with gain of function of the intracellular sensor MDA5. Immunity *40*, 199–212.

Goubau, D., Deddouche, S., and Reis e Sousa, C. (2013). Cytosolic sensing of viruses. Immunity *38*, 855–869.

Hayashi, T., Watanabe, C., Suzuki, Y., Tanikawa, T., Uchida, Y., and Saito, T. (2014). Chicken MDA5 senses short double-stranded RNA with implications for antiviral response against avian influenza viruses in chicken. J. Innate Immun. *6*, 58–71.

Hopfner, K.P. (2014). RIG-I holds the CARDs in a game of self versus nonself. Mol. Cell *55*, 505–507.

Jang, M.A., Kim, E.K., Now, H., Nguyen, N.T., Kim, W.J., Yoo, J.Y., Lee, J., Jeong, Y.M., Kim, C.H., Kim, O.H., et al. (2015). Mutations in DDX58, which encodes RIG-I, cause atypical Singleton-Merten syndrome. Am. J. Hum. Genet. *96*, 266–274.

Jiang, F., Ramanathan, A., Miller, M.T., Tang, G.Q., Gale, M., Jr., Patel, S.S., and Marcotrigiano, J. (2011). Structural basis of RNA recognition and activation by innate immune receptor RIG-I. Nature *479*, 423–427.

Kabsch, W. (2010). Xds. Acta Crystallogr. D Biol. Crystallogr. *66*, 125–132.

Karpala, A.J., Stewart, C., McKay, J., Lowenthal, J.W., and Bean, A.G. (2011). Characterization of chicken Mda5 activity: regulation of IFN-β in the absence of RIG-I functionality. J. Immunol. *186*, 5397–5405.

Kohlway, A., Luo, D., Rawling, D.C., Ding, S.C., and Pyle, A.M. (2013). Defining the functional determinants for RNA surveillance by RIG-I. EMBO Rep. *14*, 772–779.

Kowalinski, E., Lunardi, T., McCarthy, A.A., Louber, J., Brunel, J., Grigorov, B., Gerlier, D., and Cusack, S. (2011). Structural basis for the activation of innate immune pattern-recognition receptor RIG-I by viral RNA. Cell *147*, 423–435.

Lässig, C., Matheisl, S., Sparrer, K.M., de Oliveira Mann, C.C., Moldt, M., Patel, J.R., Goldeck, M., Hartmann, G., García-Sastre, A., Hornung, V., et al. (2015). ATP hydrolysis by the viral RNA sensor RIG-I prevents unintentional recognition of self-RNA. eLife *4*, 4.

Li, X., Ranjith-Kumar, C.T., Brooks, M.T., Dharmaiah, S., Herr, A.B., Kao, C., and Li, P. (2009). The RIG-I-like receptor LGP2 recognizes the termini of double-stranded RNA. J. Biol. Chem. *284*, 13881–13891.

Liniger, M., Summerfield, A., Zimmer, G., McCullough, K.C., and Ruggli, N. (2012). Chicken cells sense influenza A virus infection through MDA5 and CARDIF signaling involving LGP2. J. Virol. *86*, 705–717.

Louber, J., Brunel, J., Uchikawa, E., Cusack, S., and Gerlier, D. (2015). Kinetic discrimination of self/non-self RNA by the ATPase activity of RIG-I and MDA5. BMC Biol. *13*, 54.

Luo, D., Ding, S.C., Vela, A., Kohlway, A., Lindenbach, B.D., and Pyle, A.M. (2011). Structural insights into RNA recognition by RIG-I. Cell *147*, 409–422.

Luo, D., Kohlway, A., Vela, A., and Pyle, A.M. (2012). Visualizing the determinants of viral RNA recognition by innate immune sensor RIG-I. Structure *20*, 1983–1988.

Murshudov, G.N., Vagin, A.A., and Dodson, E.J. (1997). Refinement of macromolecular structures by the maximum-likelihood method. Acta Crystallogr. D Biol. Crystallogr. *53*, 240–255.

Peisley, A., Lin, C., Wu, B., Orme-Johnson, M., Liu, M., Walz, T., and Hur, S. (2011). Cooperative assembly and dynamic disassembly of MDA5 filaments for viral dsRNA recognition. Proc. Natl. Acad. Sci. U S A *108*, 21010–21015.

Peisley, A., Wu, B., Xu, H., Chen, Z.J., and Hur, S. (2014). Structural basis for ubiquitin-mediated antiviral signal activation by RIG-I. Nature *509*, 110–114.

Pippig, D.A., Hellmuth, J.C., Cui, S., Kirchhofer, A., Lammens, K., Lammens, A., Schmidt, A., Rothenfusser, S., and Hopfner, K.P. (2009). The regulatory domain of the RIG-I family ATPase LGP2 senses double-stranded RNA. Nucleic Acids Res. *37*, 2014–2025.

Ramanathan, A., Devarkar, S.C., Jiang, F., Miller, M.T., Khan, A.G., Marcotrigiano, J., and Patel, S.S. (2016). The autoinhibitory CARD2-Hel2i Interface of RIG-I governs RNA selection. Nucleic Acids Res. *44*, 896–909.

Rawling, D.C., Fitzgerald, M.E., and Pyle, A.M. (2015). Establishing the role of ATP for the function of the RIG-I innate immune sensor. eLife *4*, 4.

Rodriguez, K.R., and Horvath, C.M. (2013). Amino acid requirements for MDA5 and LGP2 recognition by paramyxovirus V proteins: a single arginine distinguishes MDA5 from RIG-I. J. Virol. *87*, 2974–2978.

Rodriguez, K.R., Bruns, A.M., and Horvath, C.M. (2014). MDA5 and LGP2: accomplices and antagonists of antiviral signal transduction. J. Virol. *88*, 8194–8200.

Rothenfusser, S., Goutagny, N., DiPerna, G., Gong, M., Monks, B.G., Schoenemeyer, A., Yamamoto, M., Akira, S., and Fitzgerald, K.A. (2005). The RNA helicase Lgp2 inhibits TLR-independent sensing of viral replication by retinoic acid-inducible gene-I. J. Immunol. *175*, 5260–5268.

Satoh, T., Kato, H., Kumagai, Y., Yoneyama, M., Sato, S., Matsushita, K., Tsujimura, T., Fujita, T., Akira, S., and Takeuchi, O. (2010). LGP2 is a positive regulator of RIG-I- and MDA5-mediated antiviral responses. Proc. Natl. Acad. Sci. U S A *107*, 1512–1517.

Winn, M.D., Ballard, C.C., Cowtan, K.D., Dodson, E.J., Emsley, P., Evans, P.R., Keegan, R.M., Krissinel, E.B., Leslie, A.G., McCoy, A., et al. (2011). Overview of the CCP4 suite and current developments. Acta Crystallogr. D Biol. Crystallogr. *67*, 235–242.

Wu, B., Peisley, A., Richards, C., Yao, H., Zeng, X., Lin, C., Chu, F., Walz, T., and Hur, S. (2013). Structural basis for dsRNA recognition, filament formation, and antiviral signal activation by MDA5. Cell *152*, 276–289.

Wu, B., Peisley, A., Tetrault, D., Li, Z., Egelman, E.H., Magor, K.E., Walz, T., Penczek, P.A., and Hur, S. (2014). Molecular imprinting as a signal-activation mechanism of the viral RNA sensor RIG-I. Mol. Cell *55*, 511–523.

Xiol, J., Spinelli, P., Laussmann, M.A., Homolka, D., Yang, Z., Cora, E., Couté, Y., Conn, S., Kadlec, J., Sachidanandam, R., et al. (2014). RNA clamping by Vasa assembles a piRNA amplifier complex on transposon transcripts. Cell *157*, 1698–1711.

Yoo, J.S., Kato, H., and Fujita, T. (2014). Sensing viral invasion by RIG-I like receptors. Curr. Opin. Microbiol. *20*, 131–138.

Zhu, Z., Zhang, X., Wang, G., and Zheng, H. (2014). The laboratory of genetics and physiology 2: emerging insights into the controversial functions of this RIG-I-like receptor. BioMed Res. Int. *2014*, 960190.

Molecular Cell, Volume *62*

Supplemental Information

Structural Analysis of dsRNA Binding to Anti-viral Pattern Recognition Receptors LGP2 and MDA5

Emiko Uchikawa, Mathilde Lethier, Hélène Malet, Joanna Brunel, Denis Gerlier, and Stephen Cusack

Structural analysis of dsRNA binding to anti-viral pattern recognition receptors LGP2 and MDA5

Emiko Uchikawa[1,2], Mathilde Lethier[1,2], Hélène Malet[1,2],

Joanna Brunel[3-7], Denis Gerlier[3-7] and Stephen Cusack[1,2#]

[1]European Molecular Biology Laboratory, Grenoble Outstation, 71 Avenue des Martyrs, CS 90181, 38042 Grenoble Cedex 9, France

[2]University Grenoble Alpes-Centre National de la Recherche Scientifique-EMBL Unit of Virus Host-Cell Interactions, 71 Avenue des Martyrs, CS 90181, 38042 Grenoble Cedex 9, France.

[3]CIRI, International Center for Infectiology Research, Université de Lyon, Lyon, France;

[4]Inserm, U1111, Lyon, France;

[5]CNRS, UMR5308, Lyon, France;

[6]Ecole Normale Supérieure de Lyon, Lyon, France;

[7]Université Lyon 1, Centre International de Recherche en Infectiologie, Lyon, France

#Corresponding author:

Stephen Cusack

European Molecular Biology Laboratory, Grenoble Outstation, Grenoble Cedex 9, France.

Tel. (33)476207238, Email: cusack@embl.fr

Supplemental Information

- Supplementary Figure Legends S1-S7
- Supplementary Figures
- Supplementary Table S1
- Supplemental Experimental Procedures
- Supplemental References.

Supplementary Figure Legends.

Figure S1 Sequence alignment of chicken and human LGP2 and MDA5 and duck and human RIG-I (excluding the CARDs). Related to Figures 1 and 4.

The secondary structure of *ch*LGP2 and *ch*MDA5 are at the top and bottom respectively. Outline boxes in green, yellow, cyan, red and orange delimit the Hel1, Hel2i, Hel2, Pincer and CTD domains. Helicase conserved motifs are indicated in black on the bottom and important loops such as the Hel2i, Hel2, end-binding and capping loops are highlighted with a green, yellow, cyan, magenta or orange background respectively. Figure made with ESPript (Gouet et al., 1999).

Figure S2. ATP analogue binding and ATP hydrolysis by *ch*LGP2. Related to Figure 1.

(A) Diagram showing interaction network between *ch*LGP2 and ADP:AlF_4:Mg^{2+}. Residue colours are as in Figure1C with polar interactions (blue) and hydrophobic interactions (black).

(B) Ligand electron density for ADP:AlF_4:Mg^{2+} in the *ch*LGP2 5′p 10-mer dsRNA complex with Mg^{2+} (magenta), Al (grey), F (pale blue) and coordinated water (marine). Final 2Fo-Fc electron density is contoured at 2.0 σ.

(C) Comparative RNA-dependent ATP hydrolysis activity by LGP2, RIG-I and MDA5 showing initial reaction velocities versus ATP concentration. The concentration of proteins and dsRNAs are 0.125 µM *d*Rig-I and 0.5 µM dsRNA, 0.25 µM *ch*LGP2 and 1 µM dsRNA, 0.5 µM *ch*MDA5 and 2 µM dsRNA. Plotted values are mean ±SD (n=3).

(D) ATPase activity of RLHs as a function dsRNA length using 18-mer hairpin (5′-GGGCGGCUUCGGCCGCCC-3′), 24-mer (same dsRNA as for crystallization), 32-mer hairpin (5′-pppGGGCGAGCGUGCGCUUCGGCGCACGCUCGCCC-3′), or 38-mer (5′-GGGACGUAGCAUCCGAUGUACAUCGGAUGCUACGUCCC-3′). The graph compares the amount of hydrolysis after 16 minutes. The reaction conditions were 1 µM RLH, 4 µM dsRNA and 2 mM ATP. Poly(I:C) is polyinosinic-polycytidylic acid (Amersham Biosciences).

(E) Multiple sequence alignment of Motif I of RLHs

(F) Multiple sequence alignment of Motif VI of RLHs

Figure S3. Sequence alignment of LGP2 from representative vertebrates. Related to Figure 1.

Sequences aligned include those from birds (chicken, Muscovy duck), mammals (human, mouse), reptile (alligator), amphibians (frog) and fish (zebrafish). Annotation is similar to Figure S1.

Figure S4. 5′ tri-phosphate conformation and summary of RNAs used. Related to Figures 2, 3 and 5.

(A) Schematic diagram showing the interactions with the 5′ tri-phosphate in the *ch*LGP2_10ppp structure.

(B) Schematic diagram showing the interactions with the 5′ tri-phosphate in the *ch*LGP2_3′ovg structure.

(C) RNAs used in various experiments.

Figure S5. Comparative binding of RLHs to dsRNA. Related to Figure 2.

(A) Electron density showing residues from Hel2i helix α10 interacting directly or via water molecules with nucleotides from the 3′ (violet) and 5′ (yellow) strands in the *ch*LGP2 5′p 10-mer dsRNA complex. Final 2Fo-Fc electron density is contoured at 1.5 σ.

(B) Representative curves of fluorescence anisotropy changes measured by titrating full-length *h*RIG-I (upper panel), *ch*LGP2 (middle panel) or *ch*MDA5ΔCARD (bottom panel) to FAM labelled 5′OH, 5′ppp, 3′ overhang and 5′ overhang 12-mer dsRNA with or without various nucleotides.

Figure S6. Comparison of helicase opening in known RLH crystal structures and comparison of the closed and semi-closed states of *ch*LGP2. Related to all Figures.

(A) Disposition of helicase Hel1 (green) and Hel2 (cyan) domains for known RLH crystal structures (with PDB entry code indicated) after superposition of Hel1 of each structure onto that of the closed state of *ch*LGP2, used as reference. ATP analogues (slate blue) and Mg^{2+} (magenta) are shown with spheres and the dsRNA back bone (orange). Other domains are coloured as in Fig 1A. According to the relative displacement of Hel1 and Hel2, the structures are classified closed, semi-closed, semi-open and opened (see Table S1 for quantification).

(B) Comparison of the structures of the closed and semi-closed states of *ch*LGP2 after superposition of the Hel1 domain. The Hel1, Hel2, Hel2i and CTD domains are coloured green (pale green), cyan (pale cyan), yellow (sand) and orange (pale orange) for respectively and the dsRNA 5′ strand violet (pale violet) and 3′ strand yellow (sand) for the closed (semi-closed) states respectively.

(C) Schematic diagram comparing interactions made in the closed (left) and semi-closed (right) states of *ch*LGP2 to the dsRNA illustrating the shift of Hel2 interactions (red box) one phosphate down the 3′ strand.

Figure S7 . Co-operativity of LGP2 with MDA5 in human cells. Related to Figure 7.

(A) Expression of *ch*LGP2, *ch*LGP2 variants and *h*LGP2 in chicken DF1 cells (top) and expression of *h*LGP2 and *h*LGP2 variants in human Huh7.5 cells (bottom). The protein expression of other MDA5 constructs used in this work has been previously determined and found to be comparable to that of their *wt* counterpart (Louber et al., 2015).

(B) RIG-I and MDA5-mediated endogenous response to dsRNA in human HEK293 (in blue) and Huh7.5 (in red) cells. Activation of *hu*IFNβ promoter by RIG-I agonist 5′ppp-dsRNA (61mer), MDA5 agonist Φ6 bacteriophage 2.9, 4.1 and 6.4 kbp long dsRNA, and RIG-I/MDA5 agonist poly(I:C). Note the lack of detectable endogenous response observed in Huh7.5 cells and the neutral effect of the pCG-duF vector (noted "F") used as a complement loading DNA to ensure identical amounts of DNA in the experiments with expression vectors as a source of exogenous MDA5 and/or LGP2. (Related to Figure 7B)

(C) Enhancing effect of exogenously supplied *h*LGP2 on endogenous response to poly(I:C) in human HEK293 cells with the unit 1 corresponding to 5.5 ng LGP2 DNA/well. (Related to Figure 7A).

(D) Lack of signalling ability of exogenous *h*LGP2 upon activation with poly(I:C) or 5′ ppp-dsRNA (left) and dose-response enhancement by *h*LGP2 of the activation of exogenous *h*MDA5 by poly(I:C) (right) in human Huh7.5 cells. Note the high constitutive activation by MDA5_1 (corresponding to 5.5 ng DNA/well) (as

previously reported see (Louber et al., 2015) and references therein, see also panel E below, upper left histogram, condition 0 RNA) that is neither enhanced by exogenously added *h*LGP2 (compare histograms with no RNA (0) nor upon transfection of RIG-I agonist $^{5'ppp}$dsRNA with or without exogenous *h*LGP2 (compare histograms with 0 and $^{5'ppp}$dsRNA conditions). (Related to Figure 7B).

(E) Enhancing effect of *h*LGP2 on exogenous *h*MDA5 is observed only when transfected in limited amount and/or activated by a sub-optimal amount poly(I:C) in human Huh7.5 cells. Note the loss of dose-dependent enhancing effect of *h*LGP2 (with the unit 1 corresponding to 5.5 ng LGP2 DNA/well) in the presence of 0.05 ng versus 0.005 ng of poly(I:C) in the presence of 0.55, 1,67 and 5.5 ng/well of *h*MDA5 DNA (MDA5_0.1, MDA5_0.3, MDA5_1 at the upper right, bottom left and bottom right, respectively) and the reduction of this enhancing effect with increasing amount of *h*MDA5 (compare luciferase signal gradation according to *h*LGP2 amounts for a same amount of poly(I:C) in the presence of *h*MDA5 (MDA5_0.1, MDA5_0.3, MDA5_1)). Note also that the enhancing effect of *h*LGP2 on *h*MDA5 is restricted to poly(I:C) and is not observed with another MDA5 RNA agonist, the Φ6 bacteriophage 2.9, 4.1 and 6.4 kbp long dsRNA, while *h*MDA5 do respond also to this agonist in the absence of *hu*LGP2 (compare Φ6 and the no (0) conditions in the upper left panel for each amount of transfected *h*MDA5). (Related to Figure 7B).

(F) RNA binding deficient *h*LGP2 mutants (K138E/R492E or hel°, K650E/K651E or CTD°, K138E/R492E/K650E/K651E or hel°CTD°) but not ATPase-deficient E132Q mutant (see Figure 1E for ATPase activity) no longer exhibit enhancing activity for *h*MDA5 activated by poly(I:C) in human Huh7.5 cells (Related to Figure 7B).

(G) RNA binding deficient *h*LGP2 mutants (K138E/R492E or hel°, K650E/K651E or CTD°, K138E/R492E/K650E/K651E or hel°CTD°) but not ATPase-deficient E132Q

mutant no longer exhibit enhancing activity for the homopolymerisation-defective *h*MDA5 IE/KR mutant activated by poly(I:C) in human Huh7.5 cells. Cells were transfected with a mixture of LGP2 (27.5 ng DNA/well) and MDA5 (1.67 ng DNA/well), i.e. at 5:0.3 ratio (Related to Figure 7C).

Figure S1

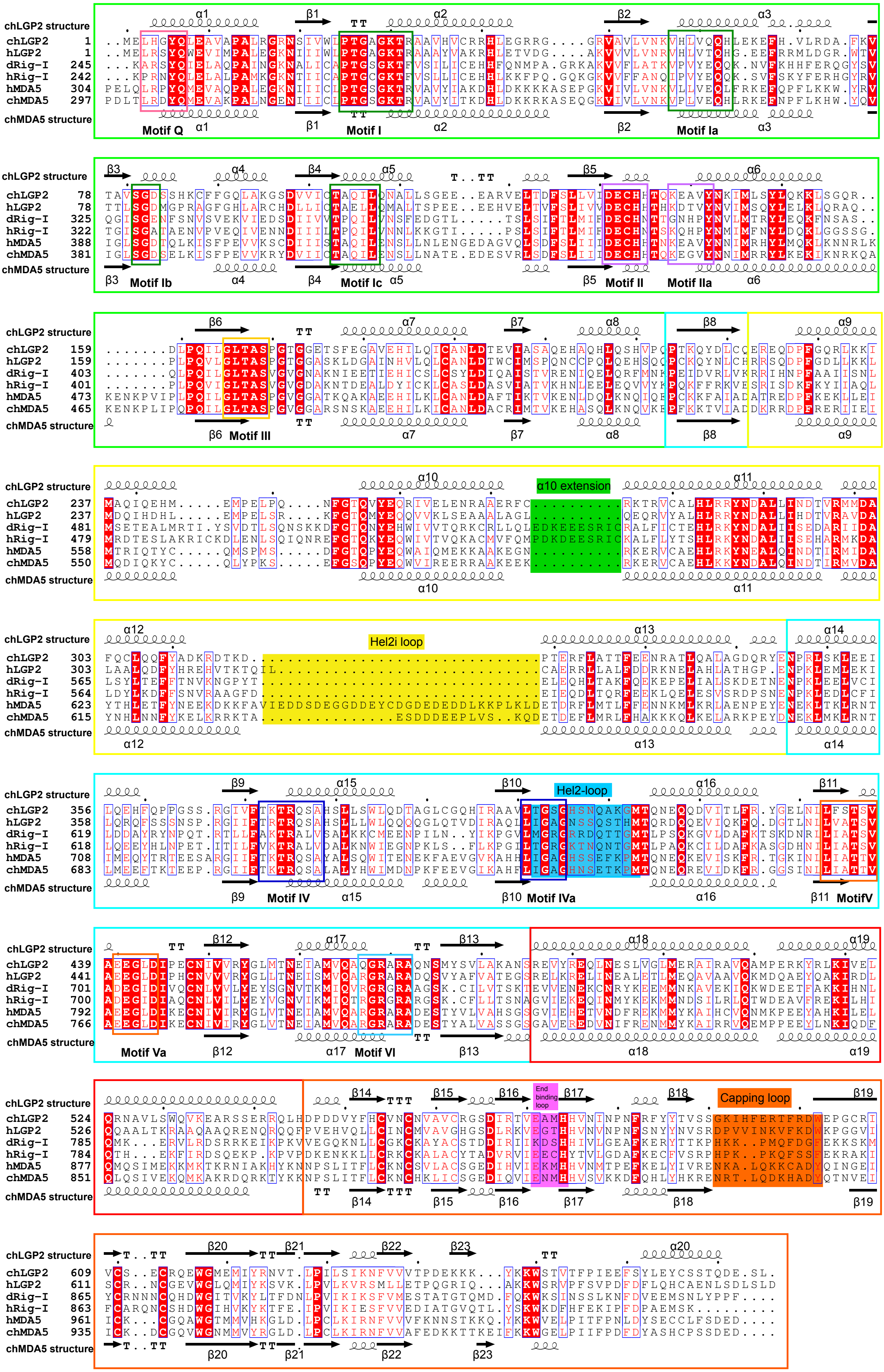
chLGP2 structure
chLGP2
hLGP2
dRig-I
hRig-I
hMDA5
chMDA5
chMDA5 structure
Motif Q
Motif I
Motif Ia
Motif Ib
Motif Ic
Motif II
Motif IIa
Motif III
α10 extension
Hel2i loop
Hel2-loop
Motif IV
Motif IVa
MotifV
Motif Va
Motif VI
End binding loop
Capping loop

Figure S2

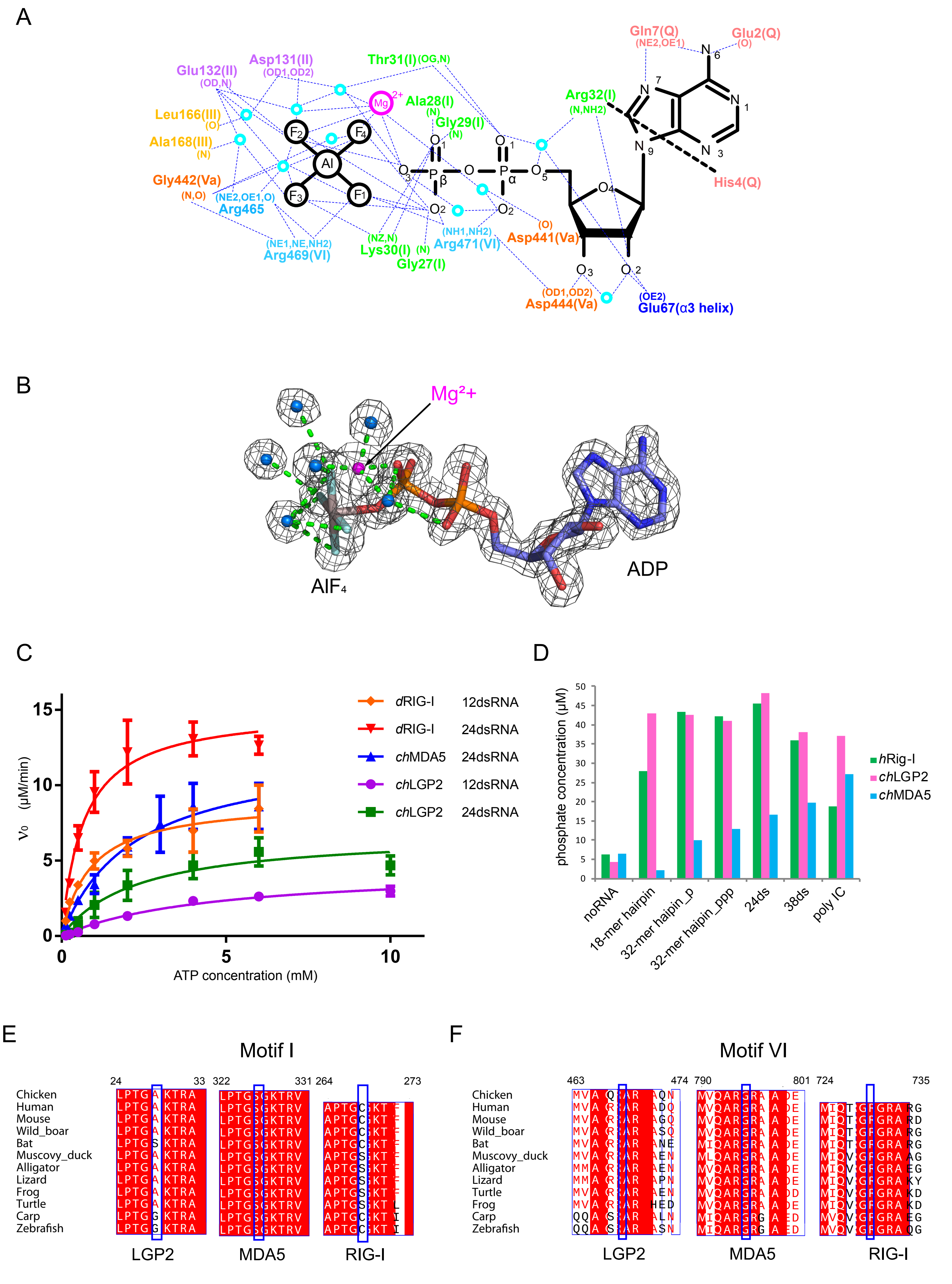
A
B
Mg²+
AlF₄
ADP
C
dRIG-I 12dsRNA
dRIG-I 24dsRNA
chMDA5 24dsRNA
chLGP2 12dsRNA
chLGP2 24dsRNA
v₀ (μM/min)
ATP concentration (mM)
D
phosphate concentration (μM)
hRig-I
chLGP2
chMDA5
noRNA
18-mer hairpin
32-mer haipin_p
32-mer haipin_ppp
24ds
38ds
poly IC
E
Motif I
F
Motif VI
Chicken
Human
Mouse
Wild_boar
Bat
Muscovy_duck
Alligator
Lizard
Frog
Turtle
Carp
Zebrafish
LGP2
MDA5
RIG-I

Figure S3

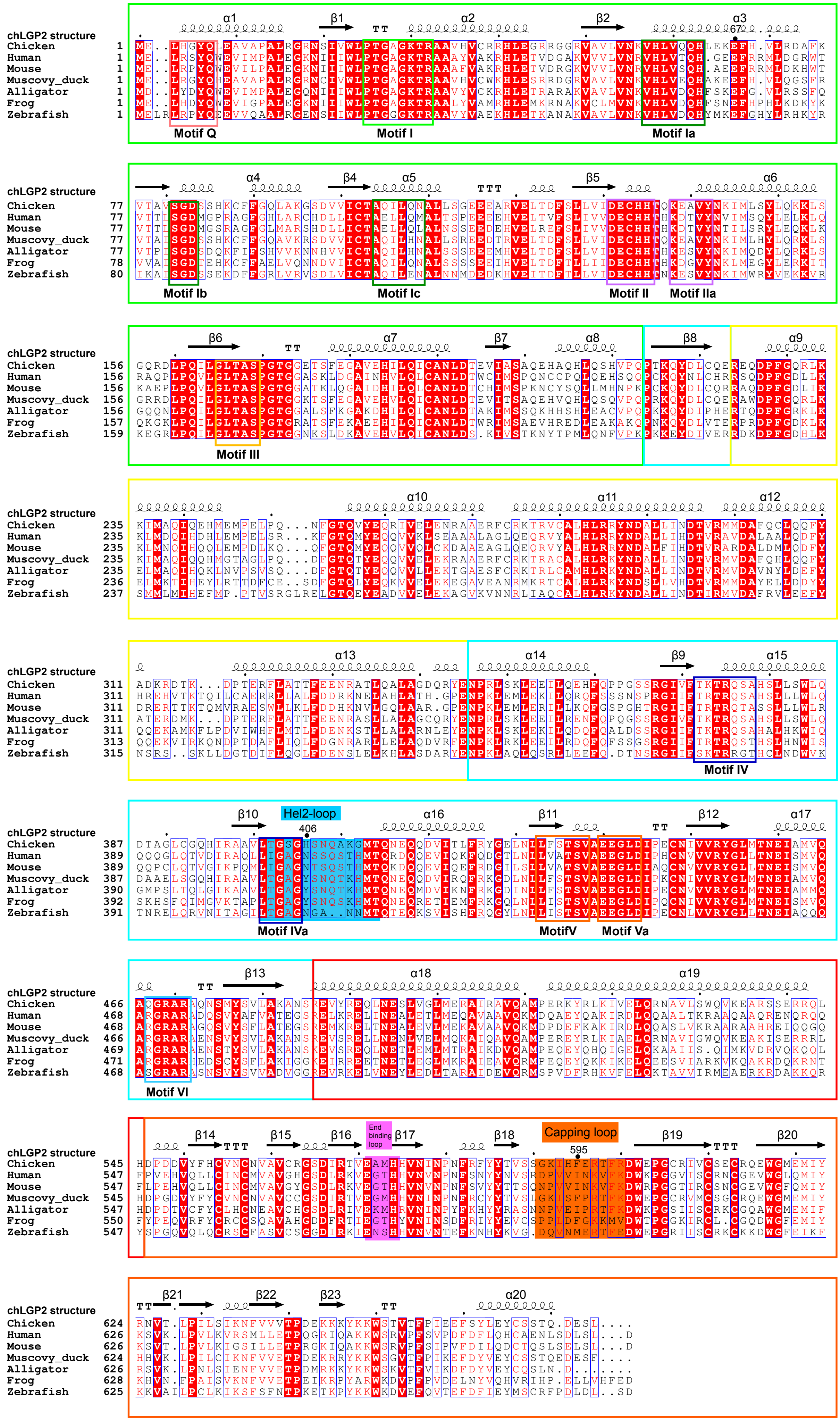

chLGP2 structure
Chicken
Human
Mouse
Muscovy_duck
Alligator
Frog
Zebrafish
Motif Q
Motif I
Motif Ia
Motif Ib
Motif Ic
Motif II
Motif IIa
Motif III
Motif IV
Hel2-loop
Motif IVa
MotifV
Motif Va
Motif VI
End binding loop
Capping loop

Figure S4

A

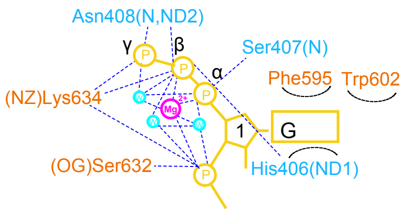

B

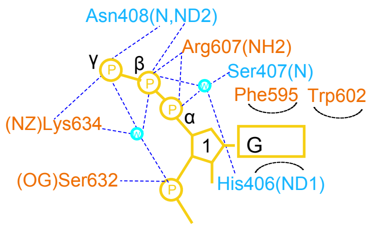

C

Anisotropy	RNA
12 dsRNA$_{OH}$	5′ – $_{OH}$GGGCUCCACAUG –3′<br>3′ – CCCGAGGUGUAC$_{(FAM)}$ –5′
12 dsRNA$_{ppp}$	5′ – $_{ppp}$GGGCUCCACAUG –3′<br>3′ – CCCGAGGUGUAC$_{(FAM)}$ –5′
3’ overhang$_{OH}$	5′ – $_{OH}$GGCCAGUGCGAA –3′<br>3′ – AACCGGUCACGC$_{(FAM)}$ –5′
5’ overhang$_{OH}$	5′ – $_{OH}$AACCGGUCACGC –3′<br>3′ – $_{(FAM)}$GGCCAGUGCGAA$_{OH}$ –5′
*ch*LGP2 crystallisation	
10 dsRNA$_{p}$	5′ – $_{p}$GGUACGUACC –3′<br>3′ – CCAUGCAUGG$_{p}$ –5′
10 dsRNA$_{ppp}$	5′ – $_{ppp}$GGUACGUACC –3′<br>3′ – CCAUGCAUGG$_{ppp}$ –5′
12 dsRNA$_{p}$	5′ – $_{p}$GGUAGCGCUACC –3′<br>3′ – CCAUCGCGAUGG$_{p}$ –5′
5′-ppp and 3' two nucleotide (GG) overhang hairpin RNA duplex	5′ – $_{ppp}$GGAGCGUGCCG U<br>3′ – GGCCUCGCACGGC A
*ch*MDA5 crystallisation	
10 dsRNA$_{p}$	5′ – $_{p}$GGUACGUACC –3′<br>3′ – CCAUGCAUGG$_{p}$ –5′
24 dsRNA$_{p}$	5′ – $_{p}$GGGACGUCAUGCGCAUGACGUCCC –3′<br>3′ – CCCUGCAGUACGCGUACUGCAGGG$_{p}$ –5′
26 dsRNA$_{p}$	5′ – $_{p}$GGGACGUUCAUGCGCAUGAACGUCCC –3′<br>3′ – CCCUGCAAGUACGCGUACUUGCAGGG$_{p}$ –5′
27 dsRNA$_{p}$	5′ – $_{p}$GGGCACGUGCAGGUCCUGCACGUGCCC –3′<br>3′ – CCCGUGCACGUCCAGGACGUGCACGGG$_{p}$ –5′

Figure S5

A

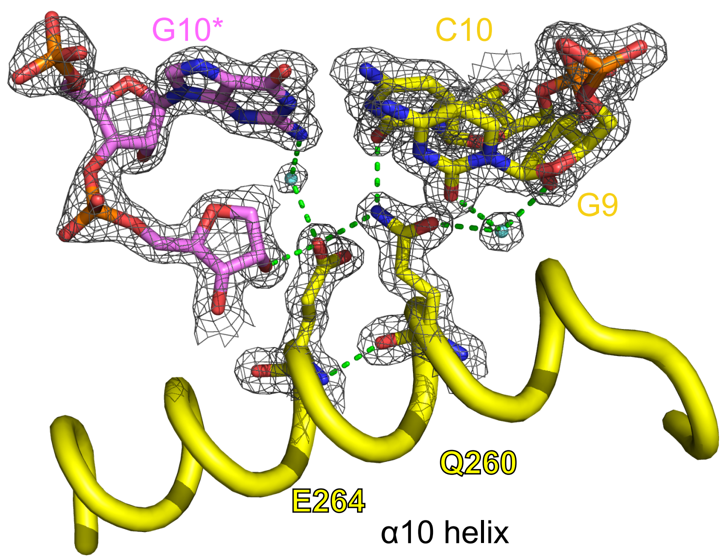
G10*
C10
G9
Q260
E264
α10 helix

B

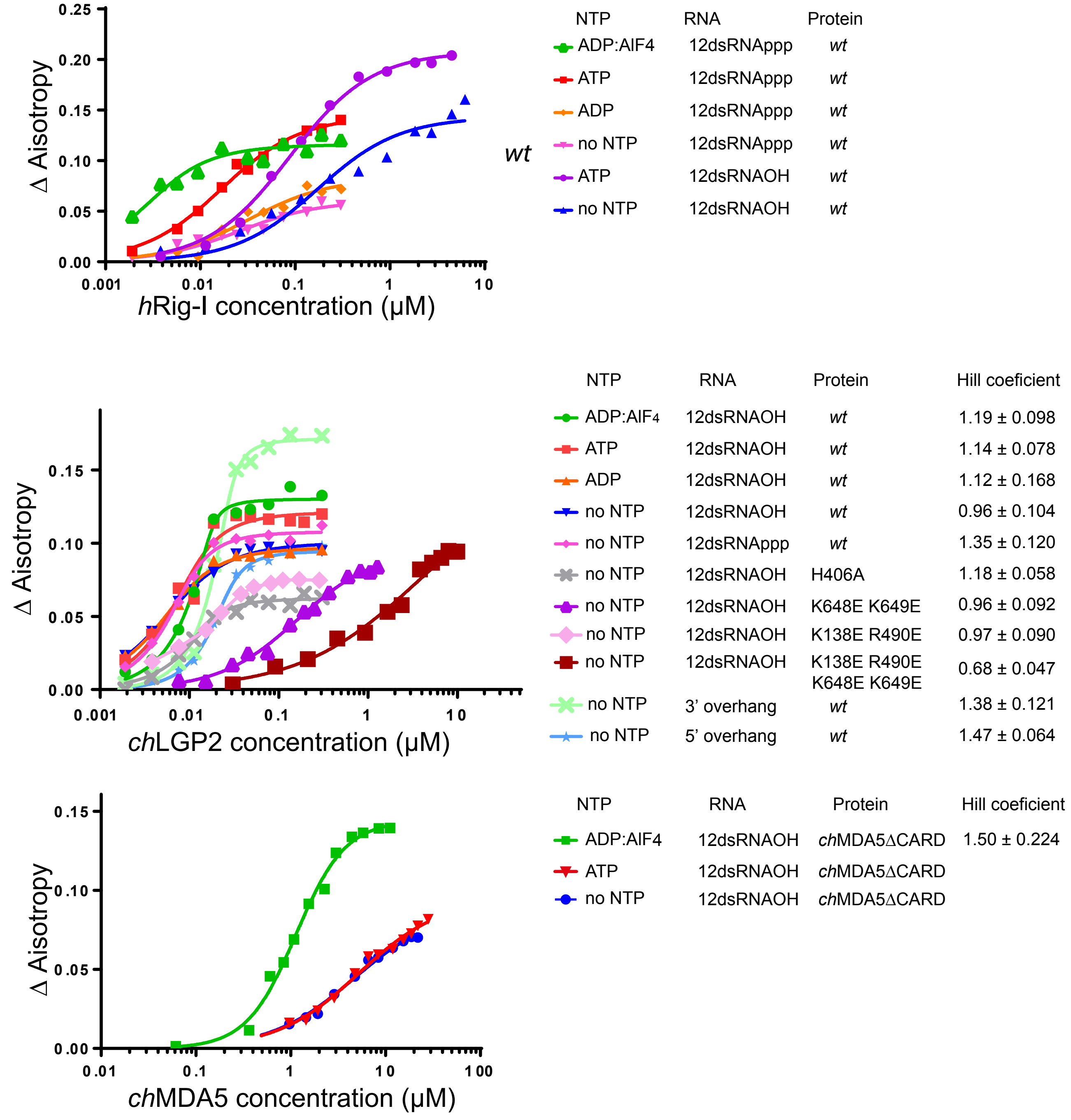
Δ Aisotropy
hRig-I concentration (μM)
wt
NTP RNA Protein
ADP:AlF4 12dsRNAppp wt
ATP 12dsRNAppp wt
ADP 12dsRNAppp wt
no NTP 12dsRNAppp wt
ATP 12dsRNAOH wt
no NTP 12dsRNAOH wt
chLGP2 concentration (μM)
NTP RNA Protein Hill coeficient
ADP:AlF4 12dsRNAOH wt 1.19 ± 0.098
ATP 12dsRNAOH wt 1.14 ± 0.078
ADP 12dsRNAOH wt 1.12 ± 0.168
no NTP 12dsRNAOH wt 0.96 ± 0.104
no NTP 12dsRNAppp wt 1.35 ± 0.120
no NTP 12dsRNAOH H406A 1.18 ± 0.058
no NTP 12dsRNAOH K648E K649E 0.96 ± 0.092
no NTP 12dsRNAOH K138E R490E 0.97 ± 0.090
no NTP 12dsRNAOH K138E R490E K648E K649E 0.68 ± 0.047
no NTP 3' overhang wt 1.38 ± 0.121
no NTP 5' overhang wt 1.47 ± 0.064
chMDA5 concentration (μM)
NTP RNA Protein Hill coeficient
ADP:AlF4 12dsRNAOH chMDA5ΔCARD 1.50 ± 0.224
ATP 12dsRNAOH chMDA5ΔCARD
no NTP 12dsRNAOH chMDA5ΔCARD

Figure S6

A

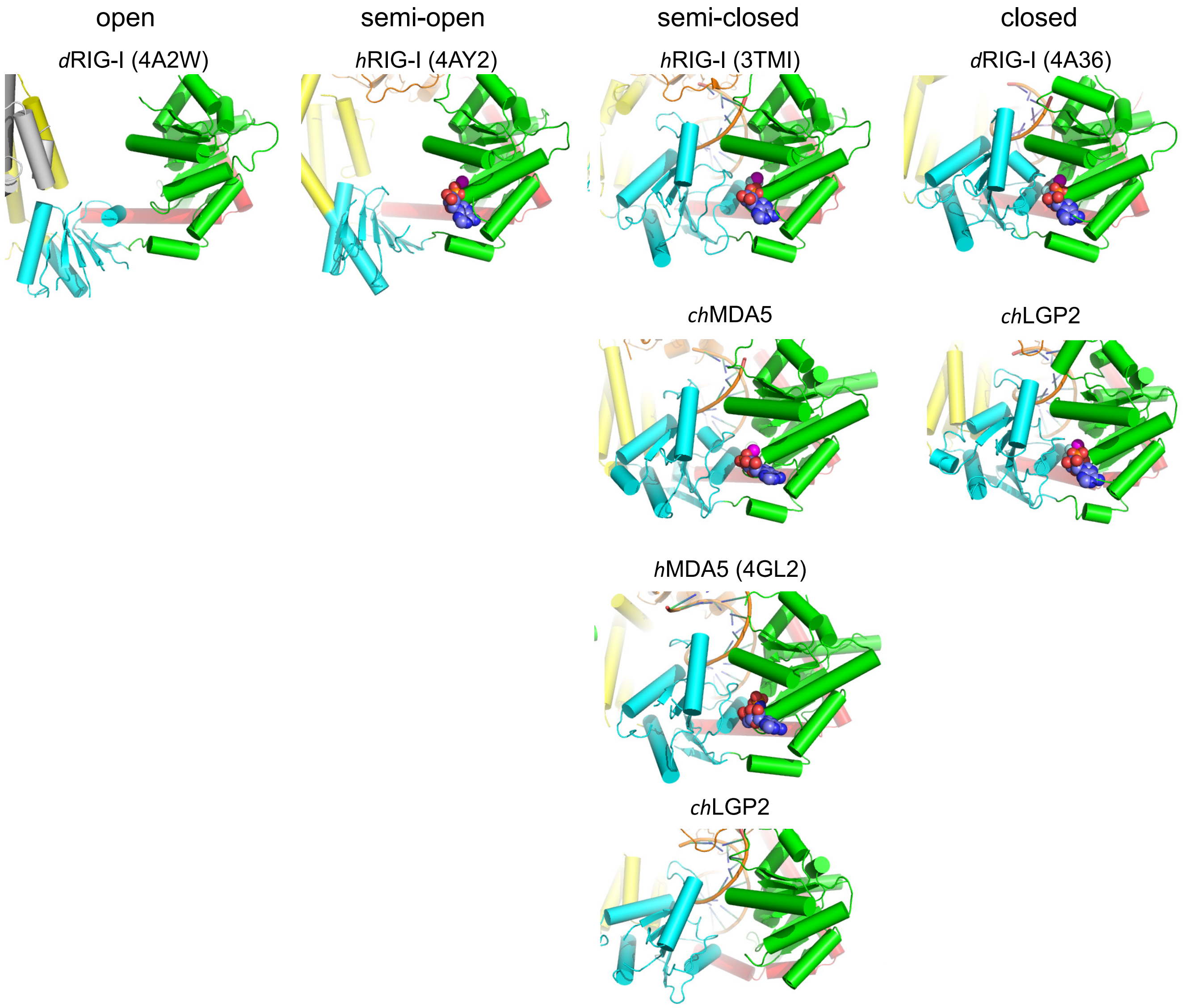

B

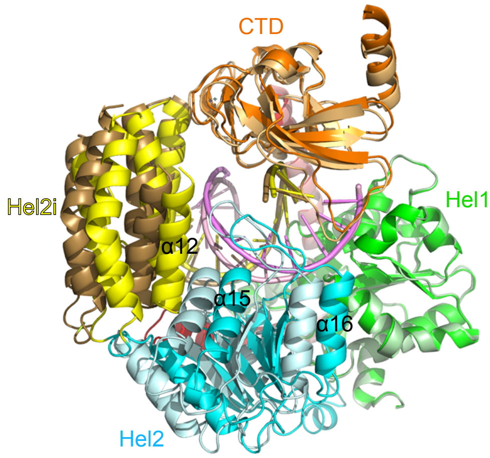

C

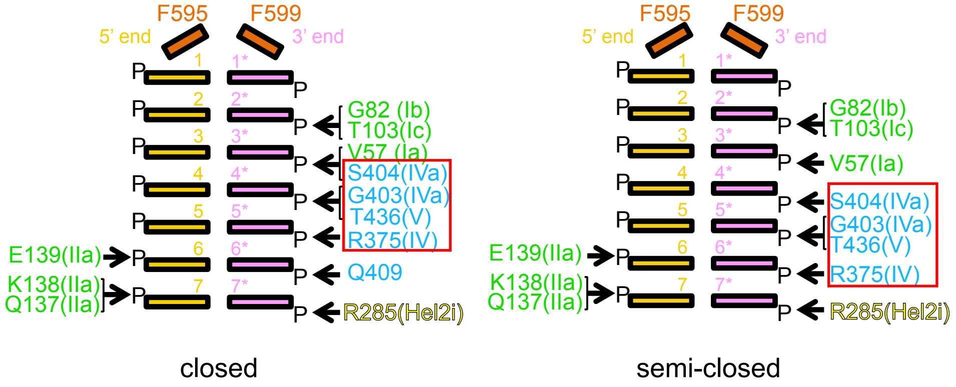

Figure S7

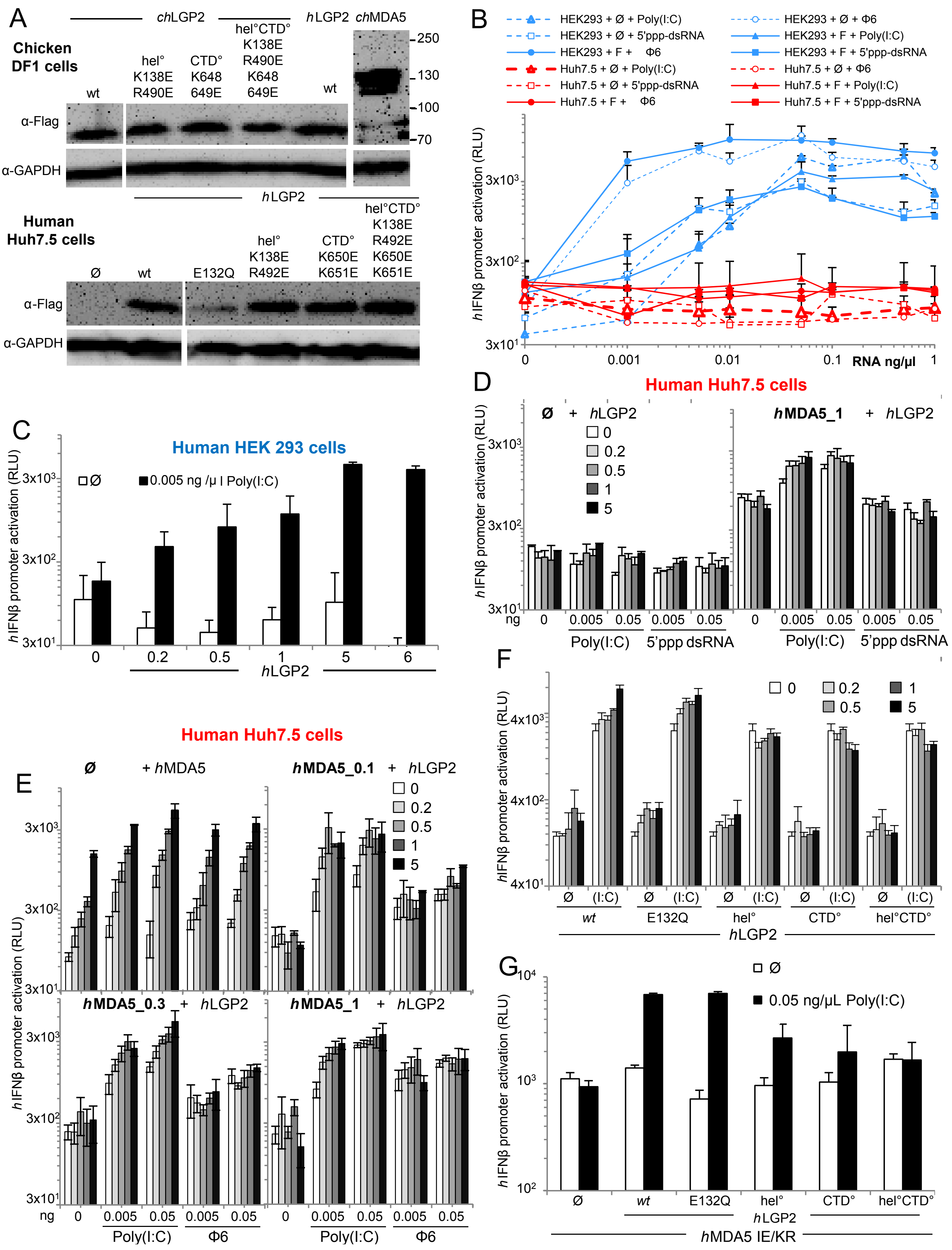

A
chLGP2
hLGP2 chMDA5
Chicken DF1 cells
wt
hel° K138E R490E
CTD° K648 649E
hel°CTD° K138E R490E K648 649E
wt
250
130
100
70
α-Flag
α-GAPDH
hLGP2
Human Huh7.5 cells
Ø
wt
E132Q
hel° K138E R492E
CTD° K650E K651E
hel°CTD° K138E R492E K650E K651E
B
HEK293 + Ø + Poly(I:C)
HEK293 + Ø + 5'ppp-dsRNA
HEK293 + F + Φ6
Huh7.5 + Ø + Poly(I:C)
Huh7.5 + Ø + 5'ppp-dsRNA
Huh7.5 + F + Φ6
HEK293 + Ø + Φ6
HEK293 + F + Poly(I:C)
HEK293 + F + 5'ppp-dsRNA
Huh7.5 + Ø + Φ6
Huh7.5 + F + Poly(I:C)
Huh7.5 + F + 5'ppp-dsRNA
hIFNβ promoter activation (RLU)
RNA ng/µl
C
Human HEK 293 cells
Ø
0.005 ng /µ l Poly(I:C)
hLGP2
D
Human Huh7.5 cells
Ø + hLGP2
hMDA5_1 + hLGP2
ng
Poly(I:C)
5'ppp dsRNA
E
Human Huh7.5 cells
Ø + hMDA5
hMDA5_0.1 + hLGP2
hMDA5_0.3 + hLGP2
hMDA5_1 + hLGP2
Φ6
F
wt
E132Q
hel°
CTD°
hel°CTD°
G
0.05 ng/µL Poly(I:C)
hMDA5 IE/KR

Table S1. Quantification of the degree of opening (rotation angle and center of mass distance) of the Hel1 and Hel2 helicase domains in published RLH structures. Related to Figures 1, 4 and S6.

**RLH Structure**	**PDB ID**	**RNA**	**Bound Nucleotide**	**Angle (degree)**	**Distance* (Å)**	**State**
*ch*LGP2 (Reference)	5JAJ	10 base pair 5′p	ADP:AlF_4	0.0	0.0	closed
*d*RIG-I	4A36	19 base pair 5′OH	ADP:AlF_4	2.5	1.3	closed
*h*MDA5	4GL2	12 base pair 5′OH	AMPPNP	8.0	3.3	semi-closed
*ch*MDA5	5JC3	10 base pair 5′p	ADP	10.1	3.9	semi-closed
*h*RIG-I	5E3H (3TMI)	14 base pair 5′OH	ADP:BeF_3	12.1	5.3	semi-closed
*ch*LGP2	5JBJ	12 base pair 5′p	-	13.1	5.4	semi-closed
*h*RIG-I	4AY2	20-mer hairpin 5′ppp	ADP	39.3	14.9	semi-open
*h*RIG-I	2YKG	10 base pair 5′OH	SO_4	40.3	15.9	open
*d*RIG-I	4A2W(A)	no RNA	-	51.8	18.5	open
*d*RIG-I	4A2W(B)	no RNA	-	59.8	19.2	open
*m*RIG-I	3TBK	no RNA	AMPPNP	68.2	24.4	open

Supplementary Experimental Procedures

Protein preparation

***ch*MDA5**. Chicken (*Gallus gallus*) MDA5 constructs (Genbank: NP_001180567) full-length *ch*MDA5 (residues 1-1001), *ch*MDA5_ΔCARD (residues 298-994), *ch*MDA5ΔCARD-Q (residues 298-994 with E436Q mutation) were cloned into pETM11 (EMBL) using a synthetic gene (GeneArt). All constructs were expressed in *E. coli* Rosetta 2 (Novagen) and cells were harvested and lysed by sonication in buffer containing 50 mM Tris pH 7.6, 500 mM NaCl, 10 % glycerol, 20 mM imidazole and 1 mM DTT. After centrifugation the lysate was applied to nickel-NTA-Superflow resin (QIAGEN). The protein was dialyzed against dialysis buffer (20 mM Hepes pH 7.5, 100 mM NaCl, 5% Glycerol, 1 mM DTT) at 4°C for 16 hours. The N-terminal 6-histidine-tag was cleaved with his-tagged Tobacco Etch Virus (TEV) protease during dialysis and cleaved protein repurified on nickel-NTA resin. *ch*MDA5 constructs were further purified with a cation exchange column (HiTrap SP, GE Healthcare) and size-exclusion chromatography (Superdex 200, GE Helthcare) in a buffer containing 20 mM Hepes pH 7.5, 150 mM NaCl and 2 mM DTT.

***ch*LGP2**. A synthetic gene (GeneArt) comprising the coding sequence of *ch*LGP2 (Genbank: AEK21509) with an added N-terminal GGGGS linker was cloned into pETM11SUMO (EMBL). Full-length *ch*LGP2 full length was expressed in *E.coli* Rosetta 2 as a Sumo fusion protein and cells were harvested and lysed by sonication in sonication buffer (50 mM Na_2HPO_4 pH 8, 300 mM NaCl, 5% glycerol, 50 mM arginine, 5 mM β-mercapto-ethanol). After centrifugation the lysate was applied to nickel-NTA-Superflow resin. The protein was dialyzed against sonication buffer at 4°C for 16 hours. The 6-histidine-tag and Sumo-tag was cleaved with TEV protease during dialysis. Cleaved protein was further purified with anion exchange column (HiTrap Q GE Healthcare) and size-exclusion chromatography (Superdex 200, GE Healthcare) in a buffer containing 20 mM Hepes pH 7.5, 200 mM NaCl and 250 µM TCEP (tris(2-carboxyethyl)phosphine).

***h*LGP2**. Full-length *h*LGP2 (Genbank NM_024119.2) was cloned into the pETM11SUMO vector (EMBL) and expressed in *E.coli* and purified as for *ch*LGP2. The chromatograph of a HiTrap Q purification shows two peaks, corresponding to *h*LGP2 monomers or dimers (the majority), as deduced from size-exclusion chromatography.

RIG-I. Full-length *h*RIG-I was cloned into pFastBAC and expressed in HiFive insect cells. Cells were harvested and lysed by sonication in buffer (50 mM Tris pH7.6, 500 mM NaCl, 10% glycerol, 20 mM Imidazole, 1 mM DTT). After centrifugation the lysate was applied to nickel-NTA-Superflow resin and further purified as for *ch*LGP2. Full-length *d*RIG-I was purified from insect cells as described (Kowalinski et al., 2011).

Crystallization

For protein crystallization, 576 different conditions were initially screened in vapour diffusion sitting drop format using a Cartesian robot. After screening, the crystal conditions were optimized in 24-well hanging drop crystallization plates.

***ch*MDA5 1:1 complex:** Directly after size exclusion chromatography *ch*MDA5ΔCARD-Q was mixed with 10 bp dsRNA in a 1:1 molar ratio and incubated for 30 minutes on ice. The complex was concentrated using an Amicon Ultra concentrator to around 10 mg/ml and 2 mM AMPPNP (adenosine 5′-(β,γ-imido)triphosphate lithium salt hydrate) was added. Sample and reservoir buffer (0.025 M Bis-Tris pH 6.5, 0.075 M succinic acid pH 7.0, 12-14% PEG 3350, 2% sucrose) were mixed in a 2:1 ratio. Three hours after setup, cover glasses with drops were transferred from a reservoir containing 12-14% PEG 3350 to one containing 8% PEG 3350. Crystals grew in one week at 20°C and were harvested in cryo-protectant solution (0.025 M Bis-Tris pH 6.5, 0.075 M succinic acid pH 7.0, 25% 3350, 10% ethylene glycol) before flash freezing with liquid nitrogen.

***ch*MDA5 2:1 complexes:** Directly after size exclusion chromatography *ch*MDA5ΔCARD-Q was mixed with 24, 26 or 27 bp dsRNA in a 0.5:1 molar ratio and incubated for 30 minutes

on ice. The complexes were concentrated with an Amicon Ultra concentrator to around 10 mg/ml and 2 mM AMPPNP and 200 mM NDSB211 (Dimethyl(2-hydroxyethyl)ammonium propane sulfonate, Hampton Research) were added.

***ch*MDA5ΔCARD-Q 24-mer dsRNA-AMPPNP** was mixed with reservoir buffer (0.1 mM Hepes pH 7.5, 11-12% PEG 3350, 2-4% sucrose) in a 2:1 ratio. Crystals grew in three days at 20 degree and were harvested in cryo-protecting solution (0.1 mM Hepes pH 7.5, 25% 3350, 10% ethylene glycol) before flash freezing with liquid nitrogen.

***ch*MDA5ΔCARD-Q : 27-mer dsRNA-AMPPNP** was mixed with reservoir buffer (0.1 mM Hepes pH 7.5, 4-6% PEG 6000, 2-4% sucrose) in a 2:1 ratio. Crystals grew in three days at 20°C. The cover glasses of crystal-containing drops were transferred to a reservoir containing cryo-protecting solution (0.1 mM Hepes pH 7.5, 10% PEG 6000, 25% ethylene glycol) 12 hours before harvesting and flash freezing with liquid nitrogen.

***ch*LGP2:** *ch*LGP2, directly after size exclusion chromatography, was mixed with dsRNA in a 1:1 ratio and incubated for 30 minutes on ice. The complexes were concentrated with an Amicon Ultra concentrator to around 10 mg/ml and then 2 mM ADP:AlF_4 (adenosine-5′-diphosphate: aluminium fluoride) and 2 mM $MgCl_2$ were added. All *ch*LGP2 crystals were harvested in the reservoir buffer containing 25% ethylene glycol and flash frozen with liquid nitrogen.

***ch*LGP2 12-mer dsRNA-ADP:AlF_4** complex was mixed with reservoir buffer (0.1 M Bis-Tris propane pH 6.5, 0.05-0.1 M magnesium formate) in a 2:1 ratio. Crystals grew in three days at 4°C.

***ch*LGP2 5′ monophosphate 10-mer dsRNA-ADP:AlF_4** complex was mixed with reservoir buffer (0.1 M tri-sodium citrate dihydrate pH 6.1, 0.04-0.08 M ammonium dihydrogen phosphate) in a 2:1 ratio. Crystals grew in one week at 4°C.

***ch*LGP2 5′ triphosphate 10-mer dsRNA-ADP:AlF_4** complex was mixed with reservoir

buffer (0.1 M Mg formate, 20% PEG3350M) in a 2:1 ratio. Crystals grew in one week at 4°C. **chLGP2 5′ triphosphate, 3′-GG overhang hairpin RNA-ADP:AlF4** complex was mixed with reservoir buffer (0.1 M citric acid pH 5.0, 0.8 M ammonium sulphate) in a 1:1 ratio. Crystals grew in one day at 4°C from a sitting drop 96 well plate.

Crystallography

Diffraction data were collected at 100 K on beamlines ID23-1 or ID29 equipped with a Pilatus 6M detector or ID23-2 equipped with a Pilatus 2M-F (DECTRIS), at the European Synchrotron Radiation Facility (ESRF), Grenoble, France. Data were processed with the XDS suite and scaled with XSCALE (Kabsch, 2010). All further analysis was done with the CCP4 suite (Winn et al., 2011) and refinement performed with REFMAC5 (Murshudov, 1997) or PHENIX (Adams et al., 2002). Models were built using COOT (Emsley and Cowtan, 2004), structure validation by MOLPROBITY (Chen et al., 2010) and structure figures drawn with PYMOL (DeLano, 2002). Buried surface are calculations performed with PISA (http://www.ebi.ac.uk/pdbe/prot_int/pistart.html) (Krissinel and Henrick, 2007). Crystallographic and refinement statistics are given in Tables 1 and 2.

The *ch*MDA5 structure was initially determined by molecular replacement with PHASER (McCoy et al., 2007) using the *h*MDA5 structure (PDB 4GL2). The *ch*LGP2 structure was initially determined with PHASER using the *h*LGP2 CTD (PDB: 2W4R) and RIG-I helicase sub-domains as search models.

Crystals of MDA5 with 10- and 24-mer dsRNAs were of highly variable quality, requiring testing of many to obtain usable data. For the 10-mer complex, several related space-groups were obtained with two ($P2_1$ or $P2_12_12_1$) or four (large $P2_1$) 1:1 complexes in the asymmetric unit. Both 10- and 24-mer crystals were generally twinned (probably lattice translocation disorder), this being manifested by weak but significant residual extra density after refinement that could be explained by the same model but referred to a different origin.

One 10-mer crystal in the $P2_1$ form was not twinned and gave a cleaner map and lower R-factors (Table 2).

RNA preparation

The palindromic 10 bp dsRNA with 5′ mono-phosphate or 5′ tri-phosphate (5′-pGGUACGUACC-3′ or 5′-pppGGUACGUACC-3′), 12 bp dsRNA with 5′ mono-phosphate (5′-pGGUAGCGCUACC-3′), 24 bp dsRNA with 5′ mono-phosphate (5′-pGGGACGUCAUGCGCAUGACGUCCC-3′) and 27 bp dsRNA with 5′ monophosphate (5′-pGGGCACGUGCAGGACCUGCACGUGCCC-3′) and 5′ triphosphate 3′-GG overhang 26-mer hairpin RNA (5′-pppGGAGCGUGCCGUACGGCACGCUCCGG-3′) were prepared by *in vitro* T7 transcription. Five times excess of GMP over GTP was added to the reaction for the 5′ mono-phosphate RNAs. RNA was purified by excising the band from denaturing urea-PAGE. A summary of RNAs used is shown in Figure S4.

Electron microscopy

Φ6 RNA (Thermo Scientific) is a mixture of 2948 bp (S segment), 4063 bp (M), and 6374 bp (L) dsRNAs. To form complexes, full-length *ch*MDA5 or *ch*LGP2 and Φ6 bacteriophage dsRNA were incubated during 5 min at 20°C in a buffer containing 20 mM Hepes pH 7.5, 150 mM NaCl, 2 mM ATP and 2 mM $MgCl_2$. Different protein:RNA ratios were used with a 1:1 ratio of MDA5/LGP2:dsRNA corresponding to one MDA5/LGP2 molecule for 15 bp of dsRNA. Where indicated, ATP was replaced by ADP, ADP:AlF_4 or no NTP analogues in order to test their influence.

For each condition tested, 4 µl of sample was applied to the clear side of carbon on a carbon-mica interface and stained with 1% (wt/vol) uranyl acetate. Images were recorded under low-dose conditions with a JEOL 1200 EX II microscope at 100 kV with a nominal magnification of 15,000×.

Cryo-EM grids were prepared by mixing 4 µl of MDA5 or LGP2 at 5 µM with

dsRNA in a 1:1 ratio. The sample was applied onto a glow-discharged quantifoil grid 400 mesh 3.5/1 (Quantifoil Micro Tools GmbH, Germany), the excess solution was blotted for 2 s with a Vitrobot (FEI) and the grid frozen in liquid ethane. Data collection was performed on a FEI Polara microscope operated at 300 kV. Fifty cryoEM micrographs were manually collected on a K2 summit electron direct detector (Gatan) at a magnification of 20,000×, giving a pixel size of 0.97 Å. Movies of 6 frames were collected with a total exposure time of 6 s and a total dose of $30e^{-}/Å^{2}$. The contrast transfer function (CTF) for each micrograph was determined with CTFFIND3 (Mindell and Grigorieff, 2003) and showed defocuses between 2.2 and 3.8 µm. A total of 7185 overlapping segments of MDA5 and 3153 overlapping segments of LGP2 (400 pixels long) were picked using EMAN boxer (Ludtke et al., 1999) with a shift of 64 pixels between adjacent segments. Corresponding segments were suggested to 2D classification in Relion1.3 (Scheres, 2012). Power spectra were calculated from masked 2D class averages.

Fluorescence polarisation anisotropy

dsRNAs used for anisotropy experiments were formed by annealing the following separate strands for 1 min at 95 °C and cooling down on ice in a buffer containing 20 mM Hepes, 2 mM EDTA at pH 6.8 (see also Figure S5 for RNA sequences):

12-mer dsRNA : 5′-$_{FAM}$CAUGUGGAGCCC-3′ and 5′-$_{OH}$GGGCUCCACAUG-3′,

12-mer dsRNAppp : 5′-$_{FAM}$CAUGUGGAGCCC-3′ and 5′-$_{ppp}$GGGCUCCACAUG-3,

3′ overhang dsRNA : 5′-$_{OH}$GGCCAGUGCGAA-3′ and 5′-$_{FAM}$CGCACUGGCCAA-3′,

5′ overhang dsRNA : 5′-$_{OH}$AACCGGUCACGC3′ and 5′-$_{OH}$AAGCGUGACCGG$_{FAM}$-3′.

Anisotropy measurements were performed at room temperature using a multimodular fluorometer (Quantamaster QM4CW; Manufacturer: Photon Technology International). RNA concentration was adjusted to 2.5 nM (12-mer 5′ppp-dsRNA) or 5 nM (12-mer 5′OH-dsRNA, 3′ 2 nt overhang dsRNA and 5′ 2 nt overhang dsRNA for LGP2 measurements), 13

or 50 nM (12-mer 5′OH-dsRNA for MDA5 measurements) by mixing in the cuvette with buffer (20 mM Hepes pH 7.5 150 mM NaCl, 200 µM TCEP, 4 mM $MgCl_2$). Protein was titrated into the cuvette and incubated for 5 minutes before measurements. The total volume of titrated protein was less than 10% of the final volume. Anisotropy measurements were made with excitation wavelength 495 nm and emission wavelength 515 nm for 100 seconds. Experiments were performed more than three times per system. Binding data was analysed by Graph Pad Prism software and fitted with the following equation (eq. 1).

$$y = \frac{(\mathrm{R}+P^n+Kd^n)-\sqrt{(R+P^n+kd^n)^2-4RP^n}}{2R} \times b + (m-b) \qquad \text{(eq. 1)}$$

The experimental anisotropy (y), Initial anisotropy (b), the maximum anisotropy (m), the RNA concentration (R), and the protein concentration (P), equilibrium binding constant (*K*d) and hill coefficient (n).Rig-I measurement curves are fitted with n=1 and the value of n are written in Figure S4. The error bars represent the S.D. value of at least three individual experiments.

ATPase activity assays

ATPase reactions were monitored using a Malachite green assay kit (Bioassays) over 0.5-30 min time courses. All proteins were pre-incubated with 4-fold molar excess of dsRNA for 10 minutes at 28 °C in ATP hydrolysis buffer (20 mM Hepes pH7.5, 100 mM NaCl, 1% glycerol, 2 mM DTT, 4 mM $MgCl_2$). The reaction was initiated by adding 2 mM ATP. For determination of kinetic parameters, the ATP concentration was varied from 0.125 to 10 mM. The reactions were quenched at 5 time points between 30 seconds to 4 minutes. The 10 µl reaction aliquots were quenched by mixing with 10 µl of quenching buffer (20 mM Hepes pH 7.5, 100 mM NaCl, 1% glycerol, 2 mM DTT, 100 mM EDTA). 80 µl of 5 times diluted Malachite green solution was added and developed for 30 minutes at room temperature. The absorbance at 622 nm was measured with a plate reader. For each ATP concentration, the

initial velocity was plotted and *K*m and *k*cat calculated using the Michaelis–Menten equation (eq. 2) in Graph Pad software Prism.

Y=Et*kcat*X/(*K*m+X) (eq. 2)

X is the substrate concentration, Y is enzyme velocity and Et is the concentration of enzyme.

Cellular assays.

Myc-*h*MDA5 and Flag-*h*RIG-I constructs in pEF-BOS vector have been described previously (Kowalinski et al., 2011; Louber et al., 2015; Louber et al., 2014) and *ch*MDA5, *ch*LGP2, *h*LGP2 tagged at the N terminus with Flag peptide were sub-cloned into pCDNA3 (chicken) and pEF-BOS (human) vector as detailed elsewhere (Louber et al., 2015; Louber et al., 2014).

Chicken DF1 (Himly et al., 1998) and human Huh7.5 (Sumpter et al., 2005) cells were maintained in Dulbecco's Minimum Essential Medium culture medium supplemented with 10% foetal calf serum, 10 mM HEPES, 2 mM L-glutamine, 10 mg/ml gentamycin and 1% non-essential amino acids for Huh7.5 cells at 37°C and 5% CO2. Being of chicken origin, DF1 cells naturally lack endogenous RIG-I (Barber et al., 2010), but express functional endogenous MDA5, LGP2 and type I IFN system (Karpala et al., 2011; Liniger et al., 2012). Huh7.5 cell line is defective in MDA5 and IFN receptor expression and expresses inactive T55I RIG-I (Binder et al., 2011; Eguchi et al., 2000; Keskinen et al., 1999; Li et al., 2005; Sumpter et al., 2005), a phenotype verified experimentally by the inability of these cells to respond to both RIG-I and MDA5 RNA agonists (Figure S7 and our previous data (Louber et al., 2015)).

Protein expression was analysed after lysis of DF1 and Huh7.5 cells (6-well plate, 6x105 cells/well) transfected the day before with 2.5 µg DNA using TransIT®-LT1 Transfection Reagent (Mirus Bio) (7.5 µl/well), separation by polyacrylamide gel

electrophoresis and visualisation by western immunoblotting using anti-Flag (1:1,000; M2; Sigma, St. Louis, MO, USA), anti-C-Myc (1:50) 9E10, anti-GAPDH (1:2,000; Millipore, Billerica, MA, USA) monoclonal antibodies

The human IFNβ promoter luciferase assay was performed essentially as described (Louber et al., 2015; Louber et al., 2014). Briefly, Huh7.5 cells seeded one day before (96 wells, 2 x104 cells/well) were co-transfected with MDA5 and LGP2 expressing vectors (33 ng of DNA/well to which was added 17 ng of DNA/well of pHRL-tk-Renilla-Luc (Promega)) together with the reporter pβIFN-fl-lucter (King and Goodbourn, 1994) (50 ng of DNA/well), using TransIT®-LT1 Transfection Reagent (Mirus Bio) (0.3 µl /well), followed 24 hours later by transfection of either poly(I:C) (Amersham Biosciences), Φ6 dsRNA (Thermo Scientific) or T7-transcribed 61-mer 5′ppp-dsRNA (Marq et al., 2011) in complex with Oligofectamine™ Transfection Reagent (Invitrogen) (0.6 µl/well). The chicken IFNβ promoter luciferase assay was performed following exactly the same procedure, but using chicken adapted material, i.e. DF1 cells, pCDNA3 based expression vectors for MDA5 and LGP2, pHRL-CMV-Renilla-Luc (Promega) instead of pHRL-tk-Renilla-Luc, the reporter pGL3-P_*ch*IFNβ-Luc (Liniger et al., 2012). For both human and chicken assay, control experiments were included by transfecting the same amount of a neutral plasmid (pCG-duF) (Cathomen et al., 1995). This plasmid was also used to complement transfected DNA so as to reach total DNA amount of 100 ng per well. Data were expressed as mean +/-SD of normalized luciferase activity. Statistical analysis (Student t test) was done on data from three independent experiments, each done in independent triplicates.

Supplementary References

Adams, P.D., Grosse-Kunstleve, R.W., Hung, L.W., Ioerger, T.R., McCoy, A.J., Moriarty, N.W., Read, R.J., Sacchettini, J.C., Sauter, N.K., and Terwilliger, T.C. (2002). PHENIX: building new software for automated crystallographic structure determination. Acta Crystallogr D Biol Crystallogr *58*, 1948-1954.

Barber, M.R., Aldridge, J.R., Jr., Webster, R.G., and Magor, K.E. (2010). Association of RIG-I with innate immunity of ducks to influenza. Proc Natl Acad Sci U S A *107*, 5913-5918.

Binder, M., Eberle, F., Seitz, S., Mucke, N., Huber, C.M., Kiani, N., Kaderali, L., Lohmann, V., Dalpke, A., and Bartenschlager, R. (2011). Molecular mechanism of signal perception and integration by the innate immune sensor retinoic acid-inducible gene-I (RIG-I). The Journal of biological chemistry *286*, 27278-27287.

Cathomen, T., Buchholz, C.J., Spielhofer, P., and Cattaneo, R. (1995). Preferential initiation at the second AUG of the measles virus F mRNA: a role for the long untranslated region. Virology *214*, 628-632.

Chen, V.B., Arendall, W.B., 3rd, Headd, J.J., Keedy, D.A., Immormino, R.M., Kapral, G.J., Murray, L.W., Richardson, J.S., and Richardson, D.C. (2010). MolProbity: all-atom structure validation for macromolecular crystallography. Acta Crystallogr D Biol Crystallogr *66*, 12-21.

DeLano, W.L. (2002). PyMOL Molecular Graphics System. available online at http://wwwpymolsourceforgenet.

Eguchi, H., Nagano, H., Yamamoto, H., Miyamoto, A., Kondo, M., Dono, K., Nakamori, S., Umeshita, K., Sakon, M., and Monden, M. (2000). Augmentation of antitumor activity of 5-fluorouracil by interferon alpha is associated with up-regulation of p27Kip1 in human hepatocellular carcinoma cells. Clinical cancer research : an official journal of the American Association for Cancer Research *6*, 2881-2890.

Emsley, P., and Cowtan, K. (2004). Coot: model-building tools for molecular graphics. Acta Crystallogr D Biol Crystallogr *60*, 2126-2132.

Gouet, P., Courcelle, E., Stuart, D.I., and Metoz, F. (1999). ESPript: analysis of multiple sequence alignments in PostScript. Bioinformatics (Oxford, England) *15*, 305-308.

Himly, M., Foster, D.N., Bottoli, I., Iacovoni, J.S., and Vogt, P.K. (1998). The DF-1 chicken fibroblast cell line: transformation induced by diverse oncogenes and cell death resulting from infection by avian leukosis viruses. Virology *248*, 295-304.

Kabsch, W. (2010). Xds. Acta Crystallogr D Biol Crystallogr *66*, 125-132.

Karpala, A.J., Stewart, C., McKay, J., Lowenthal, J.W., and Bean, A.G. (2011). Characterization of chicken Mda5 activity: regulation of IFN-beta in the absence of RIG-I functionality. Journal of immunology *186*, 5397-5405.

Keskinen, P., Nyqvist, M., Sareneva, T., Pirhonen, J., Melen, K., and Julkunen, I. (1999). Impaired antiviral response in human hepatoma cells. Virology *263*, 364-375.

King, P., and Goodbourn, S. (1994). The beta-interferon promoter responds to priming through multiple independent regulatory elements. The Journal of biological chemistry *269*, 30609-30615.

Kowalinski, E., Lunardi, T., McCarthy, A.A., Louber, J., Brunel, J., Grigorov, B., Gerlier, D., and Cusack, S. (2011). Structural basis for the activation of innate immune pattern-recognition receptor RIG-I by viral RNA. Cell *147*, 423-435.

Krissinel, E., and Henrick, K. (2007). Inference of macromolecular assemblies from crystalline state. Journal of molecular biology *372*, 774-797.

Li, K., Chen, Z., Kato, N., Gale, M., Jr., and Lemon, S.M. (2005). Distinct poly(I-C) and virus-activated signaling pathways leading to interferon-beta production in hepatocytes. The Journal of biological chemistry *280*, 16739-16747.

Liniger, M., Summerfield, A., Zimmer, G., McCullough, K.C., and Ruggli, N. (2012). Chicken cells sense influenza A virus infection through MDA5 and CARDIF signaling involving LGP2. Journal of virology *86*, 705-717.

Louber, J., Brunel, J., Uchikawa, E., Cusack, S., and Gerlier, D. (2015). Kinetic discrimination of self/non-self RNA by the ATPase activity of RIG-I and MDA5. BMC biology *13*, 54.

Louber, J., Kowalinski, E., Bloyet, L.M., Brunel, J., Cusack, S., and Gerlier, D. (2014). RIG-I Self-Oligomerization Is Either Dispensable or Very Transient for Signal Transduction. PloS one *9*, e108770.

Ludtke, S.J., Baldwin, P.R., and Chiu, W. (1999). EMAN: semiautomated software for high-resolution single-particle reconstructions. J Struct Biol *128*, 82-97.

Marq, J.B., Hausmann, S., Veillard, N., Kolakofsky, D., and Garcin, D. (2011). Short double-stranded RNAs with an overhanging 5' ppp-nucleotide, as found in arenavirus genomes, act as RIG-I decoys. The Journal of biological chemistry *286*, 6108-6116.

McCoy, A.J., Grosse-Kunstleve, R.W., Adams, P.D., Winn, M.D., Storoni, L.C., and Read, R.J. (2007). Phaser crystallographic software. J Appl Crystallogr *40*, 658-674.

Mindell, J.A., and Grigorieff, N. (2003). Accurate determination of local defocus and

specimen tilt in electron microscopy. J Struct Biol *142*, 334-347.

Murshudov, G.N. (1997). Refinement of macromolecular structures by the maximum-likelihood method. Acta Crystallogr D Biol Crystallogr *53*, 240-255.

Scheres, S.H. (2012). RELION: implementation of a Bayesian approach to cryo-EM structure determination. J Struct Biol *180*, 519-530.

Sumpter, R., Jr., Loo, Y.M., Foy, E., Li, K., Yoneyama, M., Fujita, T., Lemon, S.M., and Gale, M., Jr. (2005). Regulating intracellular antiviral defense and permissiveness to hepatitis C virus RNA replication through a cellular RNA helicase, RIG-I. Journal of virology *79*, 2689-2699.

Winn, M.D., Ballard, C.C., Cowtan, K.D., Dodson, E.J., Emsley, P., Evans, P.R., Keegan, R.M., Krissinel, E.B., Leslie, A.G., McCoy, A., *et al.* (2011). Overview of the CCP4 suite and current developments. Acta Crystallogr D Biol Crystallogr *67*, 235-242.
